# HIV-1 Subtype B Protease and Reverse Transcriptase Amino Acid Covariation

**DOI:** 10.1371/journal.pcbi.0030087

**Published:** 2007-05-11

**Authors:** Soo-Yon Rhee, Tommy F Liu, Susan P Holmes, Robert W Shafer

**Affiliations:** 1 Division of Infectious Diseases, Department of Medicine, Stanford University, Stanford, California, United States of America; 2 Department of Statistics, Stanford University, Stanford, California, United States of America; Lilly Systems Biology, Singapore

## Abstract

Despite the high degree of HIV-1 protease and reverse transcriptase (RT) mutation in the setting of antiretroviral therapy, the spectrum of possible virus variants appears to be limited by patterns of amino acid covariation. We analyzed patterns of amino acid covariation in protease and RT sequences from more than 7,000 persons infected with HIV-1 subtype B viruses obtained from the Stanford HIV Drug Resistance Database (http://hivdb.stanford.edu). In addition, we examined the relationship between conditional probabilities associated with a pair of mutations and the order in which those mutations developed in viruses for which longitudinal sequence data were available. Patterns of RT covariation were dominated by the distinct clustering of Type I and Type II thymidine analog mutations and the Q151M-associated mutations. Patterns of protease covariation were dominated by the clustering of nelfinavir-associated mutations (D30N and N88D), two main groups of protease inhibitor (PI)–resistance mutations associated either with V82A or L90M, and a tight cluster of mutations associated with decreased susceptibility to amprenavir and the most recently approved PI darunavir. Different patterns of covariation were frequently observed for different mutations at the same position including the RT mutations T69D versus T69N, L74V versus L74I, V75I versus V75M, T215F versus T215Y, and K219Q/E versus K219N/R, and the protease mutations M46I versus M46L, I54V versus I54M/L, and N88D versus N88S. Sequence data from persons with correlated mutations in whom earlier sequences were available confirmed that the conditional probabilities associated with correlated mutation pairs could be used to predict the order in which the mutations were likely to have developed. Whereas accessory nucleoside RT inhibitor–resistance mutations nearly always follow primary nucleoside RT inhibitor–resistance mutations, accessory PI-resistance mutations often preceded primary PI-resistance mutations.

## Author Summary

The identification of which mutations in a protein covary has played a major role in both structural and evolutionary biology. Covariation analysis has been used to help predict unsolved protein structures and to better understand the functions of proteins with known structures. The large number of published genetic sequences of the targets of HIV-1 therapy has provided an unprecedented opportunity to identify dependencies among mutations in these proteins that can be exploited to design inhibitors that have high genetic barriers to resistance. In our analysis, we identified many pairs of covarying drug-resistance mutations in HIV-1 protease and reverse transcriptase and organized them into clusters of mutations that often develop in a predictable order. Inhibitors that are active against early drug-resistant mutants are likely to be less prone to the development of resistance, whereas inhibitors that are active against fully evolved clusters of mutations may be useful drugs for salvage therapy.

## Introduction

HIV-1 is a highly mutable pathogen. In the decades since it entered human populations, it has accumulated extensive sequence variation leading to the development of different subtypes and recombinant forms [1]. Although the enzymatic targets of therapy are among the most conserved parts of the HIV-1 genome, these too can develop marked variation, particularly in the setting of selective antiretroviral drug pressure. Indeed, it is not uncommon for drug therapy to select for protease and reverse transcriptase (RT) variants containing substitutions at more than 10% of their amino acids [2]. However, despite this high degree of mutation, the spectrum of possible virus variants appears to be limited by patterns of amino acid covariation.

In 2003, we published two studies that examined the extent of covariation among RT and protease residues in the presence and absence of antiretroviral therapy [3,4]. Despite the relatively large size of the datasets in these studies—2,244 protease sequences and 1,210 RT sequences—there were insufficient data to examine patterns of covariation of different mutations at the same position. As more sequence data have become available, we are now analyzing covariation among mutations (rather than positions) in protease and RT. This expanded analysis uses a highly specific measure of covariation, the Jaccard similarity coefficient, and a multidimensional scaling based on this coefficient. In addition, we examine the relationship between conditional probabilities associated with a mutation pair and the order in which those mutations develop in viruses for which longitudinal sequence data are available.

## Results

### Protease

Protease sequences from 3,982 protease inhibitor (PI)–naive individuals and from 3,475 PI-experienced individuals were available for analysis. The PI-experienced individuals had received a median of 1 PI (interquartile range, 1–3).

Jaccard similarity coefficients and their standardized Z scores were calculated for all pairs of mutations at different positions present three or more times among the sequences from PI-naive and PI-experienced individuals. Among 19,203 pairs of mutations from the PI-experienced individuals, 161 pairs were significantly associated after adjusting for multiple comparisons by controlling the family-wise error rate at <0.01. Of these 161 pairs, 92 (57%) were positively associated (Z > 5.1, unadjusted *p* < 4.4 × 10^−7^) and 69 (43%) were negatively associated (Z < −5.0, unadjusted *p* < 4.8 × 10^−7^). Table 1 shows the Jaccard similarity coefficients and conditional probabilities of the 40 strongest positively associated protease mutation pairs and the ten strongest negatively associated protease mutation pairs. Table S1 shows the complete list of 161 statistically significant mutation pairs.

**Table 1:**
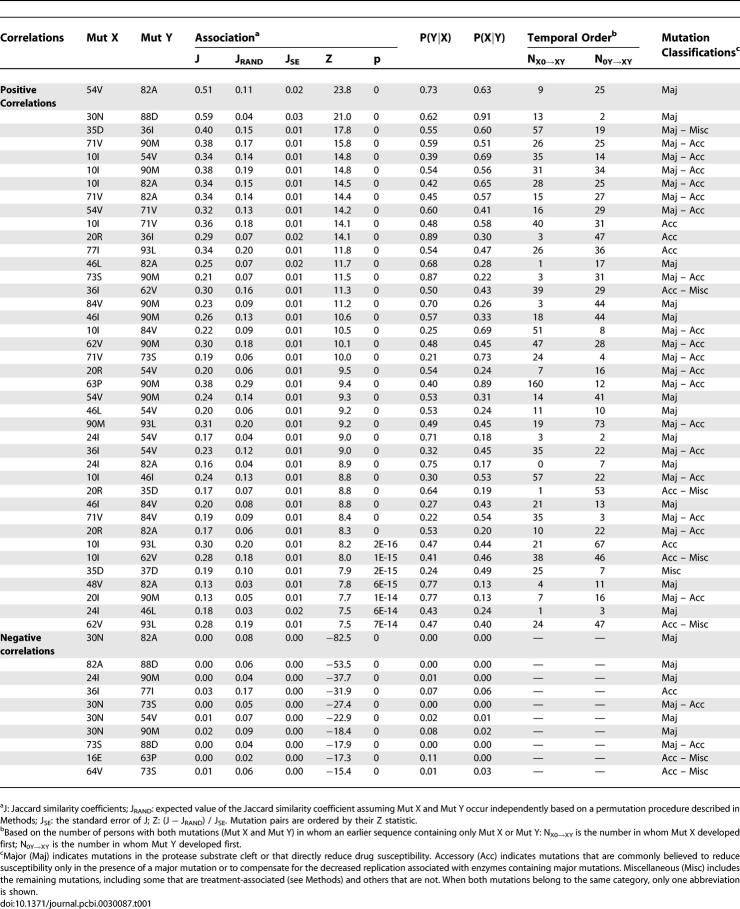
Forty Highest Positively Correlated Protease Mutation Pairs and Ten Highest Negatively Correlated Protease Mutation Pairs from PI-Experienced Persons

For the positively associated mutation pairs, Table 1 also contains two columns with data on the temporal order in which correlated mutations occurred in sequences with both mutations from persons in which an earlier sequence was available that contained only one of the two mutations. For example, the first row shows that among persons with both I54V and V82A in whom an earlier sequence contained only one of these two mutations was available, I54V occurred first in nine (26%) of 34 people, and V82A occurred first in 25 (74%) of 34 people (*p* < 0.01). In contrast, the fourth row shows that among persons with both A71V and L90M, each of the mutations was as likely to occur first (26 of 51 versus 25 of 51; *p* = NS). Figure S1 plots the relationship between the log of the ratio of the conditional probability of two mutations versus the log of the ratio in which two mutations develop, indicating that the conditional dependence between mutations is highly correlated with the order in which the mutations develop when they occur together (r^2^ = 0.56, *p* < 0.001).

Among the 18 positively associated pairs in Table 1 containing a major and an accessory PI-resistance mutation (as defined in Methods), the accessory mutation appeared first more often in 12 of the 18 pairs. There were several striking patterns of temporal association among these 18 pairs of correlated major and accessory mutations. The major mutation L90M preceded the accessory mutation G73S in 31 of 34 persons for whom temporal data were available. In contrast, the accessory mutation L63P preceded L90M in 160 of 172 persons, and the accessory mutations L10I and A71V preceded the major mutation I84V in 51 of 59 and 35 of 38 persons, respectively.

**Figure 1:**
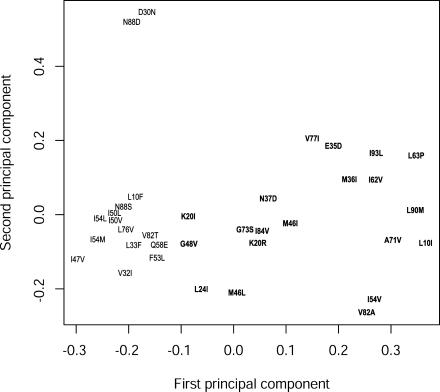
Multidimensional Scaling of 35 HIV-1 Protease Mutations Includes the 22 mutations obtained from the mutation pairs with the highest positive association (Table 1) in bold, and 13 additional clinically relevant protease inhibitor resistance mutations (L10F, V32I, L33F, I47V, I50V/L, F53L, I54L/M, Q58E, L76V, V82T, and N88S). The graph is a 2-D projection of the distances among the 35 mutations, in which the distance between any two mutations is measured by their Jaccard dissimilarity coefficient among persons who have received at least one protease inhibitor.

The Jaccard dissimilarity coefficients associated with 595 pairs of 35 mutations were used for a multidimensional scaling. The mutations included in this analysis were the 22 positively associated mutations in Table 1 and 13 additional clinically relevant PI-resistance mutations (L10F, V32I, L33F, I47V, I50V/L, F53L, I54L/M, Q58E, L76V, V82T, and N88S). Figure 1 plots the mutations along axes representing the first two principal components. The first principal component accounted for 10% of the total inertia and separates the nelfinavir-resistance mutations D30N and N88D from the main group of PI-resistance mutations. The second principal component accounted for 7% of the total inertia and separates V82A-associated mutations (I54V, L24I, and M46L) from L90M-associated mutations (M46I, G73S, and I84V). Finally, the lower-left part of the figure contains a cluster with seven of the 11 mutations recently reported to be associated with phenotypic and clinical resistance to the newest PI, darunavir (V32I, L33F, I47V, I50V, I54L/M, and L76V).

At several positions, there was sufficient data to contrast covariation patterns for different mutations. For example, M46I/L were each significantly associated with L10I, L24I, V32I, L33F, I54V, V82A, and L90M. However, M46I was uniquely associated with F53L, G73S/T, V82F/T, I84V, and N88S. I54V was significantly associated with L10F, L24I, L33F, M46I/L, G48V, F53L, V82A/F/T, I84V, and L90M. In contrast, I54L/M were significantly associated only with L33F, M46I, I47V, I84V, and L90M. N88D was positively associated with D30N and negatively associated with M46I, whereas N88S was negatively associated with D30N and positively associated with M46I. Of note, the divergent associations of different mutations at positions 46 and 88 have previously been reported by Hoffman and coworkers [5].

Among 7,131 pairs of mutations in sequences from PI-naive persons, 65 pairs were significantly associated (family-wise error rate < 0.01; Table S2). All but three of the positive associations among PI-naive persons were weaker (i.e., had a lower Z score) than the positive associations among treated persons in Table 1.

### Reverse Transcriptase

RT sequences from 2,601 RT inhibitor–naive and from 5,188 RT inhibitor–experienced individuals were available for analysis. The RT inhibitor experienced individuals had received a median of three nucleoside RT inhibitors (NRTIs; interquartile range, 2–4) and zero nonnucleoside RT inhibitors (NNRTIs; interquartile range, 0–1).

Jaccard similarity coefficients and their standardized Z scores were calculated for all pairs of RT mutations at different positions present three or more times among the sequences from RT inhibitor–experienced and –naive persons. Among 65,624 pairs of mutations from the RT inhibitor–experienced persons, 327 pairs were significantly associated after adjusting for multiple comparisons by controlling the family-wise error rate at <0.01. Of these 327 pairs, 213 (65%) were positively associated (Z > 5.2, unadjusted *p* < 2 × 10^−7^) and 114 (35%) were negatively associated (Z < −5.0, unadjusted *p* < 5 × 10^−7^). Table 2 shows the Jaccard similarity coefficients and conditional probabilities of the 40 strongest positively associated RT mutation pairs and the ten strongest negatively associated RT mutation pairs. Table S3 shows the complete list of 327 statistically significant RT mutation pairs.

**Table 2:**
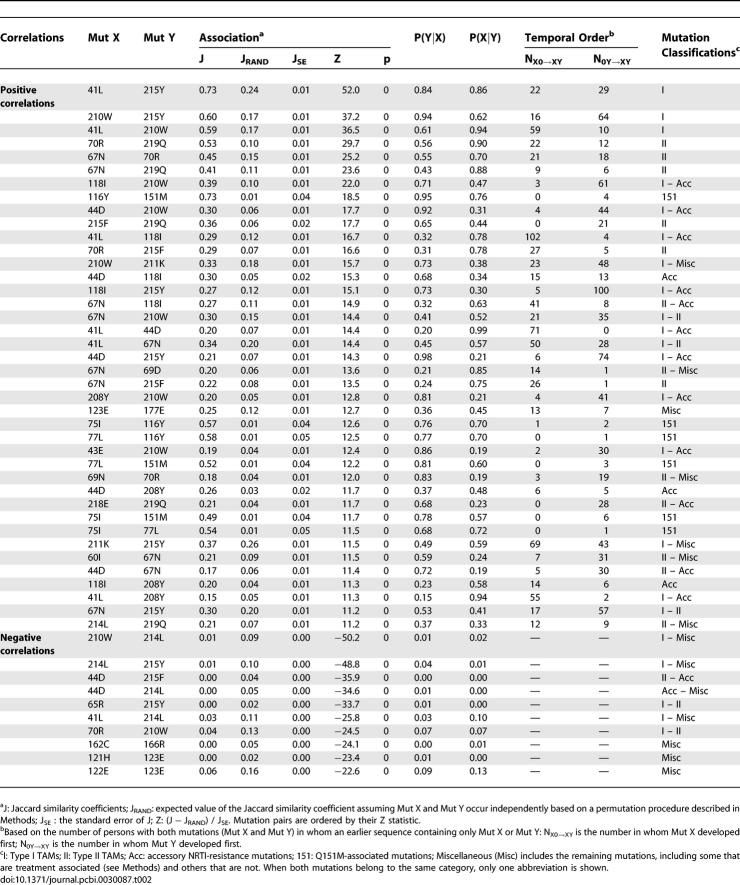
Forty Highest Positively Correlated RT Mutation Pairs and Ten Highest Negatively Correlated RT Mutation Pairs from RTI-Experienced Persons

Positively associated mutation pairs consisted primarily of Type I or II thymidine analog mutations (TAMs; as defined in Methods); accessory NRTI mutations that occurred in combination with Type I or II TAMs (K43E, E44D, V118I, H208Y, D218E); and Q151M-associated mutations (V75I, F77L, F116Y). Among the top 40 associated mutation pairs, there were only three positive associations between Type I and II TAMs (M41L, L210W, and T215Y with D67N). The strongest significant association between an NRTI and an NNRTI mutation was between L74V and Y181C (*J* = 0.17, *Z* = 8.9, unadjusted *p* < 1 × 10^−11^). Of note, the associations between the five accessory mutations listed above and Type I and II TAMs have also previously recently been described by Svicher and coworkers [6] and Cozzi-Lepri and coworkers in independent datasets [7]. The conditional probabilities and the temporal data columns show that each of the accessory NRTI mutations consistently follows the Type I or II TAMs. Among 12 pairs with a TAM and an accessory mutation, the TAM occurred first more often in all 12 pairs and was preceded by the accessory mutation in only 6% of pairs. In addition to the five accessory mutations in Table 2 (K43E, E44D, V118I, H208Y, and D218E), other NRTI mutations that consistently followed TAMs included the known treatment-selected mutations T69D and T69N. Figure S2 plots the relationship between the log of the ratio of the conditional probability of two mutations versus the log of the ratio in which two mutations develop, indicating that the conditional dependence between mutations is highly correlated with the order in which the mutations develop when they occur together (r^2^ = 0.81, *p* < 0.001).

**Figure 2:**
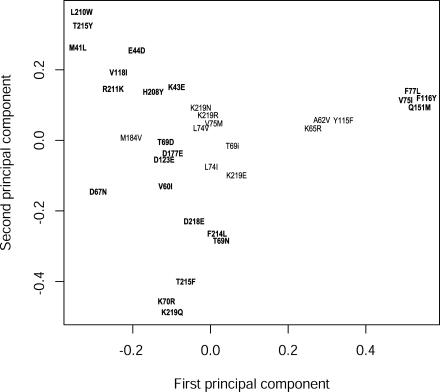
Multidimensional Scaling of 34 HIV-1 Reverse Transcriptase Mutations Includes the 23 mutations obtained from the mutation pairs with highest positive association (Table 2) in bold, and 11 additional clinically relevant nucleoside RT inhibitor resistance mutations (K65R, A62V, T69ins, L74I/V, V75M, Y115F, M184V, and K219R/E/N). The graph is a 2-D projection of the distances among the 37 mutations, in which the distance between any two mutations is measured by their Jaccard dissimilarity coefficient among persons who have received at least one nucleoside RT inhibitor.

The Jaccard dissimilarity coefficients associated with the 561 pairs of 34 mutations were used for a multidimensional scaling. The mutations included in this analysis were the 23 positively associated mutations in Table 2 and 11 additional clinically relevant NRTI-resistance mutations (K65R, A62V, T69ins, L74I/V, V75M, Y115F, M184V, and K219R/E/N). Figure 2 plots the mutations along axes representing the first two principal components. The first principal component accounts for 13% of the total inertia and separates the TAMs from the Q151M-associated mutations, whereas the second principal component accounts for 9% of the total inertia and separates the Type I and Type II TAMs. A62V, K65R, and Y115F are mutations that cluster with Q151M but may also occur with Type II (but not Type I) TAMs. D67N is a Type II TAM that can also occur with Type I TAMs, and it therefore occurs between Type I TAMs and Type II TAMs in terms of the second principal component. The non-TAM mutations, M184V and L74V, demonstrated no clustering with other NRTI-associated mutations.

At several positions, there was sufficient data to contrast covariation patterns for different mutations (Table 2, Figure 2, and Table S3). The Type I TAM, T215Y, clustered with other Type I TAMs, whereas the Type II TAM, T215F, clustered with other Type II TAMs. K219Q/E were Type II TAMs that cluster with other Type II TAMs. In contrast, two less common mutations at this position (K219N/R) were positively associated with Type I TAMs. T69D was associated with both Type I and Type II TAMs, whereas T69N was associated only with Type II TAMs. L74V was associated with the NNRTI-resistance mutations L100I, K103N, and Y181C, whereas L74I was associated with M41L. V75I was associated with Q151M-associated mutations, whereas V75M was positively associated with the Type I TAMs.

Among 19,431 pairs of mutations in sequences from RT inhibitor–naive persons, 41 pairs were significantly associated (family-wise error rate <0.01; Table S4). However, all of the positive associations among RT inhibitor–naive persons were weaker (i.e., had a lower Z score) than the positive associations among treated persons in Table 2.

## Discussion

In this analysis of amino acid covariation in protease and RT sequences from more than 7,000 persons infected with HIV-1 subtype B viruses, we confirmed several previously reported patterns of amino acid covariation and identified many new patterns of covariation. Multidimensional scaling further organized many of the correlations into clusters of co-occurring mutations. RT covariation was dominated by the distinct clustering of the TAMs and Q151M-associated mutations, and by the separation of the Type I and Type II TAMs. Protease covariation was dominated by the clustering of nelfinavir-associated mutations (D30N and N88D), two main groups of PI-resistance mutations associated either with V82A or L90M, and a newly identified cluster of the mutations V32I, L33F, I47V, I50V, I54L/M, and L76V. This new cluster of mutations is associated with decreased susceptibility to all PIs, including the salvage therapy PIs amprenavir and lopinavir and the recently approved PI darunavir. Although none of the sequences in this study were from patients who received darunavir, this drug is highly similar to amprenavir and is affected by the same PI-resistance mutations.

Previous studies of HIV-1 covariation have used either the Pearson correlation for binomial random variables or mutual information [3–6,8–10]. The correlation coefficient is overly sensitive to rare pairs of mutations because its statistical significance is based on a departure from equality between the diagonal and off-diagonal products of a 2 × 2 contingency table. In contrast, mutual information is insensitive to rare pairs of mutations, approaching a high level only for commonly occurring pairs of mutations. We therefore used the Jaccard similarity coefficient, which uses only those sequences in which at least one of a pair of mutations is present, and we assessed the significance of this coefficient using a distribution based on the underlying data.

We also used a conservative correction for multiple comparisons (Holm's method) because our analysis was not designed to identify all covarying mutations but only those with the strongest association. Without a correction for multiple comparisons, 753 pairs of protease mutations from PI-experienced persons and 2,061 pairs of RTI mutations from RTI-experienced persons had a significant Jaccard similarity coefficient at a *p*-value of 0.01 but with the Holm's correction, only 161 pairs of protease mutations and 327 pairs of RTI mutations were significantly associated using a family-wise error rate of 0.01.

Covariation between two mutations may result from the shared inheritance of the mutations from a founder virus, from a shared evolutionary pressure (e.g., an antiretroviral drug) that independently selects for each mutation, or from a functional dependency between the mutations. In our analysis, covariation was unlikely to result from shared inheritance because the most strongly covarying mutations occurred solely among treated HIV-1 isolates, consistent with the repeated selection of the correlated mutations in many different isolates as a result of selective drug pressure rather than the inheritance of the correlated mutations from a small number of ancestral viruses.

However, the possibility that many of the covarying residues resulted from similar selective pressures rather than from functional dependency cannot be excluded. For example, it is possible that some pairs of covarying protease amino acids result from the selective pressure of the same PI or possibly pair of PIs. Shared selective pressure is a possible explanation for why covarying mutations are not necessarily close to one another in tertiary structures (Figure S3) [4]. An analysis of covariation that controls for treatment history would be better able to distinguish functional dependency from shared selective pressure. However, for most PIs and NRTI combinations, insufficient data are available for such an analysis. Identifying similar patterns of covariation in one or more independent lineages (e.g., other non-B subtypes) would also provide additional independent evidence for functional dependency.

Our examination of conditional dependency between mutation pairs, the temporal order in which mutations occur, and the relationship between these two types of data provided new insights into the evolution of protease and RT in persons receiving antiretroviral therapy. A strong positive relationship between the conditional dependency ratio of two mutations and the order in which the mutations occur would represent the most parsimonious mechanism for HIV-1 to develop multiple mutations (i.e., the mutation that occurs more often in a pair of mutations would be on average more likely to occur first). Nonetheless, we found that the positive relationship between conditional dependency and the order of mutation occurrence was stronger for covarying RT (r^2^ = 0.81) compared with protease (r^2^ = 0.56) mutation pairs. This suggests that the number of mutational steps required to develop multiple PI-resistance mutations may be greater on average than that required for developing the same number of multiple NRTI-resistance mutations.

We also found that accessory NRTI-resistance mutations nearly always followed primary NRTI-resistance mutations (particularly the TAMs). In contrast, the commonly recognized accessory PI-resistance mutations were as likely to precede as to follow major PI-resistance mutations. This frequent precedence of accessory PI-resistance mutations results in part from the fact that many of the accessory PI-resistance mutations are polymorphic and therefore present prior to the start of therapy. However, this alone does not explain the marked dependency of some major mutations on polymorphic accessory PI-resistance mutations that occur only at low levels in untreated persons.

The strong positive relationship between conditional probabilities and temporal data that we describe support the validity of previous research, which used cross-sectional data to infer mutational pathways [11] and causality [12,13]. Our results also suggest that there is a complex process underlying the order in which major and accessory PI-resistance mutations develop during PI therapy, and that the designation of major PI-resistance mutations as primary and accessory PI-resistance mutations as secondary often refers only to their roles in causing resistance and not to the order in which they develop.

## Materials and Methods

### Virus sequence data.

Sequences included HIV-1 subtype B RT and protease sequences from published studies in the Stanford HIV Drug Resistance Database (http://hivdb.stanford.edu) [14]. For patients with more than one sequence, only the latest sequence obtained while receiving treatment was analyzed. For each gene, separate analyses were done for the sequences from treatment-experienced and treatment-naive individuals.

RT positions 1–240 and protease positions 1–99 were analyzed. Mutations were defined as differences from the consensus wild-type subtype B amino acid reference sequence (http://hivdb.stanford.edu/pages/asi/releaseNotes/index.html). For each pair of mutations (*X*, *Y*), the numbers of sequences containing both mutations (*X* and *Y*), only one mutation (*X* or *Y*), or neither mutation (not *X*, not *Y*) were counted and used to populate a contingency table. Sequences containing mixtures at either of the two positions were excluded from analysis of that pair of positions.

Antiretroviral treatment–selected mutations were defined based on the results of a previous study, as mutations that were significantly more common in treated than untreated persons after adjusting for multiple comparisons [15]. PI-selected mutations included L10I/V/F/R, V11I, K20R/M/I/T, L23I, L24I, D30N, V32I, L33F/I, E34Q, E35G, M36I/V, K43T, M46I/L/V, G48V/M, I50V/L, F53L, I54V/M/L/T/A/S, K55R, Q58E, L63P, I66F, C67F, A71V/T/I, V72L, G73S/T/C/A, T74A/P/S, L76V, V77I, V82A/T/F/S/L/M, I84V/A/C, I85V, N88D/S/T/G, L89V, L90M, T91S, Q92R/K, I93L, and C95F. Several PI-resistance mutations—particularly those that occur in the substrate cleft or that have a major impact on drug susceptibility—are considered major PI-resistance mutations [2,16]. For the purposes of this study, we defined mutations at positions 24, 30, 32, 46, 47, 48, 50, 53, 54, 76, 82, 84, 88, and 90 as being major PI-resistance mutations. Several PI-resistance mutations—including several that are polymorphic in untreated persons—are commonly considered accessory drug resistance mutations that either compensate for the decreased replication associated with many of the major mutations or that reduce drug susceptibility further when present with a major mutation. Mutations at positions 10, 20, 33, 36, 58, 63, 71, 73, 74, 77, and 93 are usually considered to be accessory mutations. Little attention has been given to the remaining PI-selected mutations, and for the purposes of this paper, we leave them unclassified with respect to the designations major and accessory.

NRTI-selected mutations included T39A, M41L, K43E/Q/N, E44D/A, A62V, K65R, D67N/G/E, T69D/N/S/insertion, K70R, L74V/I, V75I/M/T/A, F77L, V90I, K104N, Y115F, F116Y, V118I, Q151M, M184V/I, E203K, H208Y, L210W, T215Y/F/D/C/E/S/I/V, D218E, K219Q/E/N/R, H221Y, K223Q, and L228H/R. These mutations included the Type I TAMs M41L, L210W, and T215Y, and the Type II TAMs D67N, K70R, T215F, and K219Q/E [7]. Recently described accessory NRTI mutations included T39A, K43E/Q/N, E44D/A, V118I, E203K, H208, D218E, H221Y, K223Q, and L228H/R [3,6,17]. Q151M-associated mutations included A62V, V75I, F77L, F116Y, and Q151M [18,19].

NNRTI-selected mutations included A98G, L100I, K101E/P/N/H, K103N/S, V106A/M, V108I, V179D/E, Y181C/I/V, Y188L/C/H, G190A/S/E/Q, P225H, F227L, M230L, P236L, and K238T.

### Pairwise correlation.

We used the Jaccard similarity coefficient (*J*) to assess covariation among protease and RT mutations. For a given pair of mutations *X* and *Y*, the Jaccard similarity coefficient is calculated as *J* = *N_XY_* /(*N_XY_* + *N_X_*_0_ + *N*_0*Y*_) where *N_XY_* represents the number of sequences containing *X* and *Y*, *N_X0_* represents the number of sequences containing *X* but not *Y*, and *N_0Y_* represents the number of sequences containing *Y* but not *X*. This coefficient represents the probability of both mutations occurring together when either mutation occurs and, therefore, does not inflate the correlation between two mutations that may appear correlated by other measures when both mutations are nearly always absent.

To test whether observed Jaccard similarity coefficients were statistically significant, the expected value of the Jaccard similarity coefficients (*J_RAND_*) and its standard error (*J_SE_*) assuming two mutations (*X* and *Y*) occur independently were calculated for each pair of mutations. *J_RAND_* was calculated as the mean Jaccard similarity coefficient after 2,000 random rearrangements of the *X* or *Y* vector (containing 0 or 1 for presence or absence of a mutation, respectively). *J_SE_* was calculated using a jackknifed procedure, which removed one sequence at a time, repeatedly for each sequence. The standardized score *Z*, *Z* = (*J* − *J_RAND_*) / *J_SE_*, indicates a significant positive association (*Z* > 2.56) or a significant negative association (*Z* < −2.56) at an unadjusted *p* < 0.01.

Holm's method was used to control the family-wise error rate for multiple hypothesis testing [20]. The *p*-values of observed Jaccard similarity coefficients for all pairs of mutations were ranked in descending order. Starting from the smallest *p* (rank *r* = *n*, where *n* is the number of pairs), we compared each *p* of rank *r* with a significance cutoff of 0.01 / *r* as long as *p_r_* ≤ 0.01 / *r*. All *p*-values from *p_r_*…*p_n_* were considered to be statistically significant.

To deal with contingency tables containing 0 for *N_XY_* (potentially leading to Z scores of −∞), we generated a conservative nonzero approximation of *J_SE_* using the following procedure. Given a dataset of *n* sequences, *x* with mutation *X* and *y* with mutation *Y*, we computed the probability of both mutations (*P_XY_*), mutation *X* but not *Y* (*P_X_*_0_), mutation *Y* but not *X* (*P*_0*Y*_), and neither mutation (*P*_00_) under the null hypothesis of independence by *P_XY_* = (*x / n*) × (*y* / *n*), *P_X_*_0_ = (*x* / *n*) × (*y* / *n*) / n, *P*_0*Y*_ = (*n* − *x*) / *n* × (*y* / *n*) and *P*_00_ = 1 − *P_XY_* − *P_X_*_0_ − *P*_0*Y*_. These probabilities were used to create 200 two-by-two contingency tables with cells containing randomly distributed numbers adding up to 20,000 based on the null hypothesis probabilities of independence.

### Multidimensional scaling.

Given the matrix of dissimilarity coefficients (1 − Jaccard similarity coefficient) for a list of mutations (*X_1_*, *X_2_*, ..., *X_n_*), multidimensional scaling was used to construct points in 2-D space such that the Euclidean distances between these points approximate the entries in the dissimilarity matrix [21]. For a given *k*, it computes points *X_1_*, *X_2_*, … , *X_n_* in 2-D space such that S =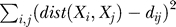 is minimized where dist(*X_i_*, *X_j_*) is the Euclidean distance between *X_i_* and *X_j_* and *d_ij_* is the dissimilarity between *Xi* and *Xj* in the matrix *D*. This was performed using the R function cmdscale (classical multidimensional scaling).

Multidimensional scaling captures the inertia in a dataset in terms of a set of variables (or principal components) that define a projection that encapsulates the maximum amount of inertia in a dataset and is orthogonal (and therefore uncorrelated) to the previous principal component. Using the first and second principal components, we summarized the relationship among mutations in a graphical model, placing pairs of mutations with low Jaccard dissimilarity coefficients close together and mutations with high Jaccard dissimilarity coefficients far apart.

## Supporting Information

Figure S1 Relationship between Conditional Probability and Order of Occurrence within Pairs of Covarying Protease Mutations The relationship between the log of the ratio of the conditional probability of two protease mutations and the log of the ratio in which mutation develops first. A total of 38 protease mutation pairs from Table 1, of which the sum of (*X*,0 → *X*,*Y*) and (0,*Y* → *X*,*Y*) ≥5 , the count of (*X*,0 → *X*,*Y*) or (0,*Y* → *X*,*Y*) is not zero, were plotted.
(22 KB PDF)

Figure S2 Relationship between Conditional Probability and Order of Occurrence within Pairs of Covarying RT Mutations The relationship between the log of the ratio of the conditional probability of two RT mutations and the log of the ratio in which mutation develops first. A total of 31 RT mutation pairs from Table 2, of which the sum of (*X*,0 → *X*,*Y*) and (0,*Y* → *X*,*Y*) ≥5, the count of (*X*,0 → *X*,*Y*) or (0,*Y* → *X*,*Y*) is not zero, were plotted.
(22 KB PDF)

Figure S3 Structural Locations and Distances For Three Sets of Covarying Protease Mutations Locations of residues present in the three most relevant clusters of PI-resistance mutations superimposed on the crystallographic structure of wild-type HIV-1 (1HPV.pdb): L24, M46, I54, and V82 (A); M46, G73, I84, and L90 (B); and V32, L33, I47, I50, I54, and L76 (C). Each panel shows the wild-type residues superimposed on the substrate cleft surface of the protease monomer. The shortest interatomic distances between selected residues are shown. Cluster A usually contains the mutations L24I, M46L > M46I, I54V, and V82A. Cluster B usually contains the mutations M46I, G73S, I84V, and L90M. Cluster C usually contains the mutations V32I, L33F, I47V, I50V, I54L > I54M, and L76V. The cluster consisting of mutations at positions 30 and 88 is not shown, as it is associated with resistance to a single PI (nelfinavir) rather than to multiple PIs.
(2.0 MB TIF)

Table S1 161 Highly Correlated Protease Mutation Pairs from PI-Experienced Persons

Table S2 Highly Correlated HIV-1 Subtype B Protease Mutation Pairs from PI-Naive Persons

Table S3 327 Highly Correlated Protease Mutation Pairs from RTI-Experienced Persons

Table S4 Highly Correlated HIV-1 Subtype B RT Mutation Pairs from RTI-Naive Persons

Text S1 Accession Numbers

### Accession Numbers

The 11,355 GenBank (http://www.ncbi.nlm.nih.gov/Genbank) accession numbers of the sequences used in this study are provided in Text S1.

## Abbreviations

NNRTI: nonnucleoside RT inhibitor
NRTI: nucleoside RT inhibitor
PI: protease inhibitor
RT: reverse transcriptase
TAM: thymidine analog mutation
