# Supplementary material for: HIV-1 Subtype B Protease and Reverse Transcriptase Amino Acid Covariation
Source: PLoS Comput Biol. 2007 May 11;3(5):e87. doi: 10.1371/journal.pcbi.0030087 (PMC1866358; doi:10.1371/journal.pcbi.0030087)
Supplement: Supplementary file 3 [file pcbi.0030087.sd001.doc]

**Text S1. Accession Numbers**

11355 genbank isolates were used in the analysis.

AB020912, AB020913, AB020914, AB020915, AB020916, AB020917, AB020918, AB020919, AB020920, AB020921, AB020923, AB020925, AB078688, AB078694, AB078703, AB078703, AB078704, AB078704, AB078706, AB078708, AB078708, AB078709, AB078709, AB078710, AB078710, AB078711, AB078711, AB078713, AB078714, AB078715, AB078715, AB097865, AB097865, AB097869, AB097869, AB097870, AB097870, AB097873, AB097873, AF004394, AF004394, AF005495, AF005495, AF009369, AF009369, AF009370, AF009370, AF009371, AF009371, AF009372, AF009372, AF009374, AF009374, AF009375, AF009375, AF009377, AF009377, AF009379, AF009379, AF009380, AF009380, AF009381, AF009381, AF011755, AF013420, AF013421, AF013422, AF013423, AF013424, AF013425, AF013426, AF013427, AF013428, AF013429, AF013430, AF013431, AF013432, AF013433, AF013434, AF013435, AF013436, AF013437, AF013438, AF013439, AF013440, AF013441, AF013442, AF013443, AF013444, AF013445, AF013447, AF013449, AF013451, AF013453, AF013455, AF013457, AF013459, AF013461, AF013463, AF013841, AF013842, AF013843, AF013844, AF013845, AF013846, AF013847, AF013848, AF013849, AF013850, AF013851, AF013852, AF013853, AF013855, AF013856, AF013857, AF013859, AF013860, AF013861, AF013862, AF013863, AF013865, AF013866, AF013869, AF013870, AF013871, AF013872, AF013873, AF013874, AF013875, AF013876, AF013877, AF013879, AF013881, AF013882, AF013883, AF013884, AF013885, AF013886, AF013887, AF013888, AF013889, AF013890, AF013893, AF013894, AF013895, AF013896, AF013899, AF013901, AF013902, AF013903, AF013905, AF013906, AF013907, AF013908, AF021273, AF021291, AF021292, AF021293, AF021294, AF021295, AF021296, AF021297, AF021298, AF021300, AF021301, AF021302, AF021303, AF021304, AF021305, AF021306, AF021307, AF021308, AF021309, AF021310, AF021311, AF021312, AF021313, AF021314, AF021315, AF021316, AF021317, AF025722, AF025724, AF025726, AF025728, AF025731, AF025732, AF025734, AF025735, AF025736, AF025744, AF042100, AF042100, AF042101, AF042101, AF042103, AF042103, AF042104, AF042104, AF042105, AF042105, AF047281, AF047291, AF047294, AF047299, AF047301, AF047306, AF047311, AF047317, AF047320, AF047321, AF056637, AF056643, AF056644, AF056647, AF056651, AF056652, AF056656, AF056660, AF056662, AF056663, AF056665, AF056667, AF056668, AF056669, AF056671, AF056674, AF056676, AF056679, AF056682, AF056684, AF056686, AF076440, AF076443, AF076444, AF076445, AF076446, AF076447, AF078566, AF078567, AF078569, AF078571, AF078572, AF078573, AF078579, AF078581, AF078583, AF078584, AF078588, AF078597, AF078600, AF078601, AF078602, AF078603, AF078604, AF078605, AF078606, AF079981, AF079982, AF079983, AF079984, AF079990, AF079991, AF079992, AF079993, AF079994, AF085092, AF085096, AF085128, AF085129, AF085131, AF088084, AF088088, AF088120, AF088121, AF088123, AF090467, AF090468, AF090469, AF090471, AF090474, AF090476, AF090479, AF090480, AF090481, AF090482, AF090483, AF090484, AF090486, AF090487, AF090490, AF090491, AF090494, AF090496, AF090497, AF090498, AF090499, AF090500, AF090501, AF090502, AF090503, AF090504, AF090505, AF090506, AF090508, AF090509, AF090510, AF090512, AF090515, AF090516, AF090517, AF090518, AF090520, AF090521, AF090522, AF090524, AF090526, AF090529, AF090530, AF090531, AF090532, AF096881, AF096881, AF096882, AF096882, AF096883, AF096883, AF096884, AF096884, AF096891, AF096892, AF096893, AF096893, AF096894, AF096894, AF102279, AF102280, AF102284, AF102285, AF102287, AF102288, AF102292, AF102293, AF102295, AF102296, AF102301, AF102302, AF102306, AF102308, AF102313, AF102314, AF102318, AF102322, AF102327, AF102328, AF102330, AF102333, AF102335, AF102338, AF102342, AF102344, AF102349, AF102352, AF102357, AF102360, AF102362, AF102365, AF102368, AF117501, AF136789, AF136791, AF136792, AF136793, AF136798, AF136799, AF136800, AF136801, AF136803, AF136804, AF142174, AF142185, AF142205, AF142239, AF153370, AF153372, AF153374, AF153376, AF153378, AF153380, AF153382, AF153384, AF153386, AF153388, AF153390, AF153392, AF153394, AF153396, AF153398, AF153400, AF153402, AF153404, AF153406, AF153408, AF154954, AF154955, AF154956, AF154957, AF154958, AF154959, AF154960, AF154961, AF154962, AF154963, AF158749, AF158751, AF158753, AF158755, AF158757, AF158759, AF158761, AF158763, AF158765, AF158767, AF158769, AF158771, AF158773, AF158775, AF158777, AF158779, AF158781, AF158783, AF158785, AF158789, AF158791, AF158793, AF158795, AF158797, AF158799, AF158801, AF158803, AF158805, AF158807, AF158809, AF158811, AF158813, AF158815, AF165586, AF165587, AF165588, AF165589, AF165590, AF165591, AF165592, AF165593, AF165594, AF168208, AF168234, AF169043, AF169045, AF169047, AF169049, AF169051, AF169055, AF169057, AF169059, AF169061, AF169063, AF169065, AF169067, AF169069, AF169071, AF169073, AF169077, AF169081, AF169083, AF169085, AF169087, AF169089, AF169091, AF169093, AF169097, AF169099, AF169101, AF169103, AF169105, AF169107, AF169109, AF169111, AF169113, AF169115, AF169117, AF169119, AF186766, AF186767, AF186768, AF186769, AF186770, AF186771, AF198038, AF198038, AF198039, AF198039, AF198041, AF198042, AF198042, AF198043, AF198043, AF198044, AF198044, AF198045, AF198045, AF198046, AF198046, AF198047, AF198047, AF198048, AF198048, AF198049, AF198049, AF198050, AF198050, AF198448, AF198448, AF199241, AF199243, AF199245, AF199247, AF199249, AF199251, AF199252, AF199254, AF199256, AF199258, AF199260, AF199262, AF199265, AF199269, AF199270, AF199272, AF199273, AF199309, AF199319, AF199321, AF204295, AF204296, AF204297, AF204298, AF204299, AF204300, AF204301, AF204302, AF204303, AF204304, AF204305, AF204306, AF204307, AF204308, AF204309, AF204310, AF204311, AF204312, AF204313, AF204314, AF204315, AF204316, AF204317, AF204318, AF204319, AF204320, AF204321, AF204322, AF204323, AF204324, AF204325, AF204326, AF204327, AF204328, AF204329, AF204330, AF204331, AF204332, AF204333, AF204334, AF204335, AF204336, AF204337, AF204338, AF204339, AF204340, AF204341, AF204342, AF204343, AF204344, AF204345, AF204346, AF204347, AF204348, AF204349, AF204350, AF204351, AF204352, AF204353, AF204354, AF204355, AF204356, AF204357, AF204358, AF204359, AF204360, AF204361, AF204362, AF204363, AF204364, AF204365, AF204366, AF204367, AF204368, AF204369, AF204370, AF204371, AF204372, AF204373, AF204374, AF204375, AF204376, AF204377, AF204378, AF204379, AF204380, AF204381, AF204382, AF204383, AF204384, AF204385, AF204386, AF204387, AF204388, AF204389, AF204390, AF204391, AF204392, AF204393, AF204394, AF221016, AF221017, AF221018, AF221019, AF221020, AF221021, AF221022, AF221023, AF221024, AF221026, AF221027, AF221028, AF221029, AF221030, AF221031, AF221032, AF221033, AF221034, AF226078, AF226079, AF226080, AF226081, AF226082, AF226083, AF226084, AF226085, AF226086, AF226087, AF226088, AF226089, AF226090, AF226091, AF226092, AF226093, AF226094, AF226095, AF226096, AF226097, AF226098, AF226099, AF226100, AF226101, AF226102, AF226103, AF226104, AF226105, AF226106, AF226107, AF226108, AF226109, AF226110, AF226111, AF226112, AF226113, AF226114, AF226115, AF226116, AF226117, AF226118, AF226119, AF226120, AF226121, AF226122, AF226123, AF226124, AF226125, AF226126, AF226127, AF226128, AF226129, AF226130, AF226131, AF226132, AF226133, AF226134, AF226135, AF226136, AF226137, AF226138, AF226139, AF226140, AF226142, AF226143, AF226144, AF226145, AF226146, AF226147, AF226148, AF226149, AF227688, AF227690, AF227692, AF227694, AF227696, AF227698, AF227700, AF227702, AF227704, AF227706, AF227708, AF227710, AF227712, AF227714, AF227716, AF227718, AF227720, AF227722, AF227724, AF227726, AF239202, AF239204, AF239205, AF239211, AF247031, AF247033, AF247038, AF247855, AF247856, AF247857, AF247858, AF247859, AF247860, AF247861, AF247862, AF247863, AF247865, AF247866, AF247867, AF247868, AF247869, AF247870, AF247871, AF247872, AF247873, AF247875, AF247876, AF247881, AF247883, AF247887, AF247892, AF247896, AF247900, AF247903, AF251942, AF251942, AF251943, AF251943, AF251944, AF251944, AF251946, AF251946, AF251947, AF251947, AF251948, AF251948, AF251950, AF251950, AF251951, AF251951, AF251952, AF251952, AF251953, AF251954, AF251954, AF251955, AF251955, AF251956, AF251956, AF251957, AF251957, AF251958, AF251959, AF251960, AF251960, AF251961, AF251961, AF251962, AF251962, AF251963, AF251964, AF251964, AF251965, AF251965, AF251966, AF251967, AF251967, AF251968, AF251968, AF251969, AF251969, AF251970, AF251970, AF251971, AF251971, AF251972, AF251973, AF251973, AF251974, AF251974, AF251975, AF251975, AF251976, AF251977, AF251977, AF251978, AF251978, AF251979, AF251979, AF251980, AF251980, AF251981, AF251981, AF251982, AF251982, AF251983, AF251983, AF251984, AF251984, AF251985, AF251985, AF251986, AF251987, AF251987, AF251988, AF251988, AF251989, AF251989, AF251997, AF251997, AF251998, AF251998, AF251999, AF251999, AF252000, AF252000, AF252001, AF252001, AF252002, AF252002, AF252003, AF252004, AF252004, AF252005, AF252005, AF252006, AF252006, AF252007, AF252007, AF252008, AF252008, AF252009, AF252009, AF252010, AF252010, AF252011, AF252011, AF252012, AF252012, AF252013, AF252013, AF252014, AF252014, AF252015, AF252015, AF252016, AF252017, AF252017, AF252018, AF252018, AF252019, AF252019, AF252020, AF252020, AF252021, AF252021, AF252022, AF252023, AF252023, AF252024, AF252024, AF252025, AF252025, AF252026, AF252026, AF252027, AF252027, AF252028, AF252028, AF252029, AF252029, AF252030, AF252030, AF252031, AF252031, AF252032, AF252032, AF252033, AF252033, AF252034, AF252034, AF252035, AF252035, AF252036, AF252036, AF252037, AF252037, AF252038, AF252038, AF252039, AF252040, AF252040, AF252041, AF252041, AF252042, AF252042, AF252043, AF252043, AF252044, AF252044, AF252046, AF252046, AF252047, AF252048, AF252048, AF252049, AF252049, AF252050, AF252050, AF252051, AF252051, AF252052, AF252052, AF252053, AF252053, AF252054, AF252054, AF252055, AF252055, AF252056, AF252056, AF252057, AF252057, AF252058, AF252058, AF252059, AF252059, AF252060, AF252060, AF252061, AF252061, AF252062, AF252062, AF252063, AF252063, AF252064, AF252064, AF252065, AF252065, AF252066, AF252067, AF252067, AF252068, AF252068, AF252069, AF252069, AF252070, AF252070, AF252071, AF252072, AF252072, AF252073, AF252073, AF252074, AF252074, AF252075, AF252075, AF252076, AF252077, AF252077, AF252078, AF252078, AF252079, AF252079, AF252080, AF252080, AF252081, AF252081, AF252082, AF252083, AF252084, AF252084, AF252085, AF252085, AF252086, AF252086, AF252087, AF252087, AF252088, AF252088, AF252089, AF252089, AF252090, AF252090, AF252091, AF252091, AF259905, AF259907, AF259911, AF259913, AF259915, AF259917, AF259919, AF259921, AF259923, AF259925, AF259927, AF259929, AF259932, AF259934, AF259936, AF259938, AF259939, AF259942, AF259943, AF259945, AF259946, AF259948, AF259950, AF266532, AF266533, AF266534, AF266535, AF266537, AF266538, AF266539, AF273145, AF273152, AF273162, AF273168, AF273169, AF273178, AF273193, AF273197, AF273199, AF273200, AF273201, AF273203, AF273207, AF273210, AF281872, AF282132, AF282134, AF282143, AF282147, AF282149, AF282150, AF282151, AF282154, AF282163, AF286230, AF288791, AF288792, AF288795, AF288797, AF288799, AF288801, AF288803, AF288806, AF288807, AF288808, AF294518, AF294518, AF294521, AF294521, AF294524, AF294524, AF294527, AF294527, AF294531, AF294531, AF294533, AF294533, AF294536, AF294536, AF294540, AF294540, AF294545, AF294545, AF294549, AF294549, AF294551, AF294551, AF294554, AF294554, AF294558, AF294558, AF294559, AF294559, AF294561, AF294561, AF294564, AF294564, AF295298, AF308493, AF308494, AF308501, AF308502, AF308512, AF308513, AF308514, AF311157, AF311159, AF311162, AF311177, AF311179, AF311187, AF315232, AF315233, AF315234, AF315235, AF315236, AF315237, AF315238, AF315239, AF315240, AF315241, AF315242, AF315243, AF315244, AF315245, AF315246, AF315247, AF315248, AF315249, AF315250, AF315251, AF315252, AF315253, AF315254, AF315255, AF315256, AF315258, AF315259, AF315260, AF315261, AF315262, AF315263, AF315264, AF315265, AF315266, AF315267, AF315268, AF315269, AF315271, AF315272, AF315273, AF315274, AF315741, AF315742, AF315743, AF315744, AF315745, AF315746, AF315747, AF315748, AF315749, AF315751, AF315755, AF315756, AF315757, AF315758, AF315759, AF315760, AF315761, AF315762, AF315763, AF315764, AF315765, AF315766, AF315767, AF315770, AF315771, AF315774, AF315775, AF315776, AF315777, AF315778, AF315779, AF315781, AF315782, AF315783, AF316837, AF316837, AF316844, AF316844, AF320534, AF320536, AF320537, AF320538, AF320539, AF320540, AF320541, AF320542, AF320543, AF320544, AF320545, AF320546, AF320547, AF320548, AF320549, AF320550, AF320551, AF320552, AF320553, AF320554, AF320555, AF320556, AF320557, AF320558, AF320559, AF326197, AF328562, AF328564, AF328566, AF328567, AF328568, AF328569, AF328571, AF328574, AF328576, AF328577, AF328578, AF328580, AF328581, AF328582, AF328583, AF328584, AF328585, AF328588, AF328590, AF328592, AF328595, AF328596, AF328597, AF328598, AF328599, AF328600, AF328601, AF328602, AF328603, AF328604, AF328605, AF328606, AF328607, AF328608, AF328609, AF328610, AF328611, AF328612, AF328613, AF328614, AF328615, AF328616, AF328617, AF328618, AF328619, AF328620, AF328621, AF328622, AF328623, AF328624, AF328625, AF328626, AF329582, AF329583, AF329584, AF329585, AF329586, AF329587, AF329588, AF329589, AF329590, AF329591, AF329592, AF329593, AF329594, AF329595, AF329596, AF329597, AF329598, AF329599, AF329600, AF329601, AF329602, AF329603, AF329604, AF329605, AF329606, AF329607, AF329608, AF329609, AF329610, AF329611, AF329612, AF329613, AF329614, AF329615, AF329616, AF329617, AF329618, AF329619, AF329620, AF329621, AF329622, AF329623, AF329624, AF329625, AF329626, AF329627, AF329628, AF329629, AF329630, AF329631, AF349317, AF349318, AF349318, AF349320, AF349320, AF349322, AF349323, AF349323, AF349324, AF349325, AF349325, AF349326, AF349327, AF349327, AF349328, AF349328, AF349329, AF349330, AF349330, AF349331, AF349331, AF349332, AF349334, AF349334, AF349335, AF349338, AF349339, AF349339, AF349342, AF349343, AF349343, AF349344, AF349345, AF349345, AF349346, AF349346, AF349349, AF349350, AF349350, AF349351, AF349351, AF349352, AF349352, AF349354, AF349354, AF349355, AF349358, AF349358, AF349359, AF349362, AF349362, AF349365, AF349365, AF349368, AF349369, AF349370, AF349372, AF349373, AF349373, AF349376, AF349378, AF349380, AF349380, AF349385, AF349385, AF349386, AF349386, AF349388, AF349388, AF349391, AF349391, AF349392, AF349392, AF349399, AF349399, AF349402, AF349402, AF349403, AF349403, AF357606, AF357606, AF357607, AF357607, AF357608, AF357608, AF357609, AF357609, AF357610, AF357610, AF357611, AF357611, AF357612, AF357612, AF357613, AF357613, AF357614, AF357614, AF357615, AF357615, AF357616, AF357617, AF357617, AF357618, AF357618, AF357619, AF357619, AF357620, AF357620, AF357621, AF357622, AF357622, AF357623, AF357623, AF357624, AF357624, AF357625, AF357626, AF357626, AF357627, AF357627, AF357628, AF357628, AF357629, AF357630, AF357632, AF357632, AF357633, AF357633, AF357634, AF357634, AF357635, AF357635, AF357636, AF357637, AF357637, AF357638, AF357638, AF357639, AF357639, AF357640, AF357641, AF357642, AF357643, AF357644, AF357645, AF357645, AF357646, AF357646, AF357647, AF357648, AF357648, AF357649, AF357649, AF357650, AF357650, AF357651, AF357651, AF357652, AF357652, AF357653, AF357654, AF357655, AF357656, AF357657, AF357658, AF357659, AF357661, AF357662, AF357662, AF357663, AF357664, AF357664, AF357665, AF357666, AF357667, AF357668, AF357669, AF357670, AF357671, AF357672, AF357673, AF357673, AF357674, AF357675, AF357675, AF357676, AF357676, AF357677, AF357677, AF357678, AF357679, AF357679, AF357680, AF357680, AF357681, AF357681, AF357682, AF357683, AF357683, AF357684, AF357684, AF357685, AF357686, AF357686, AF357687, AF357687, AF357688, AF357688, AF357690, AF357691, AF357692, AF357693, AF357693, AF357694, AF357694, AF357695, AF357695, AF357696, AF357696, AF357697, AF357698, AF357699, AF357700, AF357701, AF357701, AF357702, AF357702, AF357703, AF357703, AF357704, AF357705, AF357706, AF357706, AF357707, AF357708, AF357709, AF357710, AF357711, AF357711, AF357712, AF357712, AF357713, AF357713, AF357714, AF357714, AF357715, AF357715, AF357716, AF357716, AF357717, AF357717, AF357718, AF357718, AF357719, AF357719, AF357720, AF357721, AF357721, AF357722, AF357722, AF357723, AF357723, AF357724, AF357725, AF357725, AF357726, AF357726, AF357727, AF357728, AF357729, AF357730, AF357730, AF357731, AF357731, AF357732, AF357733, AF357733, AF357734, AF357734, AF357735, AF357735, AF357736, AF357736, AF357737, AF357738, AF357739, AF357740, AF357740, AF357747, AF357747, AF357750, AF357750, AF357754, AF357754, AF357755, AF357755, AF357756, AF357756, AF357757, AF357757, AF357760, AF357760, AF357763, AF357763, AF357764, AF357764, AF357765, AF357765, AF357770, AF357770, AF357774, AF357774, AF357776, AF357776, AF357777, AF357777, AF357779, AF357779, AF357780, AF357780, AF357781, AF357781, AF357783, AF357783, AF357784, AF357784, AF357788, AF357788, AF357791, AF357791, AF357792, AF357792, AF357793, AF357793, AF357794, AF357794, AF357796, AF357796, AF357797, AF357797, AF357798, AF357798, AF357799, AF357799, AF357800, AF357800, AF357801, AF357801, AF357802, AF357802, AF357803, AF357803, AF357804, AF357804, AF357805, AF357805, AF357806, AF357806, AF357807, AF357807, AF357808, AF357808, AF357809, AF357809, AF357810, AF357810, AF357811, AF357811, AF357812, AF357812, AF357813, AF357813, AF357814, AF357814, AF357815, AF357815, AF357816, AF357816, AF357817, AF357817, AF357818, AF357818, AF357819, AF357819, AF357820, AF357820, AF357821, AF357821, AF357822, AF357822, AF357823, AF357823, AF357824, AF357824, AF357825, AF357825, AF357826, AF357826, AF357827, AF357827, AF357828, AF357828, AF357829, AF357829, AF357830, AF357830, AF357831, AF357831, AF357832, AF357832, AF357833, AF357833, AF366277, AF366278, AF366280, AF366285, AF366286, AF366287, AF366291, AF369796, AF369796, AF369797, AF369797, AF369798, AF369798, AF369799, AF369799, AF369800, AF369800, AF369801, AF369801, AF369802, AF369802, AF369803, AF369803, AF369804, AF369804, AF369805, AF369805, AF369806, AF369806, AF369807, AF369807, AF369808, AF369808, AF369809, AF369809, AF369810, AF369810, AF369811, AF369811, AF369812, AF369812, AF375229, AF375231, AF375233, AF375235, AF375237, AF375239, AF375241, AF376522, AF376522, AF376523, AF376523, AF376524, AF376524, AF376525, AF376525, AF376526, AF376526, AF376527, AF376527, AF376528, AF376528, AF376529, AF376529, AF376531, AF376531, AF376532, AF376532, AF376533, AF376533, AF376534, AF376534, AF376535, AF376535, AF376536, AF376536, AF376537, AF376537, AF376538, AF376538, AF376539, AF376539, AF376540, AF376540, AF376541, AF376541, AF376542, AF376542, AF376543, AF376543, AF376544, AF376544, AF376545, AF376545, AF376546, AF376546, AF376547, AF376547, AF376548, AF376548, AF376549, AF376549, AF376550, AF376550, AF376551, AF376551, AF376552, AF376552, AF376553, AF376553, AF376554, AF376554, AF376555, AF376556, AF376556, AF376557, AF376557, AF376558, AF376558, AF376559, AF376559, AF376560, AF376560, AF376561, AF376561, AF376562, AF376562, AF376563, AF376563, AF376564, AF376564, AF376565, AF376565, AF376566, AF376566, AF376567, AF376567, AF376568, AF376568, AF376569, AF376569, AF376570, AF376570, AF376571, AF376571, AF376572, AF376572, AF376573, AF376574, AF376574, AF376575, AF376575, AF376576, AF376576, AF376577, AF376577, AF376578, AF376579, AF376579, AF376580, AF376580, AF376581, AF376581, AF376582, AF376582, AF376583, AF376583, AF376584, AF376584, AF376585, AF376585, AF376586, AF376586, AF376587, AF376587, AF376588, AF376588, AF376589, AF376589, AF376590, AF376590, AF376591, AF376591, AF376592, AF376592, AF376593, AF376593, AF376594, AF376594, AF376595, AF376595, AF376596, AF376596, AF376597, AF376597, AF376598, AF376598, AF376599, AF376599, AF376600, AF376600, AF376601, AF376601, AF376602, AF376602, AF376603, AF376603, AF376604, AF376604, AF376605, AF376605, AF376606, AF376606, AF376607, AF376607, AF376608, AF376608, AF376609, AF376609, AF376611, AF376612, AF376612, AF376613, AF376613, AF376614, AF376614, AF376615, AF376615, AF376616, AF376616, AF376617, AF376617, AF376618, AF376618, AF376619, AF376619, AF376620, AF376620, AF376621, AF376621, AF376622, AF376622, AF376623, AF376623, AF376624, AF376624, AF376625, AF376625, AF376626, AF376626, AF376627, AF376627, AF376628, AF376628, AF376629, AF376629, AF376630, AF376630, AF376631, AF376631, AF376632, AF376632, AF376633, AF376633, AF376634, AF376634, AF376635, AF376635, AF376636, AF376636, AF376637, AF376637, AF376638, AF376638, AF376639, AF376639, AF376640, AF376640, AF376641, AF376641, AF376642, AF376642, AF376643, AF376643, AF376644, AF376644, AF376645, AF376645, AF376646, AF376646, AF376647, AF376647, AF376648, AF376648, AF376649, AF376649, AF376650, AF376650, AF376651, AF376651, AF376652, AF376652, AF376653, AF376653, AF376654, AF376654, AF376655, AF376655, AF376656, AF376656, AF376657, AF376657, AF376658, AF376658, AF376659, AF376659, AF376660, AF376660, AF376661, AF376661, AF376662, AF376662, AF376663, AF376663, AF376664, AF376664, AF376665, AF376665, AF376666, AF376666, AF376667, AF376667, AF376668, AF376668, AF376669, AF376669, AF376670, AF376670, AF376671, AF376671, AF376672, AF376672, AF376673, AF376673, AF376674, AF376674, AF376675, AF376675, AF376676, AF376676, AF376677, AF376677, AF376678, AF376678, AF376679, AF376679, AF376680, AF376680, AF376681, AF376681, AF376682, AF376682, AF376683, AF376683, AF376684, AF376684, AF376685, AF376685, AF376686, AF376686, AF376687, AF376687, AF376688, AF376688, AF376689, AF376689, AF376690, AF376690, AF376691, AF376691, AF376692, AF376692, AF376693, AF376693, AF376694, AF376694, AF376695, AF376695, AF376696, AF376696, AF376697, AF376697, AF376698, AF376698, AF376699, AF376699, AF376700, AF376700, AF376701, AF376701, AF376702, AF376702, AF376703, AF376703, AF376704, AF376704, AF376705, AF376705, AF376706, AF376706, AF376707, AF376707, AF376708, AF376708, AF376709, AF376709, AF376710, AF376710, AF376711, AF376711, AF376712, AF376712, AF376713, AF376713, AF376714, AF376714, AF376715, AF376715, AF376716, AF376716, AF376717, AF376717, AF376718, AF376718, AF376719, AF376719, AF376720, AF376720, AF376722, AF376722, AF376723, AF376723, AF376724, AF376724, AF388123, AF388128, AF407367, AF407368, AF407372, AF407373, AF407374, AF411964, AF413811, AF413812, AF413813, AF413814, AF413816, AF413817, AF413818, AF413819, AF413820, AF413821, AF413822, AF413823, AF413824, AF413825, AF413826, AF413827, AF413828, AF413829, AF413830, AF413831, AF413833, AF413834, AF413836, AF413837, AF413838, AF413839, AF413840, AF413841, AF413842, AF413843, AF413844, AF413845, AF413846, AF413847, AF413848, AF413849, AF413850, AF413851, AF413852, AF413853, AF413854, AF413855, AF413856, AF413857, AF413859, AF413860, AF413861, AF413862, AF413863, AF413864, AF413865, AF413866, AF413867, AF413868, AF413869, AF413870, AF413871, AF413872, AF413873, AF413874, AF413875, AF413876, AF413877, AF413878, AF413879, AF413880, AF413881, AF413882, AF413883, AF413884, AF413885, AF413886, AF413887, AF413888, AF413889, AF413890, AF413891, AF413892, AF413893, AF413894, AF413895, AF413896, AF413897, AF413898, AF413899, AF413900, AF413902, AF413903, AF413904, AF413905, AF413907, AF413908, AF413909, AF413910, AF413911, AF413912, AF418656, AF422723, AF422724, AF422725, AF422726, AF422727, AF422728, AF422729, AF422730, AF422731, AF422732, AF422733, AF422734, AF422735, AF422736, AF422737, AF422738, AF422739, AF422740, AF422741, AF422742, AF422743, AF422744, AF422745, AF422746, AF422747, AF425383, AF425383, AF425384, AF425384, AF425386, AF425386, AF425387, AF425387, AF425388, AF425392, AF425392, AF425393, AF425393, AF425394, AF425398, AF425399, AF425399, AF425400, AF425402, AF425402, AF425403, AF425403, AF425405, AF425405, AF425406, AF425406, AF425407, AF425407, AF425408, AF425408, AF425409, AF425409, AF425413, AF425413, AF425416, AF425416, AF425417, AF425417, AF425418, AF425418, AF425419, AF425419, AF425422, AF425422, AF425423, AF425423, AF425424, AF425424, AF425425, AF425425, AF425428, AF425428, AF425429, AF425429, AF425430, AF425430, AF425431, AF425431, AF425432, AF425433, AF425433, AF425434, AF425434, AF425435, AF425436, AF425439, AF425439, AF425440, AF425440, AF425444, AF425444, AF425447, AF425448, AF425458, AF425458, AF425460, AF425460, AF425463, AF425464, AF425464, AF425465, AF425465, AF425467, AF425467, AF425470, AF425470, AF425471, AF425471, AF425472, AF425472, AF425473, AF425473, AF425474, AF425474, AF425477, AF425477, AF425478, AF425478, AF425479, AF425479, AF425480, AF425480, AF425481, AF425482, AF425482, AF425483, AF425483, AF425484, AF442161, AF442162, AF442165, AF442166, AF442167, AF442168, AF442169, AF442170, AF442171, AF442173, AF442174, AF442177, AF442181, AF442182, AF442183, AF442184, AF442185, AF442186, AF442187, AF442188, AF444171, AF444173, AF444174, AF444176, AF444178, AF444181, AF444182, AF444185, AF444186, AF444188, AF444190, AF444192, AF447821, AF447821, AF447829, AF447829, AF447830, AF447830, AF447834, AF447834, AF447837, AF447837, AF447847, AF447847, AF447848, AF447848, AF447849, AF447849, AF447854, AF447854, AF447855, AF447855, AF467726, AF480743, AF480744, AF480745, AF480746, AF480747, AF480748, AF480749, AF480750, AF480751, AF480752, AF480753, AF480754, AF480755, AF480756, AF480757, AF480758, AF480759, AF480760, AF480761, AF480762, AF480763, AF480764, AF480765, AF480766, AF480767, AF480768, AF480769, AF480770, AF480771, AF480772, AF480773, AF480774, AF480775, AF480776, AF480777, AF480778, AF480779, AF480780, AF480781, AF480782, AF480783, AF480784, AF480785, AF480786, AF480787, AF480788, AF480789, AF480790, AF480791, AF480792, AF480793, AF480794, AF480795, AF480796, AF480797, AF480798, AF480799, AF480800, AF480801, AF480803, AF480804, AF480805, AF480806, AF480807, AF480808, AF480810, AF480811, AF480812, AF480813, AF480814, AF480815, AF480816, AF480817, AF480818, AF480819, AF480820, AF480824, AF480827, AF480830, AF487122, AF487125, AF487125, AF487126, AF487128, AF487131, AF487131, AF487132, AF487133, AF487135, AF487135, AF487136, AF487138, AF487139, AF487139, AF493346, AF493346, AF493354, AF493354, AF493377, AF493377, AF493378, AF493378, AF493379, AF493379, AF493380, AF493380, AF493381, AF493381, AF493382, AF493382, AF493383, AF493383, AF493384, AF493384, AF493385, AF493385, AF493386, AF493386, AF493387, AF493387, AF493388, AF493388, AF493389, AF493389, AF493390, AF493390, AF493391, AF493391, AF493392, AF493392, AF493393, AF493393, AF493394, AF493394, AF493395, AF493395, AF493396, AF493396, AF493397, AF493397, AF493398, AF493398, AF493399, AF493399, AF493400, AF493400, AF493401, AF493401, AF493402, AF493402, AF493403, AF493403, AF493404, AF493404, AF493405, AF493405, AF493406, AF493406, AF493407, AF493407, AF493408, AF493408, AF493409, AF493409, AF493410, AF493410, AF493411, AF493411, AF493412, AF493412, AF493413, AF493413, AF493414, AF493414, AF493415, AF493415, AF504553, AF504556, AF504561, AF504562, AF504564, AF504565, AF504570, AF504571, AF504572, AF504573, AF504575, AF504576, AF504579, AF504582, AF504583, AF504584, AF504587, AF504588, AF504589, AF504591, AF504592, AF504593, AF504597, AF504598, AF504599, AF504600, AF504602, AF504604, AF504605, AF504607, AF504608, AF504609, AF504610, AF504611, AF504612, AF504613, AF504615, AF504616, AF504617, AF504618, AF504619, AF504622, AF504625, AF504627, AF504628, AF504629, AF504630, AF504631, AF504633, AF504635, AF504637, AF504640, AF504642, AF504643, AF504644, AF508281, AF508282, AF508284, AF508287, AF513992, AF513994, AF513995, AF513998, AF514000, AF514001, AF514002, AF514004, AF514005, AF514008, AF514009, AF514010, AF514013, AF514015, AF514019, AF514027, AF514030, AF514033, AF514034, AF514035, AF514040, AF514042, AF514043, AF514044, AF514051, AF514052, AF514054, AF514056, AF514058, AF514059, AF514060, AF514061, AF514063, AF514065, AF514069, AF514070, AF514072, AF514075, AF514076, AF514077, AF514078, AF514079, AF514080, AF514081, AF514082, AF514088, AF514090, AF514091, AF514097, AF514101, AF514104, AF514106, AF514108, AF514109, AF514110, AF514112, AF514113, AF514116, AF514120, AF514125, AF514128, AF514130, AF514131, AF514133, AF514134, AF514135, AF514138, AF514140, AF514142, AF514143, AF514144, AF514145, AF514146, AF514147, AF514151, AF514152, AF514154, AF514155, AF514156, AF514157, AF514161, AF514166, AF514167, AF514169, AF514171, AF514173, AF514174, AF514175, AF514177, AF514179, AF514186, AF514189, AF514190, AF514191, AF514192, AF514196, AF514199, AF514200, AF514201, AF514205, AF514207, AF514208, AF514209, AF514210, AF514211, AF514213, AF514215, AF514216, AF514220, AF514223, AF514224, AF514225, AF514227, AF514228, AF514230, AF514231, AF514233, AF514236, AF514238, AF514239, AF514240, AF514241, AF514242, AF514243, AF514244, AF514246, AF514247, AF514249, AF514250, AF514252, AF514253, AF514254, AF514255, AF514257, AF514259, AF514260, AF514261, AF514267, AF514269, AF514270, AF514272, AF514273, AF514274, AF514275, AF514276, AF514277, AF517018, AF517019, AF517020, AF517021, AF517022, AF517023, AF517024, AF517026, AF517027, AF517028, AF517030, AF517031, AF517032, AF517034, AF517035, AF517036, AF517037, AF517038, AF517039, AF517040, AF517042, AF517043, AF517044, AF517046, AF517047, AF517048, AF517050, AF517051, AF517052, AF517054, AF517055, AF517056, AF517058, AF517059, AF517060, AF517061, AF517062, AF517063, AF517066, AF517067, AF517068, AF517255, AF517257, AF517258, AF517259, AF517259, AF517261, AF517262, AF517262, AF517263, AF517264, AF517264, AF517265, AF517265, AF517266, AF517266, AF517267, AF517267, AF517268, AF517268, AF517269, AF517269, AF517270, AF517270, AF517271, AF517271, AF517272, AF517272, AF517273, AF517273, AF517274, AF517274, AF517275, AF517275, AF517276, AF517276, AF517277, AF517277, AF517278, AF517278, AF517279, AF517279, AF517280, AF517280, AF517282, AF517282, AF517283, AF517283, AF517284, AF517284, AF517286, AF517286, AF517287, AF517287, AF517288, AF517288, AF517289, AF517289, AF517290, AF517290, AF517291, AF517291, AF517292, AF517292, AF517293, AF517293, AF517294, AF517294, AF517295, AF517295, AF517296, AF517296, AF517297, AF517297, AF517298, AF517299, AF517299, AF517300, AF517300, AF517301, AF517301, AF517302, AF517302, AF517303, AF517303, AF517304, AF517304, AF517305, AF517305, AF517306, AF517306, AF517307, AF517307, AF517309, AF517309, AF517310, AF517310, AF517311, AF517311, AF517312, AF517312, AF517313, AF517314, AF517314, AF517315, AF517316, AF517318, AF517318, AF517319, AF519421, AF519422, AF519423, AF519424, AF519425, AF519426, AF519427, AF519428, AF519429, AF519430, AF519431, AF519432, AF519433, AF519434, AF519435, AF519437, AF519438, AF519439, AF519440, AF519441, AF519442, AF520772, AF520773, AF527177, AF527178, AF527179, AF527180, AF527182, AF527183, AF527184, AF527185, AF527187, AF527188, AF527189, AF527192, AF527194, AF527195, AF527196, AF527197, AF527198, AF527200, AF527201, AF527202, AF527203, AF527204, AF527210, AF527215, AF527216, AF527220, AF527221, AF527224, AF527226, AF527229, AF527233, AF527234, AF527236, AF527237, AF527245, AF527246, AF527247, AF527252, AF527253, AF527258, AF527259, AF527260, AF527261, AF527262, AF527263, AF527268, AF527269, AF527270, AF527271, AF527274, AF527275, AF527279, AF527280, AF527281, AF527282, AF527283, AF527285, AF527288, AF527289, AF527290, AF527291, AF527297, AF527299, AF527300, AF527301, AF527302, AF527303, AF527304, AF527307, AF527308, AF527309, AF527310, AF527313, AF527314, AF527315, AF527316, AF527319, AF527320, AF527321, AF527322, AF527324, AF527325, AF527328, AF527329, AF527331, AF527332, AF527333, AF527334, AF527335, AF527336, AF527337, AF527338, AF527339, AF527343, AF527344, AF527345, AF527347, AF527348, AF527351, AF527355, AF527356, AF527357, AF527358, AF527359, AF527362, AF527364, AF527365, AF527366, AF529925, AF544406, AF544407, AF544408, AF544410, AF544412, AF544413, AF544415, AF544416, AF544418, AF544421, AF544425, AF544427, AF544432, AF544435, AF544439, AF544442, AF544443, AF544448, AF544449, AF544450, AF544453, AF544458, AF544459, AF544460, AF544464, AF544466, AF544467, AF544468, AF544469, AF544470, AF544474, AF544476, AF544477, AF544480, AF544482, AF544483, AF544485, AF544486, AF544487, AF544488, AF544489, AF544500, AF544503, AF544505, AF544506, AF544507, AF544509, AF544510, AF544513, AF544518, AF544520, AF544522, AF544523, AF544524, AF544526, AF544527, AF544529, AF544531, AF544533, AF544534, AF544541, AF544542, AF544545, AF544548, AF544554, AF544555, AF544565, AF544567, AF544568, AF544569, AF544571, AF544572, AF544574, AF544575, AF544576, AF544580, AF544582, AF544583, AF544584, AF544586, AF544587, AF544588, AF544590, AF544593, AF544594, AF544595, AF544596, AF544597, AF544598, AF544600, AF544601, AF544602, AF544603, AF544604, AF544605, AF544607, AF544608, AF544614, AF544616, AF544617, AF544620, AF544621, AJ002376, AJ002498, AJ002501, AJ002502, AJ002504, AJ002505, AJ002506, AJ002507, AJ003206, AJ003207, AJ003208, AJ003209, AJ003210, AJ003211, AJ006287, AJ006287, AJ010412, AJ010413, AJ010415, AJ010421, AJ010714, AJ010715, AJ010716, AJ010717, AJ010718, AJ010720, AJ010721, AJ010723, AJ012426, AJ012428, AJ012429, AJ012430, AJ012431, AJ012432, AJ012433, AJ012434, AJ012435, AJ102425, AJ225914, AJ225917, AJ225920, AJ225928, AJ225929, AJ225931, AJ225932, AJ225939, AJ225940, AJ225942, AJ225943, AJ225951, AJ225952, AJ279592, AJ279593, AJ279594, AJ279595, AJ279596, AJ279597, AJ279598, AJ279599, AJ279600, AJ279601, AJ279602, AJ279603, AJ279604, AJ279605, AJ279606, AJ279607, AJ279608, AJ279609, AJ279610, AJ279611, AJ279612, AJ279613, AJ279614, AJ279615, AJ279617, AJ279618, AJ279619, AJ279620, AJ279621, AJ279622, AJ279623, AJ279624, AJ279625, AJ279626, AJ279627, AJ279629, AJ279630, AJ279631, AJ279632, AJ279633, AJ279634, AJ279635, AJ279636, AJ279637, AJ279638, AJ279639, AJ279640, AJ279641, AJ279642, AJ279643, AJ279644, AJ279646, AJ279648, AJ279649, AJ279650, AJ279651, AJ279652, AJ279654, AJ279655, AJ279656, AJ279657, AJ279658, AJ279659, AJ279660, AJ279661, AJ279662, AJ279664, AJ279665, AJ279666, AJ279668, AJ279669, AJ279670, AJ279671, AJ279672, AJ279673, AJ279674, AJ279675, AJ279676, AJ279677, AJ279678, AJ279679, AJ279680, AJ279681, AJ279682, AJ279683, AJ279684, AJ279685, AJ287007, AJ287007, AJ287008, AJ287008, AJ287009, AJ287009, AJ287010, AJ287010, AJ287044, AJ287044, AJ287045, AJ287045, AJ287046, AJ287046, AJ287047, AJ287047, AJ287048, AJ287048, AJ287049, AJ287049, AJ287050, AJ287050, AJ287051, AJ287051, AJ287052, AJ401724, AJ401773, AJ401777, AJ401788, AJ401793, AJ401794, AJ401798, AJ401799, AJ401808, AJ401811, AJ401812, AJ401815, AJ401817, AJ401818, AJ401819, AJ401820, AJ401821, AJ401823, AJ401825, AJ401826, AJ401827, AJ401828, AJ401829, AJ401830, AJ401835, AJ401841, AJ401847, AJ401870, AJ401913, AJ401914, AJ401927, AJ401947, AJ401954, AJ401965, AJ401967, AJ401968, AJ401969, AJ401970, AJ401971, AJ401972, AJ401973, AJ401974, AJ401975, AJ401976, AJ401977, AJ404345, AJ404357, AJ404358, AJ404365, AJ404413, AJ404414, AJ404426, AJ405947, AJ405947, AJ405948, AJ405948, AJ405949, AJ405949, AJ405950, AJ405950, AJ405951, AJ405951, AJ405952, AJ405952, AJ405953, AJ405953, AJ405954, AJ405954, AJ405955, AJ405955, AJ405956, AJ405956, AJ405957, AJ405957, AJ405958, AJ405958, AJ405959, AJ405959, AJ405960, AJ405960, AJ405961, AJ405961, AJ405962, AJ405962, AJ405963, AJ405963, AJ405964, AJ405964, AJ405965, AJ405965, AJ405966, AJ405966, AJ405967, AJ405967, AJ405968, AJ405968, AJ419427, AJ419427, AJ419428, AJ419428, AJ419429, AJ419429, AJ419431, AJ419431, AJ419432, AJ419432, AJ419433, AJ419433, AJ419434, AJ419434, AJ419435, AJ419435, AJ419436, AJ419436, AJ419437, AJ419437, AJ419439, AJ419439, AJ419440, AJ419440, AJ419442, AJ419442, AJ419447, AJ419447, AJ419448, AJ419448, AJ419453, AJ419453, AJ419456, AJ419456, AJ419458, AJ419458, AJ419460, AJ419460, AJ419461, AJ419461, AJ419464, AJ419464, AJ419465, AJ419467, AJ419467, AJ419468, AJ419468, AJ419469, AJ419469, AJ419470, AJ419470, AJ419475, AJ419475, AJ419478, AJ419478, AJ419479, AJ419479, AJ419480, AJ419480, AJ419481, AJ419481, AJ419482, AJ419482, AJ419483, AJ419483, AJ419485, AJ419485, AJ419486, AJ419486, AJ419488, AJ419488, AJ419489, AJ419489, AJ419490, AJ419490, AJ419491, AJ419491, AJ419492, AJ419492, AJ419496, AJ419496, AJ419497, AJ419497, AJ419499, AJ419499, AJ419503, AJ419503, AJ419504, AJ419504, AJ419505, AJ419505, AJ419506, AJ419506, AJ419508, AJ419508, AJ419511, AJ419511, AJ419512, AJ419512, AJ419513, AJ419513, AJ419515, AJ419515, AJ419520, AJ419520, AJ419521, AJ419521, AJ419522, AJ419522, AJ419523, AJ419523, AJ577726, AJ577726, AJ577730, AJ577731, AJ577731, AJ577732, AJ577732, AJ577735, AJ577735, AJ577736, AJ577736, AJ577737, AJ577742, AJ577743, AJ577744, AJ577744, AJ577858, AJ577858, AJ577859, AJ577859, AJ577860, AJ577861, AJ577861, AJ577862, AJ577862, AJ577866, AJ577866, AJ577869, AJ577869, AJ577871, AJ577871, AJ577872, AJ577872, AJ577873, AJ577873, AJ577874, AJ577874, AJ577875, AJ577875, AJ577876, AJ577876, AJ577877, AJ577877, AJ577878, AJ577881, AJ577881, AJ577882, AJ577883, AJ577885, AJ577888, AJ577888, AJ577890, AJ577890, AJ577891, AJ577891, AJ577892, AJ577892, AJ577893, AJ577893, AJ577897, AJ577899, AJ577899, AJ577900, AJ577906, AJ577906, AJ577907, AJ577907, AJ577908, AJ577909, AJ577910, AJ577917, AJ577917, AJ577927, AJ577927, AJ577928, AJ577929, AJ577929, AJ577930, AJ577930, AJ577931, AJ577931, AJ577932, AJ577932, AJ577933, AJ577933, AJ577934, AJ577934, AJ577935, AJ577936, AJ577936, AJ577941, AJ577941, AJ577942, AJ577942, AJ577943, AJ577943, AJ577945, AJ577945, AJ577946, AJ577946, AJ577948, AJ577948, AJ577949, AJ577949, AJ577950, AJ577950, AJ577951, AJ577951, AJ577952, AJ577952, AJ577955, AJ577955, AJ577957, AJ577957, AJ577960, AJ577960, AJ577961, AJ577961, AJ577962, AJ577962, AJ577970, AJ577970, AJ577972, AJ577972, AJ577976, AJ577981, AJ577984, AJ577984, AJ577985, AJ577986, AJ577986, AJ577988, AJ577988, AJ577993, AJ577993, AJ577994, AJ577994, AJ577995, AJ577995, AJ578157, AJ578157, AJ578158, AJ578158, AJ578161, AJ578162, AJ578162, AJ578166, AJ578166, AJ578167, AJ578167, AJ578168, AJ578171, AJ578171, AJ578172, AJ578172, AJ578175, AJ578175, AJ578179, AJ578179, AJ583740, AJ583740, AJ583743, AJ583743, AJ583744, AJ583744, AJ971091, AJ971091, AJ971092, AJ971092, AJ971093, AJ971093, AJ971097, AJ971097, AJ971098, AJ971098, AJ971099, AJ971101, AJ971101, AJ971103, AJ971103, AJ971104, AJ971104, AJ971105, AJ971105, AJ971106, AJ971106, AJ971107, AJ971107, AJ971108, AJ971108, AJ971109, AJ971109, AJ971110, AJ971110, AJ971111, AJ971111, AJ971112, AJ971112, AJ971113, AJ971113, AJ971114, AJ971114, AJ971115, AJ971115, AJ971116, AJ971116, AJ971117, AJ971117, AJ971118, AJ971118, AJ971119, AJ971119, AJ971120, AJ971120, AJ971121, AJ971121, AJ971122, AJ971122, AJ971123, AJ971123, AJ971124, AJ971124, AJ971125, AJ971125, AJ971126, AJ971126, AJ971127, AJ971127, AJ971128, AJ971128, AJ971129, AJ971130, AJ971130, AJ971131, AJ971131, AJ971132, AJ971132, AJ971133, AJ971133, AJ971135, AJ971136, AJ971136, AJ971137, AJ971140, AJ971140, AJ971141, AJ971141, AJ971142, AJ971142, AJ971143, AJ971143, AJ971144, AJ971144, AJ971268, AJ971268, AJ971269, AJ971269, AJ971276, AJ971276, AJ971277, AJ971277, AJ971278, AJ971278, AJ971279, AJ971279, AJ971280, AJ971280, AJ971281, AJ971281, AJ971282, AJ971282, AJ971283, AJ971283, AJ971284, AJ971284, AJ971285, AJ971285, AJ971286, AJ971286, AJ971287, AJ971287, AJ971288, AJ971288, AJ971289, AJ971289, AJ971290, AJ971290, AM040998, AM041007, AM041047, AM181791, AM181791, AY000040, AY000057, AY000237, AY000240, AY001095, AY001095, AY001108, AY001110, AY001213, AY001392, AY001394, AY001397, AY001416, AY001422, AY001446, AY001538, AY001541, AY001593, AY001602, AY001607, AY001619, AY001642, AY001764, AY001764, AY001820, AY001820, AY001889, AY001902, AY001902, AY001916, AY001917, AY001921, AY001923, AY001926, AY001930, AY001935, AY001946, AY001954, AY001957, AY001962, AY001977, AY001978, AY002012, AY002013, AY002039, AY002079, AY002080, AY002080, AY002147, AY002164, AY002278, AY002282, AY002295, AY002323, AY002347, AY002353, AY002364, AY002364, AY002368, AY002368, AY002372, AY002394, AY002394, AY002413, AY002424, AY002692, AY002694, AY002695, AY002722, AY002745, AY002745, AY002761, AY002762, AY002803, AY002804, AY002818, AY002822, AY002870, AY002873, AY002931, AY002953, AY002955, AY003056, AY003057, AY003060, AY003079, AY003092, AY003094, AY003148, AY003160, AY003294, AY003294, AY003295, AY003301, AY003309, AY003311, AY003315, AY003326, AY003332, AY003343, AY003344, AY003356, AY003357, AY003371, AY003383, AY003383, AY003384, AY003399, AY003413, AY003427, AY003460, AY003464, AY003528, AY003682, AY003695, AY003695, AY016812, AY016813, AY016814, AY016815, AY016816, AY016817, AY016818, AY016819, AY016820, AY016821, AY016822, AY016823, AY016824, AY016825, AY016826, AY016827, AY016828, AY016829, AY016830, AY016831, AY016832, AY016833, AY016834, AY016835, AY016836, AY016837, AY016838, AY016839, AY016840, AY016841, AY016842, AY016843, AY016844, AY016845, AY016846, AY016847, AY016848, AY016849, AY016850, AY016851, AY016852, AY016853, AY016854, AY016855, AY016856, AY016857, AY016858, AY016859, AY016860, AY016861, AY016862, AY016863, AY016864, AY016865, AY016866, AY016867, AY016868, AY016869, AY016870, AY016871, AY016872, AY016873, AY016874, AY016875, AY016876, AY016877, AY016878, AY016879, AY016880, AY016881, AY016882, AY016883, AY016884, AY016885, AY016886, AY016887, AY016888, AY016889, AY016890, AY016891, AY016892, AY016893, AY016894, AY016895, AY016896, AY016897, AY016899, AY016900, AY016901, AY016902, AY016903, AY016904, AY016905, AY016906, AY016907, AY016908, AY016909, AY016911, AY016912, AY016913, AY016914, AY016915, AY016916, AY016917, AY016918, AY016919, AY016920, AY016921, AY016922, AY016923, AY016924, AY016925, AY016926, AY016927, AY016928, AY016929, AY016930, AY016931, AY016932, AY016933, AY016934, AY016935, AY016936, AY016937, AY016938, AY016939, AY016940, AY016941, AY016942, AY016943, AY016944, AY016945, AY016946, AY016947, AY016948, AY016949, AY016950, AY016951, AY016952, AY016953, AY016954, AY016955, AY016956, AY016957, AY016958, AY016959, AY016960, AY016961, AY016962, AY016963, AY016964, AY016965, AY016966, AY016967, AY016968, AY016969, AY016970, AY016971, AY016972, AY016973, AY016974, AY016975, AY016976, AY016977, AY016978, AY016979, AY016980, AY016981, AY016982, AY016983, AY016984, AY016985, AY016986, AY016987, AY016988, AY016989, AY016990, AY016991, AY016992, AY016993, AY016994, AY016995, AY016996, AY016998, AY016999, AY017000, AY017001, AY017002, AY017003, AY017004, AY017005, AY017006, AY017007, AY017008, AY017010, AY017460, AY017460, AY017466, AY030412, AY030412, AY030413, AY030413, AY030419, AY030419, AY030420, AY030420, AY030421, AY030421, AY030424, AY030424, AY030426, AY030426, AY030427, AY030429, AY030429, AY030430, AY030430, AY030431, AY030431, AY030432, AY030432, AY030433, AY030433, AY030437, AY030438, AY030438, AY030442, AY030447, AY030447, AY030448, AY030449, AY030449, AY030450, AY030450, AY030451, AY030451, AY030461, AY030461, AY030471, AY030471, AY030478, AY030478, AY030487, AY030487, AY030489, AY030489, AY030491, AY030492, AY030492, AY030495, AY030495, AY030503, AY030503, AY030505, AY030507, AY030507, AY030510, AY030510, AY030512, AY030512, AY030517, AY030523, AY030523, AY030524, AY030524, AY030525, AY030526, AY030526, AY030527, AY030527, AY030531, AY030539, AY030541, AY030541, AY030542, AY030542, AY030546, AY030546, AY030547, AY030547, AY030552, AY030552, AY030554, AY030554, AY030556, AY030556, AY030557, AY030557, AY030561, AY030561, AY030566, AY030566, AY030567, AY030567, AY030571, AY030571, AY030574, AY030576, AY030580, AY030582, AY030582, AY030583, AY030583, AY030585, AY030585, AY030589, AY030589, AY030593, AY030593, AY030600, AY030602, AY030602, AY030612, AY030612, AY030617, AY030630, AY030630, AY030632, AY030632, AY030635, AY030635, AY030637, AY030637, AY030641, AY030641, AY030642, AY030642, AY030644, AY030644, AY030645, AY030645, AY030649, AY030649, AY030650, AY030651, AY030651, AY030657, AY030657, AY030658, AY030658, AY030659, AY030661, AY030661, AY030667, AY030667, AY030670, AY030670, AY030672, AY030672, AY030676, AY030677, AY030677, AY030685, AY030685, AY030688, AY030688, AY030692, AY030692, AY030696, AY030696, AY030697, AY030697, AY030702, AY030702, AY030704, AY030704, AY030706, AY030706, AY030716, AY030716, AY030720, AY030720, AY030727, AY030727, AY030730, AY030730, AY030732, AY030732, AY030734, AY030735, AY030735, AY030740, AY030740, AY030742, AY030742, AY030748, AY030748, AY030752, AY030752, AY030755, AY030755, AY030757, AY030758, AY030758, AY030761, AY030761, AY030763, AY030763, AY030765, AY030765, AY030767, AY030768, AY030768, AY030769, AY030769, AY030775, AY030775, AY030777, AY030777, AY030789, AY030789, AY030795, AY030795, AY030799, AY030799, AY030804, AY030804, AY030806, AY030808, AY030808, AY030811, AY030811, AY030813, AY030813, AY030824, AY030824, AY030829, AY030829, AY030831, AY030831, AY030834, AY030834, AY030835, AY030835, AY030836, AY030836, AY030844, AY030844, AY030847, AY030847, AY030848, AY030848, AY030849, AY030849, AY030851, AY030851, AY030853, AY030853, AY030864, AY030864, AY030865, AY030865, AY030866, AY030877, AY030877, AY030884, AY030884, AY030890, AY030890, AY030891, AY030891, AY030893, AY030893, AY030894, AY030894, AY030900, AY030900, AY030904, AY030905, AY030905, AY030907, AY030909, AY030909, AY030911, AY030924, AY030924, AY030925, AY030925, AY030929, AY030929, AY030930, AY030930, AY030936, AY030936, AY030937, AY030937, AY030938, AY030938, AY030944, AY030944, AY030946, AY030946, AY030948, AY030948, AY030952, AY030952, AY030956, AY030956, AY030957, AY030957, AY030961, AY030961, AY030966, AY030966, AY030967, AY030967, AY030968, AY030968, AY030969, AY030969, AY030973, AY030973, AY030974, AY030974, AY030978, AY030978, AY030980, AY030980, AY030982, AY030982, AY030985, AY030985, AY030987, AY030987, AY030996, AY030996, AY030997, AY030997, AY031000, AY031000, AY031002, AY031005, AY031005, AY031009, AY031009, AY031011, AY031011, AY031015, AY031015, AY031018, AY031018, AY031019, AY031019, AY031021, AY031021, AY031022, AY031022, AY031029, AY031029, AY031031, AY031031, AY031032, AY031032, AY031038, AY031038, AY031040, AY031040, AY031044, AY031044, AY031046, AY031046, AY031048, AY031048, AY031054, AY031054, AY031060, AY031060, AY031064, AY031064, AY031066, AY031066, AY031068, AY031068, AY031069, AY031069, AY031075, AY031075, AY031076, AY031076, AY031077, AY031077, AY031079, AY031079, AY031082, AY031082, AY031083, AY031083, AY031086, AY031086, AY031087, AY031087, AY031091, AY031091, AY031100, AY031100, AY031103, AY031103, AY031105, AY031105, AY031106, AY031106, AY031107, AY031107, AY031108, AY031108, AY031117, AY031117, AY031118, AY031118, AY031119, AY031119, AY031121, AY031123, AY031123, AY031125, AY031125, AY031131, AY031131, AY031132, AY031134, AY031134, AY031138, AY031138, AY031143, AY031143, AY031144, AY031144, AY031146, AY031147, AY031147, AY031150, AY031150, AY031155, AY031155, AY031157, AY031157, AY031163, AY031163, AY031164, AY031164, AY031165, AY031165, AY031170, AY031170, AY031171, AY031171, AY031178, AY031178, AY031180, AY031180, AY031188, AY031188, AY031192, AY031192, AY031193, AY031193, AY031200, AY031200, AY031201, AY031201, AY031202, AY031202, AY031204, AY031204, AY031207, AY031207, AY031212, AY031212, AY031226, AY031226, AY031238, AY031238, AY031241, AY031241, AY031242, AY031242, AY031247, AY031247, AY031250, AY031250, AY031255, AY031255, AY031257, AY031257, AY031261, AY031261, AY031263, AY031263, AY031264, AY031264, AY031268, AY031268, AY031271, AY031271, AY031272, AY031272, AY031279, AY031279, AY031289, AY031289, AY031290, AY031290, AY031291, AY031291, AY031292, AY031292, AY031296, AY031296, AY031298, AY031298, AY031303, AY031306, AY031306, AY031308, AY031308, AY031310, AY031311, AY031311, AY031316, AY031316, AY031319, AY031319, AY031326, AY031326, AY031328, AY031328, AY031329, AY031329, AY031342, AY031342, AY031344, AY031344, AY031349, AY031349, AY031350, AY031350, AY031352, AY031352, AY031359, AY031359, AY031361, AY031361, AY031362, AY031362, AY031363, AY031363, AY031367, AY031367, AY031372, AY031375, AY031375, AY031379, AY031379, AY031381, AY031381, AY031383, AY031383, AY031385, AY031385, AY031387, AY031387, AY031388, AY031388, AY031389, AY031389, AY031390, AY031390, AY031391, AY031391, AY031396, AY031396, AY031400, AY031400, AY031402, AY031402, AY031403, AY031406, AY031406, AY031413, AY031413, AY031416, AY031416, AY031426, AY031426, AY031430, AY031430, AY031435, AY031438, AY031438, AY031441, AY031441, AY031447, AY031447, AY031466, AY031466, AY031476, AY031476, AY031479, AY031479, AY031480, AY031480, AY031481, AY031481, AY031483, AY031483, AY031484, AY031484, AY031485, AY031485, AY031486, AY031486, AY031490, AY031490, AY031491, AY031495, AY031495, AY031498, AY031498, AY031500, AY031506, AY031506, AY031511, AY031511, AY031512, AY031512, AY031514, AY031514, AY031515, AY031515, AY031516, AY031516, AY031522, AY031522, AY031528, AY031528, AY031529, AY031533, AY031533, AY031534, AY031534, AY031536, AY031542, AY031542, AY031552, AY031552, AY031554, AY031554, AY031556, AY031557, AY031557, AY031565, AY031565, AY031575, AY031575, AY031577, AY031577, AY031587, AY031587, AY031588, AY031589, AY031589, AY031594, AY031594, AY031595, AY031595, AY031597, AY031597, AY031598, AY031598, AY031604, AY031604, AY031606, AY031606, AY031609, AY031609, AY031610, AY031610, AY031611, AY031611, AY031612, AY031612, AY031617, AY031617, AY031620, AY031620, AY031623, AY031623, AY031636, AY031640, AY031640, AY031645, AY031654, AY031654, AY031659, AY031659, AY031660, AY031660, AY031666, AY031666, AY031668, AY031670, AY031670, AY031677, AY031678, AY031684, AY031684, AY031685, AY031685, AY031694, AY031694, AY031698, AY031698, AY031702, AY031702, AY031719, AY031719, AY031723, AY031723, AY031724, AY031724, AY031733, AY031733, AY031741, AY031741, AY031745, AY031745, AY031748, AY031748, AY031760, AY031760, AY031763, AY031763, AY031764, AY031765, AY031765, AY031766, AY031766, AY031777, AY031777, AY031781, AY031781, AY031789, AY031789, AY031796, AY031796, AY031797, AY031797, AY031805, AY031805, AY031807, AY031807, AY031808, AY031808, AY031818, AY031818, AY031825, AY031829, AY031829, AY031834, AY031834, AY031835, AY031835, AY031836, AY031836, AY031845, AY031846, AY031846, AY031848, AY031848, AY031854, AY031854, AY031858, AY031858, AY031863, AY031863, AY031864, AY031864, AY031865, AY031866, AY031866, AY031868, AY031868, AY031872, AY031872, AY031873, AY031873, AY031875, AY031875, AY031891, AY031891, AY031894, AY031894, AY031895, AY031895, AY031896, AY031896, AY031901, AY031901, AY031902, AY031902, AY031908, AY031908, AY031912, AY031912, AY031928, AY031928, AY031931, AY031931, AY031932, AY031932, AY031934, AY031934, AY031935, AY031935, AY031941, AY031941, AY031944, AY031944, AY031963, AY031963, AY031969, AY031969, AY031970, AY031970, AY031971, AY031971, AY031976, AY031976, AY031977, AY031977, AY031990, AY031990, AY031998, AY031998, AY031999, AY031999, AY032002, AY032002, AY032007, AY032007, AY032008, AY032008, AY032017, AY032017, AY032018, AY032018, AY032021, AY032021, AY032022, AY032022, AY032023, AY032023, AY032025, AY032025, AY032035, AY032035, AY032039, AY032039, AY032041, AY032041, AY032043, AY032043, AY032053, AY032053, AY032055, AY032055, AY032067, AY032067, AY032075, AY032075, AY032078, AY032078, AY032087, AY032087, AY032089, AY032089, AY032092, AY032092, AY032093, AY032093, AY032096, AY032096, AY032097, AY032097, AY032099, AY032099, AY032100, AY032100, AY032102, AY032102, AY032106, AY032106, AY032107, AY032107, AY032109, AY032109, AY032131, AY032131, AY032133, AY032133, AY032137, AY032137, AY032140, AY032140, AY032141, AY032141, AY032142, AY032142, AY032148, AY032148, AY032159, AY032159, AY032161, AY032161, AY032163, AY032163, AY032164, AY032164, AY032177, AY032177, AY032179, AY032179, AY032194, AY032194, AY032196, AY032196, AY032197, AY032197, AY032202, AY032202, AY032204, AY032211, AY032211, AY032212, AY032212, AY032215, AY032215, AY032221, AY032222, AY032222, AY032224, AY032224, AY032225, AY032225, AY032226, AY032226, AY032228, AY032228, AY032229, AY032229, AY032234, AY032234, AY032235, AY032235, AY032238, AY032238, AY032242, AY032242, AY032254, AY032254, AY032266, AY032266, AY032271, AY032271, AY032274, AY032274, AY032283, AY032283, AY032288, AY032288, AY032294, AY032294, AY032296, AY032296, AY032298, AY032298, AY032305, AY032305, AY032306, AY032306, AY032307, AY032307, AY032312, AY032312, AY032314, AY032314, AY032315, AY032315, AY032324, AY032324, AY032326, AY032326, AY032330, AY032330, AY032331, AY032331, AY032332, AY032332, AY032344, AY032344, AY032347, AY032347, AY032348, AY032348, AY032351, AY032355, AY032355, AY032358, AY032358, AY032361, AY032361, AY032364, AY032364, AY032366, AY032366, AY032377, AY032377, AY032379, AY032379, AY032387, AY032387, AY032388, AY032388, AY032393, AY032393, AY032395, AY032395, AY032398, AY032398, AY032399, AY032399, AY032402, AY032402, AY032403, AY032403, AY032406, AY032406, AY032407, AY032407, AY032409, AY032409, AY032412, AY032412, AY032418, AY032418, AY032422, AY032422, AY032423, AY032423, AY032425, AY032425, AY032431, AY032431, AY032437, AY032439, AY032439, AY032441, AY032441, AY032442, AY032442, AY032443, AY032443, AY032444, AY032444, AY032445, AY032445, AY032446, AY032446, AY032447, AY032447, AY032453, AY032453, AY032456, AY032456, AY032457, AY032457, AY032458, AY032458, AY032460, AY032460, AY032461, AY032462, AY032462, AY032463, AY032463, AY032465, AY032465, AY032468, AY032468, AY032469, AY032469, AY032470, AY032470, AY032471, AY032471, AY032479, AY032479, AY032480, AY032480, AY032482, AY032482, AY032483, AY032483, AY032484, AY032484, AY032494, AY032494, AY032495, AY032495, AY032497, AY032497, AY032498, AY032498, AY032510, AY032510, AY032511, AY032511, AY032513, AY032513, AY032515, AY032515, AY032516, AY032516, AY032520, AY032520, AY032521, AY032522, AY032522, AY032523, AY032523, AY032524, AY032524, AY032527, AY032530, AY032530, AY032531, AY032531, AY032540, AY032540, AY032541, AY032541, AY032542, AY032542, AY032543, AY032547, AY032553, AY032553, AY032555, AY032555, AY032556, AY032556, AY032560, AY032560, AY032561, AY032561, AY032562, AY032562, AY032566, AY032566, AY032572, AY032572, AY032579, AY032579, AY032586, AY032586, AY042725, AY042726, AY042727, AY042728, AY042729, AY042730, AY042731, AY042732, AY042733, AY042734, AY042735, AY042736, AY042737, AY042738, AY042739, AY042740, AY042741, AY042742, AY042744, AY042745, AY042746, AY042747, AY042748, AY042749, AY042750, AY042751, AY042752, AY042753, AY042754, AY042755, AY042756, AY042757, AY042758, AY042759, AY042760, AY042761, AY042762, AY042763, AY042764, AY042765, AY042766, AY042767, AY042768, AY042769, AY042770, AY042771, AY042772, AY042773, AY042774, AY042775, AY042776, AY042777, AY042778, AY042779, AY042780, AY042781, AY042782, AY046879, AY046880, AY046881, AY046882, AY046883, AY047368, AY047370, AY047374, AY047382, AY047392, AY047394, AY047404, AY047408, AY047410, AY047413, AY047420, AY047425, AY047427, AY047429, AY047435, AY047441, AY047443, AY047445, AY047449, AY047450, AY047458, AY047460, AY047463, AY047466, AY047467, AY047470, AY047471, AY047474, AY049528, AY049529, AY049530, AY049531, AY049534, AY049535, AY049536, AY049537, AY049538, AY049539, AY049540, AY049541, AY049542, AY049543, AY049544, AY049545, AY049546, AY049547, AY049548, AY049549, AY049550, AY049551, AY049552, AY049553, AY049554, AY049555, AY049556, AY049557, AY049558, AY049559, AY049560, AY049561, AY049562, AY049563, AY049564, AY049565, AY049566, AY049567, AY049568, AY049569, AY049570, AY049571, AY049572, AY049573, AY049574, AY049575, AY049576, AY049577, AY049578, AY049579, AY049580, AY049581, AY049582, AY049583, AY049584, AY049585, AY049586, AY049587, AY049588, AY049589, AY049590, AY049591, AY049592, AY049593, AY049594, AY049595, AY049596, AY049597, AY049598, AY049599, AY049602, AY049603, AY049604, AY049605, AY049606, AY049607, AY049608, AY049609, AY049610, AY049611, AY049612, AY049613, AY049614, AY049615, AY049616, AY049617, AY049618, AY049619, AY049620, AY049621, AY049622, AY049623, AY049624, AY049625, AY049628, AY049629, AY049630, AY049631, AY049632, AY049633, AY049634, AY049635, AY049636, AY049637, AY049638, AY049639, AY049641, AY049642, AY049643, AY049644, AY049645, AY049650, AY049652, AY049653, AY049654, AY049655, AY049656, AY049657, AY049658, AY049659, AY049660, AY049661, AY049662, AY049663, AY049664, AY049665, AY049668, AY049669, AY049670, AY049671, AY049674, AY049675, AY049676, AY049677, AY049680, AY049681, AY049682, AY049683, AY049684, AY049685, AY049686, AY049687, AY049688, AY049689, AY049692, AY049693, AY049694, AY049695, AY049696, AY049697, AY049698, AY049699, AY049700, AY049701, AY049702, AY049703, AY051198, AY102490, AY102490, AY133491, AY133493, AY133495, AY133497, AY133499, AY133500, AY133502, AY133504, AY133506, AY133508, AY133510, AY133511, AY133513, AY134447, AY134449, AY134451, AY134453, AY134455, AY134456, AY134458, AY134460, AY134462, AY134464, AY134466, AY134468, AY140610, AY140611, AY140612, AY140613, AY145782, AY145782, AY145783, AY145783, AY145784, AY145784, AY145785, AY145785, AY145786, AY145786, AY145787, AY145787, AY145788, AY145788, AY145789, AY145789, AY145790, AY145790, AY145791, AY145791, AY145792, AY145792, AY145793, AY145793, AY145794, AY145794, AY145795, AY145795, AY145796, AY145796, AY145797, AY145797, AY145798, AY145798, AY145799, AY145799, AY145800, AY145800, AY145801, AY145801, AY145802, AY145802, AY145803, AY145803, AY145804, AY145804, AY145805, AY145805, AY145806, AY145806, AY145807, AY145807, AY145808, AY145808, AY145809, AY145809, AY145810, AY145810, AY145811, AY145812, AY145812, AY145814, AY145814, AY145815, AY145815, AY145817, AY145818, AY145819, AY145821, AY145821, AY145822, AY165185, AY165185, AY165188, AY165188, AY165189, AY165189, AY165190, AY165190, AY165191, AY165191, AY165192, AY165192, AY165193, AY165193, AY165195, AY165195, AY165197, AY165197, AY165198, AY165198, AY165199, AY165199, AY165200, AY165200, AY165201, AY165201, AY165202, AY165202, AY165203, AY165203, AY165204, AY165204, AY165210, AY165210, AY165217, AY165217, AY165222, AY165222, AY165227, AY165227, AY165228, AY165228, AY165229, AY165229, AY165231, AY165231, AY165232, AY165232, AY165233, AY165233, AY165234, AY165234, AY165236, AY165236, AY165237, AY165237, AY165238, AY165238, AY165241, AY165241, AY165242, AY165242, AY165244, AY165244, AY165245, AY165245, AY165246, AY165246, AY165247, AY165247, AY165250, AY165250, AY165251, AY165251, AY165254, AY165254, AY165256, AY165256, AY165259, AY165259, AY165261, AY165261, AY165262, AY165262, AY165264, AY165264, AY165267, AY165267, AY165268, AY165268, AY165269, AY165269, AY165270, AY165270, AY165271, AY165271, AY165273, AY165273, AY165274, AY165275, AY165275, AY165277, AY165277, AY165278, AY165278, AY165280, AY165280, AY165282, AY165282, AY173951, AY173951, AY173952, AY173952, AY173953, AY173953, AY173954, AY173954, AY173955, AY173955, AY173956, AY173956, AY173958, AY173959, AY173959, AY173960, AY173960, AY182785, AY182786, AY182789, AY182790, AY182791, AY182792, AY182793, AY182796, AY182797, AY182798, AY182799, AY182800, AY182801, AY182802, AY182804, AY182805, AY182810, AY182811, AY182813, AY182814, AY182815, AY182816, AY182817, AY182819, AY182820, AY182822, AY182823, AY182824, AY182825, AY182828, AY182830, AY182831, AY182832, AY182833, AY207857, AY209546, AY209546, AY209553, AY209553, AY209562, AY209562, AY209569, AY209569, AY209577, AY209577, AY209584, AY209584, AY209593, AY209593, AY209601, AY209601, AY209609, AY209609, AY209617, AY209617, AY209625, AY209625, AY209633, AY209633, AY209641, AY209641, AY209650, AY209650, AY209657, AY209657, AY209665, AY209665, AY209673, AY209673, AY209682, AY209682, AY209692, AY209692, AY209699, AY209699, AY209705, AY209705, AY209713, AY209713, AY209720, AY209720, AY209727, AY209727, AY209735, AY209735, AY209743, AY209743, AY209750, AY209750, AY209758, AY209758, AY209765, AY209765, AY209773, AY209773, AY209781, AY209781, AY209790, AY209790, AY209806, AY209806, AY209813, AY209813, AY209819, AY209819, AY209826, AY209826, AY209835, AY209835, AY209845, AY209845, AY209862, AY209862, AY209869, AY209877, AY209877, AY209885, AY209885, AY209893, AY209893, AY213197, AY213198, AY213199, AY213202, AY213203, AY213204, AY213205, AY213206, AY213207, AY213208, AY213209, AY213210, AY213211, AY213212, AY213213, AY213216, AY213217, AY213218, AY213219, AY213222, AY213223, AY213224, AY213225, AY213226, AY213228, AY213229, AY213230, AY213231, AY213234, AY213237, AY213243, AY213244, AY213245, AY213246, AY213248, AY213249, AY213251, AY213252, AY213253, AY213254, AY213255, AY213256, AY213257, AY213258, AY213259, AY213260, AY213261, AY213262, AY213263, AY213264, AY213265, AY213266, AY213268, AY213269, AY213270, AY213271, AY213272, AY213273, AY213274, AY213275, AY213276, AY213278, AY213279, AY213280, AY213281, AY213282, AY213283, AY213284, AY213285, AY213286, AY213287, AY213288, AY213289, AY213290, AY213291, AY213292, AY213293, AY213294, AY213295, AY213296, AY213297, AY213298, AY213299, AY213300, AY213301, AY213302, AY213303, AY213304, AY213305, AY213306, AY213307, AY213308, AY213309, AY213310, AY213311, AY213312, AY213313, AY213314, AY213315, AY213316, AY213317, AY213318, AY213323, AY213324, AY213325, AY213327, AY213328, AY213329, AY213330, AY213331, AY213333, AY213334, AY213335, AY213336, AY213337, AY213338, AY213339, AY213341, AY213342, AY213343, AY213344, AY213345, AY213348, AY213349, AY213352, AY213353, AY213354, AY213362, AY213363, AY213364, AY213365, AY213368, AY213369, AY213372, AY213373, AY213378, AY213379, AY213380, AY213381, AY213382, AY213383, AY213392, AY213398, AY213399, AY213400, AY213401, AY213403, AY213407, AY213408, AY213409, AY213410, AY213412, AY213417, AY213418, AY213419, AY213420, AY213421, AY213423, AY213426, AY213427, AY213428, AY213433, AY213434, AY213450, AY213451, AY213452, AY213453, AY213454, AY213461, AY213462, AY213463, AY213464, AY213466, AY213468, AY213471, AY213472, AY213475, AY213476, AY213482, AY213483, AY213485, AY213492, AY213494, AY213495, AY213501, AY213502, AY213503, AY213504, AY213505, AY213512, AY213512, AY213513, AY213513, AY213514, AY213514, AY213516, AY213516, AY213517, AY213517, AY213524, AY213524, AY213526, AY213526, AY213528, AY213528, AY213529, AY213529, AY213530, AY213530, AY213531, AY213531, AY213532, AY213532, AY213533, AY213533, AY213535, AY213535, AY213537, AY213537, AY213538, AY213538, AY213539, AY213539, AY213540, AY213540, AY213541, AY213541, AY213543, AY213543, AY213545, AY213545, AY213546, AY213546, AY213547, AY213547, AY213548, AY213548, AY213549, AY213551, AY213551, AY222050, AY222051, AY222052, AY222054, AY222055, AY222056, AY222057, AY222058, AY222059, AY222060, AY222061, AY222063, AY222064, AY222066, AY222067, AY222069, AY237980, AY238100, AY238126, AY238184, AY242863, AY242864, AY242865, AY242866, AY242867, AY242868, AY242869, AY242870, AY242871, AY242872, AY242874, AY242875, AY242876, AY242877, AY242878, AY242879, AY242881, AY242882, AY242883, AY242884, AY242885, AY242886, AY242887, AY242888, AY242889, AY242890, AY242891, AY242892, AY242893, AY242894, AY242895, AY242896, AY242897, AY242898, AY242899, AY242900, AY242901, AY242902, AY242903, AY242904, AY242905, AY242906, AY242907, AY242908, AY242909, AY242910, AY242911, AY242912, AY242913, AY242914, AY242915, AY242916, AY242918, AY242919, AY242921, AY242922, AY242923, AY242924, AY242925, AY242927, AY242928, AY242929, AY242930, AY242931, AY242932, AY242933, AY242934, AY242935, AY242937, AY242938, AY242939, AY242940, AY242941, AY242942, AY242943, AY242944, AY242945, AY242946, AY242947, AY242948, AY263493, AY263493, AY263494, AY263494, AY263496, AY263496, AY263498, AY263498, AY267259, AY267259, AY267260, AY267260, AY267261, AY267261, AY267262, AY267262, AY267263, AY267263, AY267264, AY267264, AY267265, AY267265, AY267266, AY267266, AY267267, AY267267, AY267268, AY267268, AY267269, AY267269, AY267270, AY267270, AY267271, AY267271, AY267272, AY267272, AY267273, AY267273, AY267274, AY267274, AY267275, AY267275, AY267276, AY267277, AY267277, AY267278, AY267278, AY267279, AY267279, AY267280, AY267280, AY267281, AY267281, AY267282, AY267282, AY267283, AY267283, AY267284, AY267284, AY267285, AY267285, AY267286, AY267286, AY267287, AY267287, AY267288, AY267288, AY267289, AY267289, AY267290, AY267290, AY267291, AY267291, AY267292, AY267292, AY267293, AY267293, AY267294, AY267294, AY267295, AY267295, AY267296, AY267296, AY267297, AY267297, AY267298, AY267298, AY267299, AY267299, AY267300, AY267300, AY267301, AY267301, AY267302, AY267302, AY267303, AY267303, AY267304, AY267304, AY267305, AY267305, AY267306, AY267306, AY267307, AY267307, AY267308, AY267308, AY267309, AY267309, AY267310, AY267310, AY267311, AY267311, AY267312, AY267312, AY267313, AY267313, AY267314, AY267314, AY267315, AY267315, AY267316, AY267316, AY267317, AY267317, AY267318, AY267318, AY267319, AY267319, AY267320, AY267320, AY267322, AY267322, AY267323, AY267323, AY267324, AY267324, AY267325, AY267325, AY267326, AY267326, AY267327, AY267327, AY267328, AY267328, AY267329, AY267329, AY267784, AY267784, AY267787, AY267787, AY267788, AY267788, AY267789, AY267789, AY267790, AY267790, AY267791, AY267791, AY285014, AY285015, AY285017, AY285018, AY285020, AY285021, AY285023, AY285024, AY285026, AY285027, AY285029, AY285030, AY285032, AY285033, AY285035, AY285036, AY285038, AY285039, AY285041, AY285042, AY285044, AY285045, AY285047, AY285048, AY285050, AY285051, AY285053, AY285054, AY285056, AY285057, AY285059, AY285060, AY285062, AY285063, AY285065, AY285066, AY285068, AY285069, AY285071, AY285072, AY285074, AY285075, AY285077, AY285078, AY285080, AY285081, AY285083, AY285084, AY285086, AY285087, AY285089, AY285090, AY285092, AY285093, AY285095, AY285097, AY285098, AY285099, AY285100, AY285102, AY285103, AY285104, AY285105, AY285107, AY285109, AY285110, AY285111, AY285112, AY285113, AY285128, AY285132, AY285135, AY285138, AY285142, AY285145, AY285147, AY285148, AY285150, AY285151, AY285153, AY285154, AY285156, AY285157, AY285159, AY305902, AY305904, AY305908, AY305909, AY305910, AY305911, AY305913, AY305916, AY305917, AY305918, AY305921, AY305925, AY305926, AY305927, AY305928, AY305931, AY305936, AY305937, AY305938, AY305942, AY305943, AY305946, AY305952, AY305953, AY305955, AY305956, AY305957, AY305959, AY305961, AY305963, AY305964, AY305970, AY305973, AY305984, AY305985, AY305988, AY305993, AY305994, AY305995, AY305996, AY305997, AY305998, AY313299, AY313301, AY313302, AY313303, AY313306, AY313308, AY313309, AY313312, AY313314, AY313315, AY313316, AY313317, AY313319, AY313320, AY313321, AY313323, AY313324, AY313325, AY313326, AY313327, AY313328, AY313329, AY313330, AY313331, AY313332, AY313333, AY313334, AY313335, AY313336, AY313337, AY313338, AY313339, AY313340, AY313341, AY313343, AY313344, AY313345, AY313347, AY313348, AY313349, AY313350, AY313351, AY313352, AY313353, AY313354, AY313355, AY313359, AY313360, AY313362, AY313363, AY313364, AY313367, AY313369, AY313370, AY313371, AY313372, AY313373, AY313374, AY313375, AY313377, AY313379, AY313381, AY313382, AY313383, AY313384, AY313385, AY313386, AY313387, AY313388, AY313389, AY313390, AY313391, AY313392, AY313393, AY313394, AY313397, AY313398, AY313399, AY313400, AY313401, AY313402, AY313403, AY313404, AY313405, AY313406, AY313407, AY313408, AY313409, AY313410, AY313412, AY313413, AY313414, AY313415, AY313416, AY313419, AY313421, AY313422, AY313423, AY313424, AY313425, AY313426, AY313427, AY313428, AY313429, AY313430, AY313431, AY313432, AY313433, AY313434, AY313435, AY313436, AY313437, AY313438, AY313439, AY313440, AY313441, AY313442, AY313443, AY313444, AY313445, AY313446, AY313447, AY351704, AY351705, AY351706, AY351712, AY351722, AY351764, AY351766, AY351771, AY358069, AY358069, AY358071, AY358072, AY358073, AY358073, AY390038, AY390040, AY390041, AY390042, AY390044, AY390046, AY390047, AY390048, AY390050, AY390053, AY390054, AY390055, AY390056, AY390057, AY390058, AY390059, AY390060, AY390062, AY390063, AY390064, AY390065, AY390066, AY390068, AY390069, AY390071, AY390072, AY390073, AY390075, AY390086, AY390089, AY390090, AY390091, AY390092, AY390093, AY390094, AY390095, AY390096, AY390097, AY390100, AY390101, AY390102, AY390103, AY390104, AY390105, AY390107, AY390108, AY390109, AY390110, AY390111, AY390112, AY390113, AY390114, AY390115, AY390116, AY390117, AY390118, AY390119, AY390120, AY390121, AY390122, AY390123, AY390126, AY390128, AY390129, AY390130, AY390131, AY390132, AY390133, AY390134, AY390136, AY390137, AY390138, AY390139, AY390140, AY390141, AY390142, AY390143, AY390144, AY390145, AY390146, AY390147, AY390148, AY390149, AY390150, AY390151, AY390152, AY390153, AY390154, AY390155, AY390156, AY390157, AY390158, AY390159, AY390161, AY390162, AY390163, AY390164, AY390165, AY390166, AY390167, AY390168, AY390169, AY390170, AY390171, AY390172, AY390173, AY390174, AY435375, AY441814, AY441824, AY441867, AY441877, AY441879, AY441910, AY441912, AY441926, AY441928, AY441937, AY441952, AY441954, AY444196, AY444196, AY444197, AY444197, AY444198, AY444198, AY444199, AY444199, AY444200, AY444200, AY444201, AY444201, AY444202, AY444202, AY444203, AY444203, AY444204, AY444204, AY444205, AY444205, AY444206, AY444206, AY444207, AY444207, AY444291, AY444299, AY444300, AY444301, AY444302, AY444812, AY444812, AY455778, AY455783, AY461483, AY461484, AY461485, AY461486, AY461487, AY461488, AY461489, AY461490, AY461492, AY461493, AY461497, AY461498, AY461499, AY461500, AY461503, AY461504, AY492784, AY492784, AY492786, AY510095, AY510096, AY510097, AY510098, AY510099, AY510100, AY510101, AY527818, AY527819, AY527819, AY527823, AY527823, AY527824, AY527825, AY527825, AY527826, AY527826, AY527827, AY527828, AY527828, AY527829, AY527830, AY527830, AY527831, AY527833, AY527833, AY527834, AY527838, AY527838, AY527839, AY527842, AY527842, AY527843, AY527844, AY527844, AY527845, AY527849, AY527849, AY527850, AY527852, AY527852, AY527853, AY527854, AY527854, AY527855, AY527858, AY527858, AY527860, AY527861, AY527861, AY527862, AY527862, AY527863, AY527864, AY527864, AY527865, AY527866, AY527866, AY527867, AY527868, AY527868, AY527869, AY527871, AY527871, AY527872, AY527873, AY527873, AY527874, AY527877, AY527877, AY527878, AY527882, AY527882, AY527883, AY527884, AY527884, AY527885, AY527887, AY527887, AY527888, AY527892, AY527892, AY527893, AY527894, AY527894, AY527895, AY527896, AY527896, AY527897, AY527899, AY527899, AY527900, AY527903, AY527903, AY527905, AY527907, AY527907, AY527908, AY527909, AY527909, AY527910, AY527912, AY527912, AY527913, AY527915, AY527915, AY527916, AY527918, AY527918, AY527919, AY527923, AY527923, AY527924, AY527927, AY527927, AY527928, AY527932, AY527932, AY527933, AY527935, AY527935, AY527936, AY527938, AY527938, AY527939, AY527943, AY527943, AY527944, AY527944, AY527945, AY527948, AY527948, AY527949, AY527950, AY527950, AY527951, AY527953, AY527953, AY527954, AY527955, AY527955, AY527957, AY527959, AY527959, AY527960, AY527962, AY527962, AY527963, AY527964, AY527964, AY527965, AY527968, AY527968, AY527970, AY527971, AY527971, AY527973, AY527974, AY527974, AY527976, AY527980, AY527980, AY527981, AY527983, AY527983, AY527984, AY527986, AY527986, AY527987, AY527990, AY527990, AY527991, AY527992, AY527992, AY527993, AY527996, AY527996, AY527997, AY528000, AY528000, AY528002, AY528003, AY528003, AY528004, AY528007, AY528007, AY528008, AY528010, AY528010, AY528012, AY528013, AY528013, AY528014, AY528017, AY528017, AY528018, AY528021, AY528021, AY528022, AY528023, AY528023, AY528024, AY528025, AY528025, AY528026, AY528030, AY528030, AY528031, AY528032, AY528032, AY528033, AY528034, AY528034, AY528035, AY528039, AY528039, AY528040, AY528042, AY528042, AY528043, AY528047, AY528047, AY528048, AY528052, AY528052, AY528053, AY528055, AY528055, AY528056, AY528060, AY528060, AY528061, AY528064, AY528064, AY528065, AY528069, AY528069, AY528070, AY528074, AY528074, AY528075, AY528076, AY528076, AY528082, AY528084, AY528084, AY528085, AY528088, AY528088, AY528089, AY528090, AY528090, AY528091, AY528094, AY528094, AY528095, AY528096, AY528096, AY528097, AY528101, AY528101, AY528102, AY528103, AY528103, AY528104, AY528108, AY528108, AY528109, AY528111, AY528111, AY528112, AY528113, AY528113, AY528114, AY528118, AY528118, AY528119, AY528120, AY528120, AY528121, AY528123, AY528123, AY528124, AY528128, AY528128, AY528129, AY528133, AY528133, AY528134, AY528137, AY528137, AY528138, AY528139, AY528139, AY528140, AY528143, AY528143, AY528145, AY528148, AY528148, AY528150, AY528153, AY528153, AY528155, AY528156, AY528156, AY528157, AY528160, AY528160, AY528161, AY528163, AY528163, AY528164, AY528165, AY528165, AY528166, AY528167, AY528167, AY528168, AY528172, AY528172, AY528173, AY528174, AY528174, AY528175, AY528177, AY528177, AY528178, AY528181, AY528181, AY528182, AY528185, AY528185, AY528186, AY528190, AY528190, AY528191, AY528194, AY528194, AY528195, AY528196, AY528196, AY528197, AY528201, AY528201, AY528202, AY528204, AY528204, AY528205, AY528205, AY528206, AY528207, AY528208, AY528208, AY528209, AY528212, AY528212, AY528214, AY528218, AY528218, AY528219, AY528220, AY528220, AY528221, AY528222, AY528222, AY529539, AY529546, AY529580, AY529585, AY529586, AY529588, AY529597, AY529603, AY529604, AY529611, AY548236, AY548236, AY548237, AY548237, AY548238, AY548238, AY548239, AY548239, AY548240, AY548240, AY548241, AY548241, AY548242, AY548242, AY548243, AY548243, AY548244, AY548244, AY548245, AY548245, AY548246, AY548246, AY548247, AY548247, AY548248, AY548248, AY548249, AY548249, AY548250, AY548251, AY548251, AY548252, AY548252, AY548253, AY548253, AY548254, AY548254, AY548255, AY548255, AY548256, AY548256, AY548257, AY548257, AY548258, AY548258, AY548259, AY548259, AY548260, AY548261, AY548261, AY548262, AY548262, AY548263, AY548263, AY548264, AY548264, AY548265, AY548266, AY548267, AY548268, AY548268, AY548269, AY548269, AY548270, AY548270, AY548271, AY548271, AY548272, AY548272, AY548273, AY548273, AY548275, AY548276, AY548276, AY548277, AY548277, AY548278, AY548278, AY548279, AY548279, AY548280, AY548280, AY548281, AY548281, AY548282, AY548282, AY548283, AY548283, AY548284, AY548284, AY548285, AY548285, AY548286, AY548286, AY548287, AY548287, AY548288, AY548288, AY548289, AY548289, AY556812, AY569830, AY569832, AY569833, AY569835, AY569836, AY569837, AY569838, AY569839, AY569840, AY569842, AY569843, AY569844, AY569845, AY569846, AY569847, AY569848, AY569849, AY569850, AY569851, AY569853, AY569854, AY569855, AY569858, AY569859, AY569860, AY569861, AY569863, AY569864, AY569865, AY569866, AY569867, AY569868, AY569869, AY569870, AY569873, AY569874, AY569875, AY569877, AY569879, AY569880, AY569881, AY569883, AY569885, AY569886, AY569887, AY569888, AY569890, AY569891, AY569892, AY569893, AY569894, AY569895, AY569896, AY569897, AY569899, AY569902, AY569903, AY569904, AY569905, AY569906, AY569907, AY569908, AY569909, AY569910, AY569911, AY569912, AY569913, AY569914, AY569915, AY569916, AY569917, AY569918, AY569919, AY569920, AY569921, AY569923, AY569924, AY569925, AY569926, AY569927, AY569928, AY569929, AY569930, AY569931, AY569932, AY569933, AY569934, AY569935, AY569936, AY569937, AY569938, AY569939, AY569940, AY569941, AY569943, AY569944, AY569945, AY569946, AY569947, AY569948, AY569949, AY569950, AY569953, AY569954, AY569955, AY569956, AY569957, AY569958, AY569959, AY569960, AY569961, AY569962, AY569963, AY569964, AY569965, AY569967, AY569968, AY569969, AY587958, AY587959, AY587960, AY587961, AY587962, AY587963, AY587964, AY587965, AY587966, AY587967, AY587968, AY587969, AY587970, AY587971, AY587972, AY587973, AY587974, AY598799, AY598800, AY598801, AY598803, AY598804, AY598805, AY598806, AY598807, AY598808, AY598811, AY598812, AY598814, AY598815, AY605558, AY605559, AY605562, AY605563, AY605590, AY605634, AY605637, AY605638, AY643137, AY643139, AY643154, AY643183, AY643184, AY643189, AY643195, AY643200, AY643204, AY643225, AY643227, AY643254, AY643269, AY643271, AY643273, AY677379, AY677387, AY677392, AY677393, AY677434, AY677464, AY677509, AY677510, AY677524, AY677570, AY713408, AY713408, AY713409, AY713409, AY713410, AY713410, AY713411, AY713411, AY713412, AY713412, AY736108, AY736110, AY736110, AY736111, AY736113, AY736113, AY736114, AY736115, AY736115, AY736116, AY736117, AY736117, AY789993, AY789993, AY789996, AY789996, AY789997, AY789997, AY789998, AY790002, AY790002, AY790003, AY790003, AY790004, AY790006, AY790006, AY795904, AY795905, AY795905, AY795908, AY795908, AY796421, AY796423, AY796425, AY796428, AY796429, AY796430, AY796433, AY796435, AY796436, AY796438, AY796441, AY796442, AY796444, AY796452, AY796456, AY796467, AY796476, AY796478, AY796480, AY796485, AY796489, AY796492, AY796493, AY796494, AY796496, AY796500, AY796501, AY796504, AY796505, AY796508, AY796509, AY796510, AY796513, AY796515, AY796516, AY796519, AY796521, AY796525, AY796526, AY796527, AY796529, AY796532, AY796535, AY796538, AY796540, AY796543, AY796551, AY796555, AY796559, AY796563, AY796565, AY796570, AY796572, AY796576, AY796581, AY796582, AY796584, AY796585, AY796586, AY796595, AY796597, AY796598, AY796601, AY796604, AY796605, AY796608, AY796609, AY796610, AY796619, AY796622, AY796623, AY796624, AY796629, AY796630, AY796631, AY796636, AY796638, AY796641, AY796642, AY796643, AY796644, AY796652, AY796653, AY796657, AY796659, AY796660, AY796662, AY796665, AY796666, AY796670, AY796673, AY796674, AY796677, AY796678, AY796681, AY796683, AY796684, AY796686, AY796688, AY796693, AY796698, AY796701, AY796703, AY796707, AY796711, AY796713, AY796715, AY796716, AY796717, AY796718, AY796720, AY796721, AY796723, AY796727, AY796728, AY796730, AY796732, AY796734, AY796738, AY796739, AY796743, AY796744, AY796746, AY796747, AY796750, AY796751, AY796752, AY796753, AY796755, AY796758, AY796762, AY796764, AY796765, AY796769, AY796771, AY796773, AY796774, AY796775, AY796779, AY796781, AY796784, AY796785, AY796787, AY796788, AY796789, AY796792, AY796795, AY796796, AY796798, AY796799, AY796801, AY796804, AY796805, AY796809, AY796810, AY796813, AY796814, AY796815, AY796816, AY796819, AY796822, AY796824, AY796825, AY796827, AY796829, AY796834, AY796835, AY796836, AY796837, AY796839, AY796840, AY796843, AY796845, AY796847, AY796848, AY796849, AY796852, AY796853, AY796854, AY796856, AY796857, AY796858, AY796860, AY796864, AY796865, AY796866, AY796867, AY796869, AY796870, AY796871, AY796874, AY796876, AY796877, AY796880, AY796881, AY796882, AY796883, AY796884, AY796886, AY796887, AY796888, AY796891, AY796896, AY796897, AY796903, AY796907, AY796910, AY796911, AY796914, AY796921, AY796924, AY796925, AY796926, AY796927, AY796928, AY796929, AY796930, AY796931, AY796934, AY796936, AY796937, AY796938, AY796942, AY796944, AY796946, AY796948, AY796950, AY796951, AY796952, AY796954, AY796957, AY796959, AY796960, AY796962, AY796964, AY796965, AY796967, AY796969, AY796971, AY796975, AY796976, AY796977, AY796980, AY796981, AY796983, AY796986, AY796987, AY796991, AY796994, AY796995, AY796998, AY796999, AY797000, AY797001, AY797002, AY797003, AY797004, AY797005, AY797006, AY797008, AY797009, AY797010, AY797011, AY797012, AY797013, AY797014, AY797015, AY797016, AY797017, AY797018, AY797020, AY797021, AY797023, AY797024, AY797025, AY797026, AY797029, AY797030, AY797031, AY797034, AY797035, AY797042, AY797046, AY797048, AY797050, AY797054, AY797055, AY797056, AY797057, AY797058, AY797060, AY797061, AY797063, AY797065, AY797066, AY797067, AY797068, AY797072, AY797074, AY797078, AY797079, AY797081, AY797082, AY797086, AY797087, AY797090, AY797091, AY797092, AY797094, AY797095, AY797097, AY797100, AY797102, AY797103, AY797106, AY797107, AY797113, AY797115, AY797116, AY797117, AY797119, AY797121, AY797124, AY797127, AY797130, AY797131, AY797132, AY797134, AY797135, AY797138, AY797140, AY797141, AY797142, AY797145, AY797146, AY797148, AY797149, AY797151, AY797152, AY797154, AY797157, AY797159, AY797166, AY797168, AY797169, AY797171, AY797173, AY797174, AY797176, AY797179, AY797181, AY797183, AY797185, AY797186, AY797187, AY797188, AY797192, AY797196, AY797197, AY797199, AY797200, AY797203, AY797204, AY797206, AY797207, AY797209, AY797213, AY797214, AY797216, AY797218, AY797220, AY797221, AY797223, AY797226, AY797227, AY797228, AY797229, AY797230, AY797231, AY797232, AY797233, AY797234, AY797235, AY797238, AY797239, AY797240, AY797241, AY797244, AY797246, AY797247, AY797248, AY797249, AY797250, AY797252, AY797253, AY797255, AY797256, AY797258, AY797260, AY797261, AY797263, AY797264, AY797266, AY797267, AY797268, AY797270, AY797272, AY797273, AY797275, AY797278, AY797281, AY797283, AY797284, AY797286, AY797288, AY797289, AY797290, AY797291, AY797292, AY797293, AY797294, AY797295, AY797296, AY797297, AY797298, AY797300, AY797302, AY797305, AY797306, AY797308, AY797312, AY797314, AY797315, AY797316, AY797318, AY797319, AY797324, AY797327, AY797328, AY797329, AY797332, AY797333, AY797336, AY797340, AY797341, AY797342, AY797344, AY797345, AY797347, AY797348, AY797349, AY797350, AY797353, AY797354, AY797355, AY797357, AY797359, AY797360, AY797361, AY797362, AY797363, AY797364, AY797366, AY797367, AY797368, AY797369, AY797371, AY797372, AY797373, AY797374, AY797375, AY797376, AY797379, AY797380, AY797382, AY797383, AY797385, AY797386, AY797387, AY797388, AY797390, AY797391, AY797393, AY797396, AY797398, AY797400, AY797401, AY797402, AY797403, AY797404, AY797406, AY797408, AY797409, AY797410, AY797411, AY797412, AY797413, AY797414, AY797415, AY797417, AY797419, AY797421, AY797424, AY797428, AY797429, AY797431, AY797432, AY797434, AY797437, AY797438, AY797439, AY797441, AY797442, AY797447, AY797448, AY797450, AY797451, AY797452, AY797453, AY797454, AY797457, AY797458, AY797460, AY797461, AY797463, AY797464, AY797465, AY797467, AY797468, AY797470, AY797472, AY797473, AY797475, AY797478, AY797479, AY797480, AY797481, AY797482, AY797483, AY797485, AY797486, AY797487, AY797488, AY797489, AY797490, AY797492, AY797495, AY797496, AY797497, AY797499, AY797502, AY797503, AY797504, AY797506, AY797507, AY797508, AY797509, AY797510, AY797511, AY797513, AY797515, AY797517, AY797518, AY797521, AY797523, AY797524, AY797526, AY797528, AY797531, AY797532, AY797534, AY797535, AY797537, AY797538, AY797539, AY797541, AY797542, AY797544, AY797545, AY797546, AY797547, AY797549, AY797550, AY797552, AY797553, AY797555, AY797558, AY797559, AY797562, AY797569, AY797571, AY797574, AY797575, AY797576, AY797577, AY797579, AY797580, AY797582, AY797584, AY797585, AY797587, AY797589, AY797591, AY797593, AY797594, AY797595, AY797596, AY797597, AY797598, AY797600, AY797602, AY797604, AY797605, AY797607, AY797610, AY797611, AY797613, AY797614, AY797615, AY797616, AY797618, AY797619, AY797620, AY797621, AY797622, AY797623, AY797625, AY797627, AY797628, AY797630, AY797631, AY797632, AY797635, AY797638, AY797639, AY797640, AY797641, AY797644, AY797645, AY797646, AY797648, AY797649, AY797650, AY797651, AY797653, AY797656, AY797658, AY797659, AY797660, AY797661, AY797663, AY797664, AY797666, AY797669, AY797673, AY797675, AY797677, AY797679, AY797680, AY797681, AY797682, AY797683, AY797685, AY797686, AY797688, AY797690, AY797692, AY797693, AY797694, AY797695, AY797696, AY797698, AY797699, AY797701, AY797702, AY797703, AY797705, AY797706, AY797707, AY797709, AY797710, AY797713, AY797716, AY797717, AY797719, AY797720, AY797721, AY797724, AY797726, AY797727, AY797728, AY797729, AY797730, AY797733, AY797734, AY797737, AY797738, AY797739, AY797740, AY797742, AY797745, AY797746, AY797748, AY797749, AY797750, AY797752, AY797753, AY797756, AY797757, AY797759, AY797761, AY797762, AY797764, AY797766, AY797767, AY797770, AY797772, AY797774, AY797775, AY797777, AY797778, AY797779, AY797780, AY797781, AY797782, AY797783, AY797785, AY797786, AY797787, AY797788, AY797789, AY797793, AY797794, AY797795, AY797796, AY797797, AY797798, AY797800, AY797801, AY797802, AY797803, AY797804, AY797805, AY797806, AY797812, AY797814, AY797816, AY797818, AY797819, AY797820, AY797821, AY797822, AY797825, AY797826, AY797828, AY797829, AY797830, AY797831, AY797833, AY797834, AY797836, AY797837, AY797838, AY797839, AY797840, AY797841, AY797842, AY797843, AY797844, AY797845, AY797846, AY797847, AY797848, AY797849, AY797851, AY797853, AY797855, AY797856, AY797857, AY797858, AY797859, AY797861, AY797863, AY797866, AY797867, AY797869, AY797870, AY797872, AY797874, AY797876, AY797877, AY797878, AY797880, AY797882, AY797883, AY797884, AY797885, AY797886, AY797887, AY797888, AY797889, AY797890, AY797891, AY797892, AY797893, AY797894, AY797895, AY797896, AY797898, AY797899, AY797900, AY797902, AY797903, AY797905, AY797907, AY797908, AY797910, AY797911, AY797912, AY797913, AY797915, AY797917, AY797918, AY797920, AY797921, AY797923, AY797924, AY797928, AY797930, AY797931, AY797932, AY797933, AY797934, AY797936, AY797937, AY797939, AY797942, AY797943, AY797944, AY797946, AY797947, AY797948, AY797949, AY797952, AY797953, AY797955, AY797959, AY797960, AY797961, AY797963, AY797964, AY797965, AY797968, AY797971, AY797974, AY797975, AY797976, AY797978, AY797979, AY797980, AY797982, AY797983, AY797984, AY797985, AY797987, AY797989, AY797990, AY797993, AY797994, AY797995, AY797996, AY797997, AY797998, AY797999, AY798000, AY798001, AY798003, AY798006, AY798007, AY798009, AY798012, AY798013, AY798015, AY798018, AY798019, AY798021, AY798023, AY798025, AY798027, AY798028, AY798030, AY798032, AY798034, AY798035, AY798037, AY798038, AY798040, AY798041, AY798042, AY798044, AY798045, AY798046, AY798049, AY798050, AY798051, AY798052, AY798053, AY798054, AY798055, AY798057, AY798058, AY798059, AY798060, AY798062, AY798064, AY798065, AY798066, AY798067, AY798068, AY798069, AY798070, AY798071, AY798072, AY798073, AY798075, AY798077, AY798078, AY798079, AY798080, AY798081, AY798083, AY798085, AY798086, AY798087, AY798089, AY798090, AY798091, AY798092, AY798094, AY798095, AY798096, AY798099, AY798102, AY798104, AY798105, AY798106, AY798107, AY798108, AY798109, AY798110, AY798112, AY798114, AY798115, AY798116, AY798118, AY798119, AY798120, AY798121, AY798122, AY798123, AY798124, AY798125, AY798126, AY798127, AY798128, AY798130, AY798131, AY798132, AY798133, AY798134, AY798135, AY798137, AY798138, AY798139, AY798140, AY798141, AY798142, AY798143, AY798144, AY798145, AY798146, AY798148, AY798149, AY798150, AY798151, AY798152, AY798153, AY798154, AY798155, AY798157, AY798158, AY798159, AY798160, AY798161, AY798162, AY798163, AY798164, AY798165, AY798169, AY798170, AY798171, AY798172, AY798173, AY798174, AY798175, AY798176, AY798177, AY798178, AY798179, AY798180, AY798181, AY798182, AY798183, AY798184, AY798185, AY798186, AY798187, AY798188, AY798189, AY798190, AY798191, AY798192, AY798193, AY798194, AY798197, AY798198, AY798199, AY798200, AY798202, AY798203, AY798204, AY798206, AY798207, AY798208, AY798209, AY798210, AY798211, AY798212, AY798213, AY798214, AY798215, AY798216, AY798218, AY798219, AY798220, AY798223, AY798224, AY798225, AY798226, AY798227, AY798228, AY798229, AY798230, AY798231, AY798232, AY798233, AY798234, AY798235, AY798236, AY798237, AY798239, AY798240, AY798241, AY798242, AY798243, AY798244, AY798245, AY798246, AY798247, AY798248, AY798249, AY798250, AY798251, AY798252, AY798253, AY798254, AY798256, AY798257, AY798258, AY798259, AY798260, AY798261, AY798262, AY798263, AY798264, AY798265, AY798267, AY798270, AY798271, AY798272, AY798273, AY798274, AY798275, AY798276, AY798277, AY798278, AY798279, AY798280, AY798281, AY798282, AY798283, AY798284, AY798285, AY798286, AY798287, AY798288, AY798289, AY798290, AY798291, AY798292, AY798293, AY798294, AY798295, AY798296, AY798297, AY798298, AY798299, AY798301, AY798302, AY798303, AY798304, AY798305, AY798306, AY798307, AY798308, AY798310, AY798311, AY798312, AY798313, AY798314, AY798315, AY798316, AY798317, AY798318, AY798319, AY798320, AY798321, AY798324, AY798335, AY798345, AY798349, AY798355, AY798356, AY798362, AY798382, AY798409, AY798413, AY798416, AY798417, AY798418, AY798420, AY798422, AY798423, AY798424, AY798426, AY798427, AY798428, AY798429, AY798432, AY798433, AY798435, AY798436, AY798437, AY798450, AY798451, AY798479, AY798480, AY798481, AY798483, AY798484, AY798486, AY798489, AY798491, AY798492, AY798494, AY798496, AY798497, AY800656, AY800659, AY800662, AY800664, AY800665, AY800666, AY800669, AY800672, AY800673, AY800675, AY800677, AY800685, AY800689, AY800701, AY800703, AY800704, AY800707, AY800709, AY800714, AY800718, AY800721, AY800725, AY800729, AY800734, AY800736, AY800737, AY800740, AY800744, AY800745, AY800748, AY800749, AY800752, AY800753, AY800754, AY800757, AY800760, AY800762, AY800764, AY800768, AY800770, AY800771, AY800774, AY800775, AY800776, AY800778, AY800779, AY800781, AY800784, AY800786, AY800790, AY800799, AY800802, AY800806, AY800812, AY800814, AY800816, AY800821, AY800823, AY800827, AY800830, AY800833, AY800835, AY800836, AY800844, AY800846, AY800847, AY800850, AY800853, AY800855, AY800857, AY800858, AY800859, AY800860, AY800862, AY800867, AY800872, AY800874, AY800877, AY800878, AY800886, AY800887, AY800892, AY800894, AY800897, AY800902, AY800903, AY800912, AY800913, AY800917, AY800918, AY800920, AY800922, AY800925, AY800927, AY800928, AY800931, AY800934, AY800936, AY800937, AY800938, AY800940, AY800946, AY800952, AY800956, AY800959, AY800961, AY800969, AY800972, AY800976, AY800978, AY800979, AY800980, AY800981, AY800982, AY800984, AY800989, AY800990, AY800992, AY800994, AY800996, AY801000, AY801005, AY801007, AY801009, AY801012, AY801013, AY801014, AY801015, AY801016, AY801020, AY801024, AY801026, AY801027, AY801031, AY801033, AY801035, AY801036, AY801037, AY801040, AY801043, AY801046, AY801047, AY801049, AY801050, AY801051, AY801053, AY801056, AY801057, AY801059, AY801061, AY801063, AY801064, AY801067, AY801070, AY801071, AY801072, AY801073, AY801074, AY801077, AY801080, AY801081, AY801082, AY801084, AY801086, AY801091, AY801092, AY801093, AY801094, AY801095, AY801098, AY801099, AY801102, AY801104, AY801105, AY801106, AY801107, AY801110, AY801111, AY801113, AY801114, AY801115, AY801119, AY801120, AY801121, AY801122, AY801123, AY801124, AY801125, AY801127, AY801128, AY801129, AY801130, AY801131, AY801132, AY801135, AY801136, AY801137, AY801139, AY801143, AY801144, AY801148, AY801155, AY801159, AY801161, AY801164, AY801170, AY801174, AY801175, AY801176, AY801177, AY801178, AY801180, AY801181, AY801183, AY801184, AY801189, AY801191, AY801194, AY801197, AY801199, AY801202, AY801205, AY801208, AY801209, AY801211, AY801214, AY801216, AY801217, AY801219, AY801221, AY801223, AY801224, AY801226, AY801230, AY801231, AY801232, AY801235, AY801236, AY801238, AY801241, AY801246, AY801249, AY801250, AY801251, AY801252, AY801253, AY801254, AY801255, AY801256, AY801257, AY801258, AY801259, AY801260, AY801261, AY801262, AY801264, AY801265, AY801266, AY801267, AY801269, AY801270, AY801273, AY801274, AY801275, AY801276, AY801277, AY801280, AY801281, AY801282, AY801284, AY801285, AY801292, AY801295, AY801298, AY801299, AY801303, AY801304, AY801305, AY801306, AY801307, AY801309, AY801311, AY801313, AY801314, AY801315, AY801319, AY801320, AY801324, AY801325, AY801327, AY801331, AY801332, AY801336, AY801338, AY801339, AY801342, AY801344, AY801347, AY801349, AY801352, AY801353, AY801359, AY801361, AY801362, AY801363, AY801366, AY801368, AY801371, AY801373, AY801375, AY801378, AY801379, AY801380, AY801382, AY801384, AY801385, AY801388, AY801390, AY801392, AY801395, AY801396, AY801398, AY801399, AY801401, AY801402, AY801403, AY801406, AY801408, AY801415, AY801417, AY801418, AY801420, AY801422, AY801423, AY801424, AY801426, AY801429, AY801431, AY801433, AY801435, AY801436, AY801437, AY801441, AY801445, AY801446, AY801448, AY801449, AY801452, AY801453, AY801455, AY801456, AY801458, AY801462, AY801463, AY801465, AY801467, AY801469, AY801470, AY801472, AY801473, AY801476, AY801477, AY801478, AY801479, AY801481, AY801482, AY801483, AY801484, AY801485, AY801486, AY801489, AY801490, AY801491, AY801492, AY801495, AY801497, AY801498, AY801499, AY801500, AY801502, AY801503, AY801505, AY801506, AY801507, AY801509, AY801511, AY801513, AY801515, AY801516, AY801517, AY801518, AY801520, AY801522, AY801524, AY801527, AY801530, AY801532, AY801534, AY801536, AY801538, AY801539, AY801540, AY801541, AY801542, AY801543, AY801544, AY801545, AY801547, AY801548, AY801550, AY801552, AY801554, AY801557, AY801558, AY801560, AY801563, AY801565, AY801566, AY801567, AY801569, AY801570, AY801575, AY801577, AY801578, AY801579, AY801580, AY801583, AY801584, AY801586, AY801590, AY801591, AY801593, AY801595, AY801596, AY801598, AY801599, AY801600, AY801603, AY801604, AY801605, AY801607, AY801609, AY801610, AY801611, AY801612, AY801613, AY801615, AY801616, AY801617, AY801618, AY801620, AY801621, AY801622, AY801623, AY801624, AY801625, AY801626, AY801629, AY801630, AY801632, AY801633, AY801635, AY801636, AY801637, AY801639, AY801640, AY801642, AY801645, AY801647, AY801648, AY801650, AY801651, AY801652, AY801653, AY801655, AY801657, AY801658, AY801659, AY801660, AY801661, AY801662, AY801663, AY801664, AY801666, AY801668, AY801670, AY801672, AY801673, AY801674, AY801678, AY801679, AY801683, AY801684, AY801686, AY801689, AY801690, AY801691, AY801692, AY801694, AY801695, AY801700, AY801701, AY801702, AY801704, AY801705, AY801706, AY801707, AY801708, AY801711, AY801712, AY801714, AY801715, AY801717, AY801718, AY801719, AY801721, AY801722, AY801724, AY801726, AY801727, AY801729, AY801732, AY801733, AY801734, AY801735, AY801736, AY801737, AY801739, AY801740, AY801741, AY801742, AY801743, AY801744, AY801745, AY801747, AY801748, AY801749, AY801752, AY801755, AY801756, AY801757, AY801759, AY801760, AY801761, AY801762, AY801763, AY801764, AY801766, AY801768, AY801769, AY801770, AY801773, AY801775, AY801778, AY801780, AY801783, AY801784, AY801786, AY801787, AY801789, AY801790, AY801792, AY801793, AY801795, AY801796, AY801797, AY801798, AY801800, AY801802, AY801803, AY801805, AY801806, AY801808, AY801811, AY801812, AY801815, AY801822, AY801824, AY801827, AY801828, AY801829, AY801831, AY801832, AY801833, AY801834, AY801836, AY801838, AY801839, AY801841, AY801843, AY801846, AY801847, AY801848, AY801849, AY801850, AY801851, AY801853, AY801855, AY801857, AY801858, AY801860, AY801863, AY801864, AY801866, AY801868, AY801869, AY801870, AY801871, AY801873, AY801874, AY801875, AY801876, AY801878, AY801880, AY801882, AY801883, AY801885, AY801886, AY801887, AY801890, AY801893, AY801894, AY801895, AY801898, AY801900, AY801901, AY801903, AY801904, AY801905, AY801906, AY801908, AY801911, AY801913, AY801914, AY801915, AY801917, AY801919, AY801920, AY801922, AY801923, AY801925, AY801929, AY801931, AY801933, AY801935, AY801936, AY801937, AY801938, AY801941, AY801942, AY801944, AY801946, AY801948, AY801949, AY801950, AY801951, AY801952, AY801954, AY801955, AY801957, AY801958, AY801959, AY801962, AY801963, AY801965, AY801966, AY801969, AY801972, AY801973, AY801975, AY801976, AY801977, AY801980, AY801982, AY801983, AY801984, AY801985, AY801986, AY801989, AY801990, AY801993, AY801994, AY801995, AY801996, AY801998, AY802001, AY802002, AY802005, AY802006, AY802007, AY802009, AY802010, AY802014, AY802015, AY802019, AY802020, AY802022, AY802024, AY802025, AY802028, AY802030, AY802032, AY802033, AY802035, AY802036, AY802037, AY802038, AY802039, AY802040, AY802041, AY802043, AY802044, AY802046, AY802047, AY802048, AY802052, AY802053, AY802054, AY802055, AY802056, AY802059, AY802060, AY802061, AY802062, AY802063, AY802064, AY802066, AY802072, AY802074, AY802076, AY802078, AY802079, AY802080, AY802081, AY802082, AY802085, AY802086, AY802087, AY802088, AY802089, AY802090, AY802091, AY802093, AY802094, AY802095, AY802096, AY802097, AY802098, AY802099, AY802100, AY802102, AY802103, AY802104, AY802105, AY802106, AY802107, AY802108, AY802110, AY802112, AY802114, AY802115, AY802116, AY802117, AY802118, AY802120, AY802122, AY802125, AY802126, AY802128, AY802129, AY802131, AY802132, AY802133, AY802134, AY802136, AY802137, AY802138, AY802140, AY802142, AY802143, AY802145, AY802146, AY802147, AY802148, AY802149, AY802150, AY802151, AY802152, AY802153, AY802154, AY802155, AY802156, AY802158, AY802159, AY802160, AY802162, AY802163, AY802165, AY802167, AY802168, AY802170, AY802171, AY802172, AY802173, AY802177, AY802178, AY802180, AY802181, AY802183, AY802184, AY802188, AY802190, AY802191, AY802192, AY802193, AY802194, AY802196, AY802198, AY802201, AY802202, AY802203, AY802205, AY802206, AY802207, AY802208, AY802211, AY802212, AY802213, AY802214, AY802218, AY802219, AY802220, AY802222, AY802223, AY802224, AY802227, AY802230, AY802233, AY802234, AY802235, AY802237, AY802238, AY802239, AY802241, AY802242, AY802243, AY802244, AY802246, AY802248, AY802249, AY802252, AY802253, AY802254, AY802256, AY802257, AY802258, AY802259, AY802260, AY802261, AY802262, AY802264, AY802267, AY802268, AY802270, AY802273, AY802274, AY802276, AY802279, AY802280, AY802282, AY802284, AY802286, AY802288, AY802289, AY802291, AY802293, AY802295, AY802296, AY802298, AY802299, AY802301, AY802302, AY802303, AY802305, AY802306, AY802307, AY802310, AY802311, AY802312, AY802313, AY802315, AY802316, AY802318, AY802319, AY802320, AY802321, AY802322, AY802325, AY802326, AY802328, AY802329, AY802330, AY802331, AY802332, AY802333, AY802334, AY802336, AY802337, AY802338, AY802339, AY802340, AY802341, AY802343, AY802345, AY802346, AY802347, AY802349, AY802350, AY802351, AY802352, AY802354, AY802355, AY802356, AY802359, AY802362, AY802363, AY802364, AY802365, AY802366, AY802367, AY802368, AY802369, AY802370, AY802371, AY802372, AY802374, AY802375, AY802376, AY802378, AY802379, AY802380, AY802381, AY802382, AY802383, AY802384, AY802385, AY802386, AY802387, AY802388, AY802390, AY802391, AY802392, AY802393, AY802394, AY802397, AY802398, AY802399, AY802400, AY802401, AY802402, AY802403, AY802404, AY802405, AY802406, AY802408, AY802409, AY802410, AY802411, AY802412, AY802413, AY802414, AY802415, AY802417, AY802418, AY802419, AY802420, AY802421, AY802422, AY802423, AY802424, AY802425, AY802429, AY802430, AY802431, AY802432, AY802433, AY802434, AY802435, AY802436, AY802437, AY802438, AY802439, AY802440, AY802441, AY802442, AY802443, AY802444, AY802445, AY802446, AY802447, AY802448, AY802449, AY802450, AY802451, AY802452, AY802453, AY802456, AY802457, AY802458, AY802459, AY802461, AY802462, AY802463, AY802465, AY802466, AY802467, AY802468, AY802469, AY802470, AY802471, AY802472, AY802473, AY802474, AY802475, AY802477, AY802478, AY802479, AY802482, AY802483, AY802484, AY802485, AY802486, AY802487, AY802488, AY802489, AY802490, AY802491, AY802492, AY802493, AY802494, AY802495, AY802496, AY802498, AY802499, AY802500, AY802501, AY802502, AY802503, AY802504, AY802505, AY802506, AY802507, AY802508, AY802509, AY802510, AY802511, AY802512, AY802513, AY802514, AY802516, AY802517, AY802518, AY802519, AY802520, AY802521, AY802522, AY802523, AY802524, AY802525, AY802527, AY802529, AY802532, AY802533, AY802534, AY802535, AY802536, AY802537, AY802538, AY802539, AY802540, AY802541, AY802542, AY802543, AY802544, AY802545, AY802546, AY802547, AY802548, AY802549, AY802550, AY802551, AY802552, AY802553, AY802554, AY802555, AY802556, AY802557, AY802558, AY802559, AY802560, AY802561, AY802562, AY802564, AY802565, AY802566, AY802567, AY802568, AY802569, AY802570, AY802571, AY802573, AY802574, AY802575, AY802576, AY802577, AY802578, AY802579, AY802580, AY802581, AY802582, AY802583, AY802584, AY802585, AY802586, AY802604, AY802608, AY802617, AY802618, AY802644, AY802648, AY802677, AY802680, AY802683, AY802684, AY802685, AY802686, AY802687, AY802688, AY802689, AY802692, AY802694, AY802695, AY802697, AY802699, AY802700, AY802701, AY802702, AY802703, AY802707, AY802708, AY802709, AY802711, AY802712, AY802713, AY802715, AY802717, AY802718, AY802726, AY802756, AY802758, AY845747, AY845747, AY845793, AY845793, AY872282, AY872283, AY872284, AY872285, AY872286, AY872287, AY872288, AY872289, AY872290, AY872291, AY872292, AY872293, AY872294, AY872295, AY872296, AY872297, AY872298, AY872299, AY872300, AY872301, AY872302, AY872303, AY872304, AY872305, AY872306, AY872307, AY872308, AY872309, AY872310, AY872311, AY872312, AY872313, AY872314, AY872315, AY872316, AY872317, AY872318, AY872319, AY872320, AY872321, AY872322, AY872323, AY872324, AY872325, AY872326, AY872332, AY872333, AY872334, AY872335, AY872336, AY872337, AY872338, AY872339, AY872340, AY872341, AY872342, AY872343, AY872344, AY872345, AY872346, AY872347, AY872348, AY872349, AY872350, AY872351, AY872352, AY872353, AY872354, AY872355, AY872356, AY872357, AY872358, AY872359, AY872360, AY872361, AY872362, AY872363, AY872364, AY872365, AY872366, AY872367, AY872368, AY872369, AY872370, AY872371, AY872372, AY872373, AY872374, AY872375, AY872376, AY872377, AY872378, AY872379, AY872380, AY872386, AY872387, AY872388, AY872389, AY904522, AY904523, AY904538, AY904542, AY904550, AY904555, AY904559, AY904562, AY904568, AY904581, AY904597, AY904599, AY904611, AY904615, AY904616, AY904617, AY904623, AY904638, AY904642, AY904650, AY904658, AY904661, AY904668, AY904681, AY904697, AY904699, AY904711, AY904715, AY904716, AY904717, AY940215, AY940216, AY940217, AY940218, AY940219, AY940220, AY940221, AY940222, AY940223, AY940224, AY940225, AY940226, AY940227, AY940228, AY940229, AY940230, AY940231, AY940232, AY940233, AY940234, AY940235, AY940236, AY940237, AY940238, AY940239, AY940240, AY940241, AY940242, AY940243, AY940244, AY940245, AY940246, AY940247, AY940248, AY940249, AY940250, AY940251, AY940252, AY940253, AY940254, AY940255, AY940256, AY940257, AY940258, AY940259, AY940260, AY940261, AY940262, AY940263, AY940264, AY940265, AY940315, AY967803, AY967804, AY967805, AY967805, AY971636, AY971637, AY971638, AY971640, AY971641, AY971642, AY971644, AY971645, AY971647, AY971649, AY971650, AY971651, AY971652, AY999349, AY999349, AY999350, AY999350, AY999351, AY999351, AY999352, AY999352, AY999353, AY999353, AY999354, AY999354, AY999355, AY999356, AY999356, AY999359, AY999359, AY999360, AY999360, AY999361, AY999361, AY999362, AY999362, AY999363, AY999363, AY999364, AY999364, AY999365, AY999365, AY999366, AY999366, AY999367, AY999367, AY999368, AY999368, AY999369, AY999370, AY999370, AY999371, AY999371, AY999372, AY999372, AY999373, AY999373, AY999374, AY999374, AY999375, AY999375, AY999376, AY999376, AY999377, AY999379, AY999380, AY999380, AY999381, AY999381, AY999382, AY999383, AY999383, AY999384, AY999384, AY999385, AY999385, AY999387, AY999387, AY999389, AY999389, AY999390, AY999390, AY999391, AY999391, AY999392, AY999392, AY999393, AY999393, AY999394, AY999394, AY999395, AY999395, AY999396, AY999396, AY999397, AY999397, AY999398, AY999398, AY999399, AY999399, AY999400, AY999400, AY999401, AY999402, AY999402, AY999403, AY999403, AY999404, AY999404, AY999405, AY999405, AY999406, AY999406, AY999407, AY999407, AY999408, AY999408, AY999409, AY999409, AY999410, AY999410, AY999411, AY999411, AY999412, AY999412, AY999413, AY999413, AY999414, AY999414, AY999415, AY999415, AY999417, AY999417, AY999418, AY999418, AY999419, AY999419, AY999420, AY999420, AY999421, AY999421, AY999422, AY999422, AY999423, AY999423, AY999424, AY999424, AY999425, AY999425, AY999426, AY999426, AY999428, AY999428, AY999429, AY999429, AY999430, AY999430, AY999431, AY999431, AY999432, AY999432, AY999433, AY999433, AY999434, AY999434, AY999435, AY999435, AY999437, AY999438, AY999439, AY999439, AY999440, AY999440, AY999441, AY999441, AY999442, AY999442, AY999443, AY999443, AY999444, AY999444, AY999445, AY999445, AY999446, AY999446, AY999447, AY999447, AY999448, AY999448, AY999450, AY999450, AY999451, AY999451, AY999452, AY999452, AY999454, AY999454, AY999455, AY999455, AY999456, AY999456, AY999458, AY999458, AY999459, AY999459, AY999460, AY999460, AY999461, AY999461, AY999462, AY999462, AY999464, AY999464, AY999465, AY999465, AY999466, AY999466, AY999468, AY999468, AY999469, AY999469, AY999470, AY999470, AY999471, AY999473, AY999473, AY999474, AY999474, AY999475, AY999475, AY999476, AY999477, AY999477, AY999478, AY999478, AY999479, AY999479, AY999480, AY999480, AY999482, AY999482, AY999483, AY999483, AY999484, AY999484, AY999485, AY999486, AY999486, AY999488, AY999488, AY999489, AY999489, AY999490, AY999490, AY999491, AY999491, AY999492, AY999492, AY999493, AY999493, AY999494, AY999494, AY999495, AY999495, AY999496, AY999496, AY999497, AY999497, AY999498, AY999498, AY999499, AY999499, AY999500, AY999500, AY999501, AY999501, AY999502, AY999502, AY999503, AY999503, AY999504, AY999504, AY999505, AY999505, AY999506, AY999506, AY999507, AY999507, AY999508, AY999508, AY999509, AY999509, AY999510, AY999510, AY999512, AY999512, AY999513, AY999513, AY999514, AY999514, AY999515, AY999515, AY999519, AY999519, AY999520, AY999520, AY999521, AY999521, AY999522, AY999522, AY999523, AY999523, AY999524, AY999524, AY999525, AY999526, AY999526, AY999527, AY999527, AY999528, AY999529, AY999529, AY999533, AY999533, AY999534, AY999534, AY999535, AY999535, AY999536, AY999536, AY999539, AY999539, AY999540, AY999540, AY999542, AY999542, AY999543, AY999543, AY999544, AY999544, AY999545, AY999545, AY999546, AY999546, AY999547, AY999547, AY999548, AY999548, AY999549, AY999549, AY999550, AY999550, AY999551, AY999551, AY999552, AY999552, AY999553, AY999553, AY999554, AY999554, AY999555, AY999555, AY999556, AY999556, AY999557, AY999557, AY999558, AY999558, AY999559, AY999559, AY999560, AY999560, AY999561, AY999561, AY999563, AY999563, AY999564, AY999564, AY999565, AY999565, AY999566, AY999566, AY999567, AY999567, AY999570, AY999570, AY999571, AY999571, AY999572, AY999572, AY999574, AY999575, AY999575, AY999576, AY999576, AY999577, AY999577, AY999578, AY999578, AY999579, AY999579, AY999580, AY999580, AY999581, AY999581, AY999582, AY999582, AY999583, AY999583, AY999584, AY999585, AY999585, AY999586, AY999586, AY999587, AY999587, AY999588, AY999590, AY999590, AY999591, AY999591, AY999592, AY999592, AY999593, AY999593, AY999594, AY999594, AY999595, AY999595, AY999596, AY999596, AY999597, AY999597, AY999598, AY999598, AY999599, AY999600, AY999600, AY999601, AY999601, AY999602, AY999602, AY999603, AY999604, AY999604, AY999605, AY999605, AY999607, AY999607, AY999608, AY999609, AY999609, AY999610, AY999610, AY999611, AY999611, AY999612, AY999612, AY999613, AY999613, AY999614, AY999614, AY999615, AY999615, AY999616, AY999617, AY999617, AY999618, AY999618, AY999619, AY999619, AY999620, AY999621, AY999621, AY999623, AY999623, AY999624, AY999624, AY999625, AY999626, AY999626, AY999627, AY999627, AY999628, AY999628, AY999629, AY999629, AY999630, AY999630, AY999631, AY999631, AY999632, AY999632, AY999633, AY999633, AY999634, AY999634, AY999635, AY999635, AY999636, AY999636, AY999637, AY999637, AY999638, AY999638, AY999639, AY999640, AY999640, AY999641, AY999641, AY999642, AY999642, AY999643, AY999643, AY999647, AY999647, AY999648, AY999648, AY999649, AY999649, AY999650, AY999650, AY999651, AY999651, AY999652, AY999652, AY999653, AY999653, AY999654, AY999654, AY999655, AY999655, AY999656, AY999657, AY999657, AY999658, AY999658, AY999659, AY999659, AY999661, AY999661, AY999662, AY999662, AY999663, AY999663, AY999664, AY999664, AY999665, AY999665, AY999666, AY999666, AY999667, AY999667, AY999668, AY999668, AY999669, AY999669, AY999670, AY999670, AY999671, AY999671, AY999672, AY999674, AY999674, AY999675, AY999675, AY999676, AY999676, AY999677, AY999677, AY999678, AY999678, AY999680, AY999680, AY999681, AY999681, D10112, D10112, DQ003015, DQ003015, DQ003016, DQ003016, DQ003017, DQ003017, DQ003018, DQ003018, DQ003019, DQ003019, DQ003020, DQ003020, DQ003021, DQ003021, DQ003022, DQ003022, DQ013397, DQ013444, DQ013541, DQ013649, DQ055197, DQ055197, DQ055198, DQ055198, DQ055200, DQ055200, DQ055202, DQ055202, DQ055207, DQ055207, DQ055208, DQ055208, DQ055209, DQ055209, DQ055211, DQ055211, DQ055213, DQ055213, DQ055214, DQ055214, DQ055215, DQ055215, DQ055216, DQ055216, DQ055218, DQ055218, DQ055220, DQ055220, DQ055227, DQ055227, DQ055228, DQ055228, DQ055236, DQ055236, DQ055275, DQ055280, DQ055284, DQ055284, DQ055286, DQ055286, DQ055287, DQ055287, DQ055292, DQ055292, DQ055294, DQ055294, DQ055296, DQ055296, DQ055300, DQ055300, DQ055305, DQ055305, DQ055309, DQ055309, DQ055312, DQ055312, DQ055314, DQ055314, DQ055316, DQ055316, DQ055317, DQ055317, DQ055318, DQ055318, DQ055319, DQ055319, DQ055320, DQ055320, DQ055321, DQ055321, DQ055322, DQ055322, DQ055323, DQ055323, DQ055324, DQ055324, DQ055325, DQ055325, DQ055326, DQ055326, DQ055327, DQ055327, DQ055328, DQ055328, DQ055329, DQ055329, DQ055330, DQ055330, DQ055331, DQ055331, DQ055332, DQ055332, DQ055333, DQ055333, DQ055334, DQ055334, DQ055335, DQ055335, DQ055336, DQ055336, DQ070561, DQ070562, DQ070563, DQ070564, DQ070565, DQ070566, DQ070567, DQ070568, DQ070569, DQ070570, DQ070571, DQ070572, DQ070573, DQ070574, DQ070575, DQ070576, DQ070577, DQ070578, DQ070579, DQ070580, DQ070581, DQ070582, DQ070583, DQ070584, DQ070585, DQ070587, DQ070588, DQ070593, DQ070594, DQ070595, DQ070596, DQ070597, DQ070598, DQ070625, DQ070626, DQ070627, DQ070628, DQ070629, DQ070630, DQ070631, DQ070632, DQ070633, DQ070634, DQ070635, DQ070636, DQ070637, DQ070638, DQ070639, DQ070640, DQ070641, DQ070642, DQ070643, DQ070644, DQ070645, DQ070646, DQ070647, DQ070648, DQ070649, DQ070650, DQ070651, DQ070652, DQ070653, DQ070654, DQ070655, DQ070713, DQ070714, DQ070715, DQ070719, DQ108337, DQ108337, DQ108340, DQ108340, DQ108343, DQ108343, DQ108346, DQ108346, DQ108349, DQ108349, DQ108352, DQ108352, DQ108354, DQ108354, DQ108357, DQ108357, DQ108359, DQ108359, DQ108362, DQ108362, DQ108364, DQ108364, DQ108367, DQ108370, DQ108370, DQ108373, DQ108373, DQ108376, DQ108376, DQ108379, DQ108379, DQ108381, DQ108381, DQ199574, DQ199574, DQ199575, DQ199575, DQ199576, DQ199576, DQ199577, DQ199577, DQ199578, DQ199578, DQ199579, DQ199579, DQ199580, DQ199580, DQ199582, DQ199582, DQ199583, DQ199583, DQ199584, DQ199584, DQ199585, DQ199585, DQ199586, DQ199586, DQ199587, DQ199587, DQ199588, DQ199588, DQ199589, DQ199589, DQ199590, DQ199590, DQ199591, DQ199591, DQ199592, DQ199592, DQ199593, DQ199593, DQ199594, DQ199594, DQ199595, DQ199595, DQ199596, DQ199596, DQ199597, DQ199597, DQ199598, DQ199598, DQ199599, DQ199599, DQ199600, DQ199600, DQ199601, DQ199601, DQ199602, DQ199602, DQ199603, DQ199603, DQ199604, DQ199604, DQ199605, DQ199605, DQ199606, DQ199606, DQ199607, DQ199607, DQ199608, DQ199608, DQ199609, DQ199609, DQ199610, DQ199610, DQ199611, DQ199611, DQ199612, DQ199612, DQ199613, DQ199613, DQ199614, DQ199614, DQ205252, DQ205252, DQ205253, DQ205253, DQ205260, DQ205260, DQ205261, DQ205261, DQ205264, DQ205264, DQ205265, DQ205265, DQ205269, DQ205269, DQ205272, DQ205272, DQ205273, DQ205273, DQ205280, DQ205280, DQ205282, DQ205282, DQ205284, DQ205284, DQ206645, DQ206646, DQ206647, DQ206648, DQ206649, DQ206650, DQ206651, DQ206652, DQ206653, DQ206654, DQ206654, DQ206655, DQ206656, DQ206657, DQ206658, DQ206659, DQ206660, DQ206660, DQ206661, DQ206662, DQ206662, DQ206663, DQ206664, DQ206664, DQ206665, DQ206665, DQ206666, DQ206666, DQ206667, DQ206667, DQ206668, DQ206668, DQ206669, DQ206669, DQ206670, DQ206670, DQ206671, DQ206671, DQ206672, DQ206672, DQ206673, DQ206673, DQ206674, DQ206674, DQ273923, DQ286511, DQ286514, DQ286515, DQ286516, DQ286517, DQ286520, DQ286521, DQ286522, DQ328525, DQ328525, DQ328544, DQ328544, DQ328547, DQ328547, DQ328559, DQ345012, DQ345023, DQ345024, DQ345025, DQ345026, DQ345032, DQ345041, DQ345053, DQ345054, DQ345055, DQ345059, DQ345061, DQ345062, DQ345065, DQ345494, DQ345495, DQ345496, DQ345497, DQ345498, DQ345498, DQ345499, DQ345500, DQ345501, DQ345502, DQ345503, DQ345504, DQ345504, DQ345505, DQ345506, DQ345506, DQ345507, DQ345507, DQ345508, DQ345508, DQ345509, DQ345509, DQ345510, DQ345510, DQ345511, DQ345511, DQ345512, DQ345512, DQ345513, DQ345513, DQ345514, DQ345514, DQ345515, DQ345515, DQ396398, DQ396398, DQ443557, DQ443559, DQ443612, DQ443616, DQ443676, K02007, K02007, K02013, K02013, L07423, L08463, L08464, L39021, L39022, L39023, L39024, L39026, L39027, L39028, L39029, L39030, L39031, L39032, L39033, L39034, L39036, L39039, L39041, L39042, L39043, L39045, L39046, L39047, L39048, M17449, M17449, M17451, M17451, M19921, M19921, M26727, M26727, M38429, M38429, M92995, M92996, M92997, M92998, M92999, M93000, M93001, M93002, M93003, M93258, M93258, M93372, M93375, M93379, M93382, M96155, M96155, U12738, U12745, U12746, U12747, U12748, U12749, U12750, U12751, U12752, U12753, U12754, U12755, U12756, U16765, U16767, U16769, U16771, U16773, U16775, U16777, U16779, U19411, U19412, U19413, U19414, U19415, U19417, U19418, U19419, U19420, U19421, U19422, U19423, U19424, U19425, U19426, U19427, U19428, U19429, U19430, U19431, U19441, U19446, U19447, U19448, U19449, U19457, U19458, U21122, U21135, U21135, U21136, U26546, U28648, U31385, U31386, U31387, U31388, U31389, U31390, U31391, U31392, U31393, U31394, U31395, U31399, U31400, U31401, U31402, U31403, U31404, U31405, U31406, U31408, U31412, U37270, U37270, U43096, U43096, U43141, U43141, U71182, U71182, Y10107, Z26585, Z26587, Z26589, Z27232, Z31354, Z31355, Z37142, Z99299, Z99301, Z99303, Z99305, Z99307, Z99311, Z99313, Z99317, Z99319, Z99321, Z99323, Z99325, Z99326, Z99328, Z99330, Z99333, Z99335, Z99336
