# Supplementary material for: HIV-1 Subtype B Protease and Reverse Transcriptase Amino Acid Covariation
Source: PLoS Comput Biol. 2007 May 11;3(5):e87. doi: 10.1371/journal.pcbi.0030087 (PMC1866358; doi:10.1371/journal.pcbi.0030087)
Supplement: Supplementary file 4 [file pcbi.0030087.st001.doc]

**Table S1. 161 Highly Correlated Protease Mutation Pairs from PI Experienced Persons^*^**

| **Mut X** | **Mut Y** | **Association^†^** | | | | | **P(Y\|X)** | **P(X\|Y)** |
| --- | --- | --- | --- | --- | --- | --- | --- | --- |
|  |  | **J** | **J_RAND_** | **J_SE_** | **Z** | **p** |  |  |
| *Positive correlations* | | | | | | | | |
| 54V | 82A | 0.51 | 0.11 | 0.02 | 23.7 | 0 | 0.73 | 0.63 |
| 30N | 88D | 0.59 | 0.04 | 0.03 | 21.0 | 0 | 0.62 | 0.91 |
| 35D | 36I | 0.40 | 0.15 | 0.01 | 17.8 | 0 | 0.55 | 0.60 |
| 71V | 90M | 0.38 | 0.17 | 0.01 | 15.8 | 0 | 0.59 | 0.51 |
| 10I | 54V | 0.34 | 0.14 | 0.01 | 14.8 | 0 | 0.39 | 0.69 |
| 10I | 90M | 0.38 | 0.19 | 0.01 | 14.7 | 0 | 0.54 | 0.56 |
| 10I | 82A | 0.34 | 0.15 | 0.01 | 14.5 | 0 | 0.42 | 0.65 |
| 71V | 82A | 0.34 | 0.14 | 0.01 | 14.4 | 0 | 0.45 | 0.57 |
| 54V | 71V | 0.32 | 0.13 | 0.01 | 14.2 | 0 | 0.60 | 0.41 |
| 10I | 71V | 0.36 | 0.18 | 0.01 | 14.1 | 0 | 0.48 | 0.58 |
| 20R | 36I | 0.29 | 0.07 | 0.02 | 14.1 | 0 | 0.89 | 0.30 |
| 77I | 93L | 0.34 | 0.20 | 0.01 | 11.8 | 0 | 0.54 | 0.47 |
| 46L | 82A | 0.25 | 0.07 | 0.02 | 11.7 | 0 | 0.68 | 0.28 |
| 73S | 90M | 0.21 | 0.07 | 0.01 | 11.5 | 0 | 0.87 | 0.22 |
| 36I | 62V | 0.30 | 0.16 | 0.01 | 11.3 | 0 | 0.50 | 0.43 |
| 84V | 90M | 0.23 | 0.09 | 0.01 | 11.2 | 0 | 0.70 | 0.26 |
| 46I | 90M | 0.26 | 0.13 | 0.01 | 10.6 | 0 | 0.57 | 0.33 |
| 10I | 84V | 0.22 | 0.09 | 0.01 | 10.5 | 0 | 0.25 | 0.69 |
| 62V | 90M | 0.30 | 0.18 | 0.01 | 10.1 | 0 | 0.48 | 0.45 |
| 71V | 73S | 0.19 | 0.06 | 0.01 | 10.0 | 0 | 0.21 | 0.73 |
| 20R | 54V | 0.20 | 0.06 | 0.01 | 9.5 | 0 | 0.54 | 0.24 |
| 63P | 90M | 0.38 | 0.29 | 0.01 | 9.5 | 0 | 0.40 | 0.89 |
| 54V | 90M | 0.24 | 0.14 | 0.01 | 9.3 | 0 | 0.53 | 0.31 |
| 46L | 54V | 0.20 | 0.06 | 0.01 | 9.2 | 0 | 0.53 | 0.24 |
| 90M | 93L | 0.31 | 0.20 | 0.01 | 9.2 | 0 | 0.49 | 0.45 |
| 24I | 54V | 0.17 | 0.04 | 0.01 | 9.0 | 0 | 0.71 | 0.18 |
| 36I | 54V | 0.23 | 0.12 | 0.01 | 9.0 | 0 | 0.32 | 0.45 |
| 24I | 82A | 0.16 | 0.04 | 0.01 | 8.9 | 0 | 0.75 | 0.17 |
| 10I | 46I | 0.24 | 0.13 | 0.01 | 8.8 | 0 | 0.30 | 0.53 |
| 20R | 35D | 0.17 | 0.07 | 0.01 | 8.8 | 0 | 0.64 | 0.19 |
| 46I | 84V | 0.20 | 0.08 | 0.01 | 8.8 | 0 | 0.27 | 0.43 |
| 71V | 84V | 0.19 | 0.09 | 0.01 | 8.3 | 0 | 0.22 | 0.54 |
| 20R | 82A | 0.17 | 0.06 | 0.01 | 8.3 | 0 | 0.53 | 0.20 |
| 10I | 93L | 0.30 | 0.20 | 0.01 | 8.2 | 2.22E-16 | 0.47 | 0.44 |
| 10I | 62V | 0.28 | 0.18 | 0.01 | 8.0 | 1.11E-15 | 0.41 | 0.46 |
| 35D | 37D | 0.19 | 0.10 | 0.01 | 7.9 | 2.22E-15 | 0.24 | 0.49 |
| 48V | 82A | 0.13 | 0.03 | 0.01 | 7.8 | 6.00E-15 | 0.77 | 0.13 |
| 20I | 90M | 0.13 | 0.05 | 0.01 | 7.7 | 1.13E-14 | 0.77 | 0.13 |
| 24I | 46L | 0.18 | 0.03 | 0.02 | 7.5 | 5.62E-14 | 0.43 | 0.24 |
| 62V | 93L | 0.28 | 0.19 | 0.01 | 7.5 | 7.06E-14 | 0.47 | 0.40 |
| 73S | 84V | 0.17 | 0.05 | 0.02 | 7.4 | 1.25E-13 | 0.36 | 0.24 |
| 32I | 46I | 0.13 | 0.03 | 0.01 | 7.2 | 4.28E-13 | 0.64 | 0.14 |
| 32I | 82A | 0.12 | 0.04 | 0.01 | 7.2 | 7.66E-13 | 0.67 | 0.13 |
| 32I | 47V | 0.27 | 0.01 | 0.04 | 7.2 | 8.21E-13 | 0.30 | 0.76 |
| 36I | 90M | 0.24 | 0.17 | 0.01 | 7.1 | 1.59E-12 | 0.44 | 0.35 |
| 12P | 14R | 0.18 | 0.02 | 0.02 | 7.1 | 1.70E-12 | 0.63 | 0.20 |
| 10I | 48V | 0.10 | 0.03 | 0.01 | 7.0 | 2.21E-12 | 0.10 | 0.88 |
| 20I | 46I | 0.13 | 0.04 | 0.01 | 6.9 | 4.98E-12 | 0.52 | 0.15 |
| 10I | 73S | 0.13 | 0.06 | 0.01 | 6.9 | 5.73E-12 | 0.15 | 0.64 |
| 13V | 30N | 0.16 | 0.07 | 0.01 | 6.8 | 8.03E-12 | 0.21 | 0.39 |
| 46L | 71V | 0.15 | 0.07 | 0.01 | 6.8 | 1.18E-11 | 0.53 | 0.17 |
| 36I | 71V | 0.23 | 0.15 | 0.01 | 6.7 | 1.82E-11 | 0.39 | 0.36 |
| 43T | 82A | 0.10 | 0.03 | 0.01 | 6.6 | 4.77E-11 | 0.77 | 0.10 |
| 46I | 93L | 0.21 | 0.14 | 0.01 | 6.5 | 7.66E-11 | 0.50 | 0.26 |
| 10I | 20R | 0.13 | 0.07 | 0.01 | 6.3 | 2.64E-10 | 0.15 | 0.59 |
| 10I | 24I | 0.10 | 0.04 | 0.01 | 6.3 | 2.77E-10 | 0.11 | 0.72 |
| 63P | 93L | 0.38 | 0.32 | 0.01 | 6.3 | 3.15E-10 | 0.41 | 0.83 |
| 13V | 88D | 0.13 | 0.05 | 0.01 | 6.3 | 3.83E-10 | 0.15 | 0.44 |
| 20I | 84V | 0.14 | 0.04 | 0.02 | 6.2 | 4.16E-10 | 0.37 | 0.18 |
| 62V | 73S | 0.13 | 0.06 | 0.01 | 6.2 | 4.78E-10 | 0.14 | 0.56 |
| 20R | 71V | 0.13 | 0.07 | 0.01 | 6.1 | 1.28E-09 | 0.51 | 0.15 |
| 20R | 62V | 0.13 | 0.07 | 0.01 | 6.0 | 2.22E-09 | 0.53 | 0.15 |
| 10I | 46L | 0.13 | 0.07 | 0.01 | 6.0 | 2.42E-09 | 0.15 | 0.56 |
| 53L | 71V | 0.08 | 0.03 | 0.01 | 5.9 | 3.31E-09 | 0.75 | 0.08 |
| 32I | 71V | 0.09 | 0.04 | 0.01 | 5.9 | 3.82E-09 | 0.65 | 0.10 |
| 36I | 82A | 0.20 | 0.13 | 0.01 | 5.8 | 7.52E-09 | 0.31 | 0.36 |
| 46I | 71V | 0.19 | 0.13 | 0.01 | 5.7 | 1.20E-08 | 0.39 | 0.27 |
| 82A | 90M | 0.21 | 0.15 | 0.01 | 5.7 | 1.24E-08 | 0.43 | 0.29 |
| 15V | 36I | 0.18 | 0.11 | 0.01 | 5.7 | 1.32E-08 | 0.38 | 0.25 |
| 13V | 33F | 0.10 | 0.04 | 0.01 | 5.7 | 1.51E-08 | 0.11 | 0.49 |
| 54V | 62V | 0.19 | 0.13 | 0.01 | 5.6 | 1.94E-08 | 0.41 | 0.26 |
| 53L | 54V | 0.09 | 0.03 | 0.01 | 5.5 | 3.27E-08 | 0.57 | 0.09 |
| 35D | 37E | 0.08 | 0.03 | 0.01 | 5.5 | 3.36E-08 | 0.08 | 0.72 |
| 60E | 61E | 0.12 | 0.02 | 0.02 | 5.5 | 3.49E-08 | 0.15 | 0.40 |
| 33F | 84V | 0.11 | 0.03 | 0.01 | 5.5 | 4.11E-08 | 0.36 | 0.14 |
| 10I | 36I | 0.23 | 0.17 | 0.01 | 5.5 | 4.40E-08 | 0.33 | 0.43 |
| 63P | 71V | 0.30 | 0.25 | 0.01 | 5.5 | 4.74E-08 | 0.32 | 0.83 |
| 43T | 54V | 0.08 | 0.03 | 0.01 | 5.4 | 5.95E-08 | 0.59 | 0.09 |
| 53L | 82A | 0.08 | 0.03 | 0.01 | 5.4 | 6.79E-08 | 0.60 | 0.09 |
| 46I | 55R | 0.08 | 0.02 | 0.01 | 5.4 | 7.09E-08 | 0.09 | 0.58 |
| 20R | 90M | 0.12 | 0.07 | 0.01 | 5.4 | 7.54E-08 | 0.53 | 0.14 |
| 20I | 73S | 0.12 | 0.03 | 0.02 | 5.4 | 7.93E-08 | 0.27 | 0.19 |
| 48V | 54T | 0.20 | 0.01 | 0.04 | 5.3 | 8.84E-08 | 0.21 | 0.86 |
| 72L | 73S | 0.12 | 0.01 | 0.02 | 5.3 | 9.12E-08 | 0.69 | 0.13 |
| 71V | 93L | 0.24 | 0.18 | 0.01 | 5.3 | 9.66E-08 | 0.44 | 0.34 |
| 35D | 63P | 0.31 | 0.26 | 0.01 | 5.3 | 1.38E-07 | 0.83 | 0.33 |
| 54V | 58E | 0.09 | 0.03 | 0.01 | 5.2 | 1.57E-07 | 0.09 | 0.51 |
| 73T | 90M | 0.05 | 0.02 | 0.01 | 5.1 | 3.15E-07 | 0.93 | 0.05 |
| 62V | 71V | 0.22 | 0.17 | 0.01 | 5.1 | 3.25E-07 | 0.35 | 0.38 |
| 35D | 62V | 0.22 | 0.17 | 0.01 | 5.1 | 3.26E-07 | 0.38 | 0.35 |
| 71T | 72T | 0.10 | 0.02 | 0.02 | 5.1 | 4.31E-07 | 0.12 | 0.41 |
| 33F | 82A | 0.09 | 0.04 | 0.01 | 5.1 | 4.35E-07 | 0.48 | 0.10 |
| *Negative correlations* | | | | | | | | |
| 30N | 82A | 0.00 | 0.08 | 0.00 | -82.5 | 0 | 0.00 | 0.00 |
| 82A | 88D | 0.00 | 0.06 | 0.00 | -53.5 | 0 | 0.00 | 0.00 |
| 24I | 90M | 0.00 | 0.04 | 0.00 | -37.7 | 0 | 0.01 | 0.00 |
| 36I | 77I | 0.03 | 0.17 | 0.00 | -31.9 | 0 | 0.07 | 0.06 |
| 30N | 73S | 0.00 | 0.05 | 0.00 | -27.4 | 0 | 0.00 | 0.00 |
| 30N | 54V | 0.01 | 0.07 | 0.00 | -22.9 | 0 | 0.02 | 0.01 |
| 30N | 90M | 0.02 | 0.09 | 0.00 | -18.4 | 0 | 0.08 | 0.02 |
| 73S | 88D | 0.00 | 0.04 | 0.00 | -17.9 | 0 | 0.00 | 0.00 |
| 16E | 63P | 0.00 | 0.02 | 0.00 | -17.3 | 0 | 0.11 | 0.00 |
| 64V | 73S | 0.01 | 0.06 | 0.00 | -15.4 | 0 | 0.01 | 0.03 |
| 10I | 30N | 0.03 | 0.09 | 0.00 | -14.6 | 0 | 0.03 | 0.10 |
| 15V | 77I | 0.05 | 0.13 | 0.01 | -14.0 | 0 | 0.13 | 0.07 |
| 30N | 84V | 0.01 | 0.06 | 0.00 | -11.4 | 0 | 0.03 | 0.02 |
| 64V | 71V | 0.06 | 0.13 | 0.01 | -11.3 | 0 | 0.13 | 0.10 |
| 66F | 93L | 0.00 | 0.01 | 0.00 | -11.3 | 0 | 0.03 | 0.00 |
| 20M | 77I | 0.00 | 0.02 | 0.00 | -11.3 | 0 | 0.04 | 0.00 |
| 35D | 77I | 0.10 | 0.18 | 0.01 | -11.2 | 0 | 0.18 | 0.17 |
| 33V | 77I | 0.00 | 0.01 | 0.00 | -11.2 | 0 | 0.03 | 0.00 |
| 63P | 64V | 0.11 | 0.18 | 0.01 | -11.1 | 0 | 0.13 | 0.48 |
| 54V | 88D | 0.01 | 0.05 | 0.00 | -10.9 | 0 | 0.02 | 0.05 |
| 64V | 71T | 0.02 | 0.07 | 0.00 | -10.7 | 0 | 0.03 | 0.06 |
| 13V | 93L | 0.08 | 0.14 | 0.01 | -10.1 | 0 | 0.20 | 0.11 |
| 20R | 77I | 0.03 | 0.07 | 0.00 | -9.4 | 0 | 0.13 | 0.03 |
| 32I | 88D | 0.00 | 0.03 | 0.00 | -9.2 | 0 | 0.01 | 0.00 |
| 69N | 93L | 0.00 | 0.01 | 0.00 | -9.1 | 0 | 0.04 | 0.00 |
| 30N | 46I | 0.03 | 0.07 | 0.01 | -8.7 | 0 | 0.07 | 0.04 |
| 30N | 46L | 0.01 | 0.05 | 0.00 | -8.7 | 0 | 0.02 | 0.03 |
| 10I | 88D | 0.02 | 0.06 | 0.00 | -8.5 | 0 | 0.03 | 0.14 |
| 37D | 41K | 0.04 | 0.09 | 0.01 | -8.1 | 4.44E-16 | 0.09 | 0.06 |
| 69Q | 93L | 0.01 | 0.02 | 0.00 | -8.0 | 1.78E-15 | 0.09 | 0.01 |
| 64V | 72T | 0.01 | 0.03 | 0.00 | -7.7 | 9.55E-15 | 0.01 | 0.04 |
| 63T | 90M | 0.01 | 0.02 | 0.00 | -7.7 | 1.24E-14 | 0.08 | 0.01 |
| 82A | 88S | 0.00 | 0.02 | 0.00 | -7.6 | 2.02E-14 | 0.00 | 0.03 |
| 63S | 84V | 0.00 | 0.03 | 0.00 | -7.6 | 2.66E-14 | 0.02 | 0.01 |
| 63A | 73S | 0.00 | 0.02 | 0.00 | -7.6 | 2.86E-14 | 0.01 | 0.00 |
| 12S | 37D | 0.00 | 0.02 | 0.00 | -7.3 | 2.43E-13 | 0.02 | 0.00 |
| 33V | 82A | 0.00 | 0.01 | 0.00 | -7.2 | 5.07E-13 | 0.03 | 0.00 |
| 24I | 30N | 0.00 | 0.03 | 0.01 | -6.8 | 9.08E-12 | 0.00 | 0.00 |
| 30N | 32I | 0.00 | 0.03 | 0.00 | -6.7 | 2.58E-11 | 0.00 | 0.00 |
| 20I | 32I | 0.00 | 0.02 | 0.00 | -6.6 | 3.97E-11 | 0.01 | 0.01 |
| 20I | 30N | 0.01 | 0.04 | 0.00 | -6.4 | 1.19E-10 | 0.03 | 0.01 |
| 33V | 54V | 0.00 | 0.01 | 0.00 | -6.4 | 1.31E-10 | 0.03 | 0.00 |
| 30N | 48V | 0.00 | 0.03 | 0.00 | -6.4 | 1.35E-10 | 0.00 | 0.00 |
| 54V | 88S | 0.00 | 0.02 | 0.00 | -6.4 | 1.39E-10 | 0.00 | 0.03 |
| 73S | 74S | 0.01 | 0.03 | 0.00 | -6.3 | 2.29E-10 | 0.01 | 0.01 |
| 46L | 88D | 0.01 | 0.04 | 0.00 | -6.3 | 3.84E-10 | 0.02 | 0.03 |
| 24I | 88D | 0.00 | 0.03 | 0.00 | -6.2 | 7.04E-10 | 0.00 | 0.00 |
| 88D | 90M | 0.03 | 0.06 | 0.00 | -6.2 | 7.40E-10 | 0.17 | 0.04 |
| 54V | 77I | 0.09 | 0.14 | 0.01 | -6.2 | 7.45E-10 | 0.22 | 0.13 |
| 13V | 82A | 0.07 | 0.11 | 0.01 | -6.1 | 8.27E-10 | 0.14 | 0.12 |
| 71V | 88S | 0.00 | 0.02 | 0.00 | -6.0 | 1.46E-09 | 0.00 | 0.07 |
| 65D | 93L | 0.00 | 0.01 | 0.00 | -6.0 | 2.41E-09 | 0.09 | 0.00 |
| 30N | 43T | 0.00 | 0.02 | 0.00 | -6.0 | 2.42E-09 | 0.01 | 0.02 |
| 20I | 82A | 0.02 | 0.05 | 0.00 | -5.9 | 3.64E-09 | 0.09 | 0.02 |
| 64V | 71I | 0.00 | 0.02 | 0.00 | -5.8 | 7.78E-09 | 0.00 | 0.05 |
| 55R | 88D | 0.00 | 0.02 | 0.00 | -5.7 | 9.34E-09 | 0.01 | 0.00 |
| 77I | 82A | 0.10 | 0.15 | 0.01 | -5.7 | 1.48E-08 | 0.16 | 0.23 |
| 69N | 82A | 0.00 | 0.01 | 0.00 | -5.6 | 2.15E-08 | 0.03 | 0.00 |
| 54V | 63S | 0.01 | 0.03 | 0.00 | -5.6 | 2.21E-08 | 0.01 | 0.06 |
| 64V | 93L | 0.10 | 0.14 | 0.01 | -5.6 | 2.79E-08 | 0.26 | 0.14 |
| 63S | 73S | 0.00 | 0.02 | 0.00 | -5.5 | 2.92E-08 | 0.00 | 0.00 |
| 48V | 73S | 0.00 | 0.03 | 0.00 | -5.5 | 3.34E-08 | 0.00 | 0.00 |
| 63Q | 84V | 0.00 | 0.01 | 0.00 | -5.4 | 6.29E-08 | 0.02 | 0.00 |
| 37T | 82A | 0.01 | 0.02 | 0.00 | -5.2 | 1.70E-07 | 0.07 | 0.01 |
| 37E | 41K | 0.01 | 0.03 | 0.00 | -5.2 | 1.87E-07 | 0.06 | 0.01 |
| 30N | 82T | 0.00 | 0.02 | 0.00 | -5.2 | 2.16E-07 | 0.00 | 0.00 |
| 64V | 69K | 0.01 | 0.02 | 0.00 | -5.2 | 2.46E-07 | 0.01 | 0.06 |
| 30N | 55R | 0.00 | 0.02 | 0.00 | -5.1 | 3.41E-07 | 0.00 | 0.00 |
| 20T | 82A | 0.01 | 0.02 | 0.00 | -5.0 | 4.79E-07 | 0.07 | 0.01 |

**^*^** 161 pairs from PI experienced persons were significantly associated after adjusting for multiple comparisons by controlling the family-wise error rate at < 0.01

**^†^**J: Jaccard similarity coefficients; J_RAND_: expected value of the Jaccard similarity coefficient assuming Mut X and Mut Y occur independently based on a permutation procedure described in the methods; J_SE_ : the standard error of J; Z: (J- J_RAND_)/ J_SE_. Mutation pairs are ordered by their Z statistic.
