# Supplementary material for: HIV-1 Subtype B Protease and Reverse Transcriptase Amino Acid Covariation
Source: PLoS Comput Biol. 2007 May 11;3(5):e87. doi: 10.1371/journal.pcbi.0030087 (PMC1866358; doi:10.1371/journal.pcbi.0030087)
Supplement: Supplementary file 5 [file pcbi.0030087.st002.doc]

**Table S2. Highly Correlated HIV-1 Subtype B Protease Mutation Pairs from PI-Naive Persons**

| **Mut X** | **Mut Y** | **Association^†^** | | | | | **P(Y\|X)** | **P(X\|Y)** |
| --- | --- | --- | --- | --- | --- | --- | --- | --- |
|  |  | **J** | **J_RAND_** | **J_SE_** | **Z** | **p** |  |  |
| *Positive correlations* | | | | | | | | |
| 77I | 93L | 0.32 | 0.13 | 0.01 | 14.28 | 0.00 | 0.48 | 0.48 |
| 62V | 93L | 0.21 | 0.12 | 0.01 | 8.34 | 0.00 | 0.39 | 0.32 |
| 33V | 37Y | 0.34 | 0.01 | 0.04 | 7.65 | 0.00 | 0.36 | 0.87 |
| 63P | 93L | 0.27 | 0.20 | 0.01 | 7.36 | 0.00 | 0.31 | 0.70 |
| 35D | 63P | 0.27 | 0.20 | 0.01 | 7.28 | 0.00 | 0.69 | 0.31 |
| 37Y | 69Q | 0.26 | 0.01 | 0.04 | 7.06 | 0.00 | 0.85 | 0.28 |
| 37Y | 64L | 0.29 | 0.01 | 0.04 | 6.96 | 0.00 | 0.79 | 0.32 |
| 71V | 93L | 0.09 | 0.02 | 0.01 | 6.94 | 0.00 | 0.85 | 0.09 |
| 35D | 37D | 0.14 | 0.07 | 0.01 | 6.92 | 0.00 | 0.17 | 0.46 |
| 33V | 64L | 0.21 | 0.02 | 0.03 | 6.64 | 0.00 | 0.35 | 0.34 |
| 71T | 93L | 0.10 | 0.04 | 0.01 | 6.55 | 0.00 | 0.58 | 0.11 |
| 33V | 69Q | 0.18 | 0.02 | 0.03 | 6.48 | 0.00 | 0.35 | 0.28 |
| 60E | 61E | 0.16 | 0.02 | 0.02 | 6.39 | 0.00 | 0.19 | 0.49 |
| 64L | 69Q | 0.18 | 0.02 | 0.03 | 6.37 | 0.00 | 0.34 | 0.28 |
| 72V | 77I | 0.12 | 0.06 | 0.01 | 6.16 | 0.00 | 0.47 | 0.14 |
| 36I | 57K | 0.13 | 0.06 | 0.01 | 5.84 | 0.00 | 0.20 | 0.27 |
| 36I | 62V | 0.14 | 0.08 | 0.01 | 5.39 | 0.00 | 0.31 | 0.21 |
| 37Y | 62V | 0.06 | 0.01 | 0.01 | 5.38 | 0.00 | 0.91 | 0.06 |
| 71T | 77I | 0.09 | 0.04 | 0.01 | 5.30 | 0.00 | 0.49 | 0.10 |
| 62V | 64L | 0.08 | 0.03 | 0.01 | 5.23 | 0.00 | 0.08 | 0.52 |
| 15V | 36I | 0.13 | 0.07 | 0.01 | 5.19 | 0.00 | 0.22 | 0.25 |
| 37S | 41K | 0.15 | 0.10 | 0.01 | 5.07 | 0.00 | 0.32 | 0.23 |
| 36I | 41K | 0.14 | 0.09 | 0.01 | 4.85 | 0.00 | 0.32 | 0.19 |
| 41K | 93L | 0.18 | 0.13 | 0.01 | 4.85 | 0.00 | 0.32 | 0.29 |
| 57K | 61N | 0.07 | 0.01 | 0.01 | 4.85 | 0.00 | 0.08 | 0.74 |
| 41K | 60E | 0.09 | 0.05 | 0.01 | 4.83 | 0.00 | 0.10 | 0.41 |
| *Negative correlations* | | | | | | | | |
| 93L | 95S | 0.00 | 0.03 | 0.00 | -26.74 | 0.00 | 0.00 | 0.01 |
| 33V | 77I | 0.00 | 0.03 | 0.00 | -19.48 | 0.00 | 0.02 | 0.00 |
| 63P | 64V | 0.06 | 0.15 | 0.00 | -18.20 | 0.00 | 0.07 | 0.24 |
| 36I | 77I | 0.02 | 0.09 | 0.00 | -16.87 | 0.00 | 0.07 | 0.04 |
| 64V | 71T | 0.00 | 0.04 | 0.00 | -16.50 | 0.00 | 0.00 | 0.02 |
| 64V | 71V | 0.00 | 0.02 | 0.00 | -15.19 | 0.00 | 0.00 | 0.01 |
| 64V | 93L | 0.04 | 0.11 | 0.01 | -12.08 | 0.00 | 0.10 | 0.07 |
| 15V | 77I | 0.04 | 0.10 | 0.01 | -11.33 | 0.00 | 0.10 | 0.06 |
| 15V | 64L | 0.00 | 0.03 | 0.00 | -10.66 | 0.00 | 0.00 | 0.02 |
| 69N | 93L | 0.00 | 0.01 | 0.00 | -10.50 | 0.00 | 0.02 | 0.00 |
| 37E | 41K | 0.00 | 0.03 | 0.00 | -10.41 | 0.00 | 0.04 | 0.00 |
| 69Q | 93L | 0.01 | 0.03 | 0.00 | -9.83 | 0.00 | 0.06 | 0.01 |
| 64V | 72T | 0.00 | 0.02 | 0.00 | -9.16 | 0.00 | 0.00 | 0.02 |
| 63A | 64V | 0.01 | 0.03 | 0.00 | -8.42 | 0.00 | 0.04 | 0.01 |
| 62V | 64V | 0.05 | 0.10 | 0.01 | -8.28 | 0.00 | 0.09 | 0.10 |
| 64V | 77I | 0.06 | 0.11 | 0.01 | -7.96 | 0.00 | 0.14 | 0.10 |
| 64V | 89M | 0.00 | 0.01 | 0.00 | -7.44 | 0.00 | 0.00 | 0.02 |
| 33V | 35D | 0.01 | 0.03 | 0.00 | -7.41 | 0.00 | 0.07 | 0.01 |
| 16E | 63P | 0.01 | 0.03 | 0.00 | -7.30 | 0.00 | 0.21 | 0.01 |
| 15V | 33V | 0.00 | 0.03 | 0.00 | -7.21 | 0.00 | 0.00 | 0.00 |
| 33V | 72V | 0.00 | 0.02 | 0.00 | -7.13 | 0.00 | 0.01 | 0.00 |
| 37Y | 64V | 0.00 | 0.01 | 0.00 | -7.00 | 0.00 | 0.02 | 0.00 |
| 61N | 63P | 0.00 | 0.01 | 0.00 | -6.92 | 0.00 | 0.14 | 0.00 |
| 37D | 41K | 0.03 | 0.07 | 0.01 | -6.83 | 0.00 | 0.10 | 0.04 |
| 36I | 37S | 0.04 | 0.08 | 0.01 | -6.66 | 0.00 | 0.08 | 0.07 |
| 36I | 37C | 0.00 | 0.01 | 0.00 | -6.62 | 0.00 | 0.00 | 0.02 |
| 37Y | 77I | 0.00 | 0.01 | 0.00 | -6.59 | 0.00 | 0.04 | 0.00 |
| 61N | 62V | 0.00 | 0.01 | 0.00 | -6.13 | 0.00 | 0.03 | 0.00 |
| 41K | 69N | 0.00 | 0.01 | 0.00 | -6.08 | 0.00 | 0.00 | 0.04 |
| 33V | 93L | 0.01 | 0.03 | 0.00 | -6.04 | 0.00 | 0.08 | 0.01 |
| 15V | 63P | 0.09 | 0.13 | 0.01 | -5.93 | 0.00 | 0.41 | 0.11 |
| 37Y | 41K | 0.00 | 0.01 | 0.00 | -5.92 | 0.00 | 0.04 | 0.00 |
| 35D | 37C | 0.00 | 0.01 | 0.00 | -5.77 | 0.00 | 0.00 | 0.05 |
| 64V | 69Q | 0.01 | 0.03 | 0.00 | -5.45 | 0.00 | 0.01 | 0.06 |
| 61N | 64V | 0.00 | 0.01 | 0.00 | -5.23 | 0.00 | 0.03 | 0.00 |
| 57K | 63Q | 0.00 | 0.01 | 0.00 | -4.97 | 0.00 | 0.00 | 0.02 |
| 63H | 93L | 0.01 | 0.02 | 0.00 | -4.93 | 0.00 | 0.08 | 0.01 |
| 15V | 71V | 0.01 | 0.02 | 0.00 | -4.89 | 0.00 | 0.01 | 0.04 |
| 15V | 93L | 0.07 | 0.10 | 0.01 | -4.87 | 0.00 | 0.17 | 0.10 |

**^†^**J: Jaccard similarity coefficients; J_RAND_: expected value of the Jaccard similarity coefficient assuming Mut X and Mut Y occur independently based on a permutation procedure described in the methods; J_SE_ : the standard error of J; Z: (J- J_RAND_)/ J_SE_. Mutation pairs are ordered by their Z statistic.
