# Supplementary material for: HIV-1 Subtype B Protease and Reverse Transcriptase Amino Acid Covariation
Source: PLoS Comput Biol. 2007 May 11;3(5):e87. doi: 10.1371/journal.pcbi.0030087 (PMC1866358; doi:10.1371/journal.pcbi.0030087)
Supplement: Supplementary file 6 [file pcbi.0030087.st003.doc]

**Table S3. 327 Highly Correlated Protease Mutation Pairs from RTI-Experienced Persons^*^**

| **Mut X** | **Mut Y** | **Association^†^** | | | | | **P(Y\|X)** | **P(X\|Y)** |
| --- | --- | --- | --- | --- | --- | --- | --- | --- |
|  |  | **J** | **J_RAND_** | **J_SE_** | **Z** | **p** |  |  |
| *Positive correlations* | | | | | | | | |
| 41L | 215Y | 0.73 | 0.24 | 0.01 | 51.9 | 0 | 0.84 | 0.86 |
| 210W | 215Y | 0.60 | 0.17 | 0.01 | 37.2 | 0 | 0.94 | 0.62 |
| 41L | 210W | 0.59 | 0.17 | 0.01 | 36.5 | 0 | 0.61 | 0.94 |
| 70R | 219Q | 0.53 | 0.10 | 0.01 | 29.7 | 0 | 0.56 | 0.90 |
| 67N | 70R | 0.45 | 0.15 | 0.01 | 25.2 | 0 | 0.55 | 0.70 |
| 67N | 219Q | 0.41 | 0.11 | 0.01 | 23.6 | 0 | 0.43 | 0.88 |
| 118I | 210W | 0.39 | 0.10 | 0.01 | 22.0 | 0 | 0.71 | 0.47 |
| 116Y | 151M | 0.73 | 0.01 | 0.04 | 18.5 | 0 | 0.95 | 0.76 |
| 44D | 210W | 0.30 | 0.06 | 0.01 | 17.7 | 0 | 0.92 | 0.31 |
| 215F | 219Q | 0.36 | 0.06 | 0.02 | 17.7 | 0 | 0.65 | 0.44 |
| 41L | 118I | 0.29 | 0.12 | 0.01 | 16.7 | 0 | 0.32 | 0.78 |
| 70R | 215F | 0.29 | 0.07 | 0.01 | 16.6 | 0 | 0.31 | 0.78 |
| 210W | 211K | 0.33 | 0.18 | 0.01 | 15.7 | 0 | 0.73 | 0.38 |
| 44D | 118I | 0.30 | 0.05 | 0.02 | 15.3 | 0 | 0.68 | 0.34 |
| 118I | 215Y | 0.27 | 0.12 | 0.01 | 15.1 | 0 | 0.73 | 0.30 |
| 67N | 118I | 0.27 | 0.11 | 0.01 | 14.9 | 0 | 0.32 | 0.63 |
| 67N | 210W | 0.30 | 0.15 | 0.01 | 14.4 | 0 | 0.41 | 0.52 |
| 41L | 44D | 0.20 | 0.07 | 0.01 | 14.4 | 0 | 0.20 | 0.99 |
| 41L | 67N | 0.34 | 0.20 | 0.01 | 14.4 | 0 | 0.45 | 0.57 |
| 44D | 215Y | 0.21 | 0.07 | 0.01 | 14.3 | 0 | 0.98 | 0.21 |
| 67N | 69D | 0.20 | 0.06 | 0.01 | 13.6 | 0 | 0.21 | 0.85 |
| 67N | 215F | 0.22 | 0.08 | 0.01 | 13.5 | 0 | 0.24 | 0.75 |
| 208Y | 210W | 0.20 | 0.05 | 0.01 | 12.8 | 0 | 0.81 | 0.21 |
| 123E | 177E | 0.25 | 0.12 | 0.01 | 12.7 | 0 | 0.36 | 0.45 |
| 75I | 116Y | 0.57 | 0.01 | 0.04 | 12.6 | 0 | 0.76 | 0.70 |
| 77L | 116Y | 0.58 | 0.01 | 0.05 | 12.5 | 0 | 0.77 | 0.70 |
| 43E | 210W | 0.19 | 0.04 | 0.01 | 12.4 | 0 | 0.86 | 0.19 |
| 77L | 151M | 0.52 | 0.01 | 0.04 | 12.1 | 0 | 0.81 | 0.60 |
| 69N | 70R | 0.18 | 0.04 | 0.01 | 12.0 | 0 | 0.83 | 0.19 |
| 44D | 208Y | 0.26 | 0.03 | 0.02 | 11.7 | 0 | 0.37 | 0.48 |
| 218E | 219Q | 0.21 | 0.04 | 0.01 | 11.7 | 0 | 0.68 | 0.23 |
| 75I | 151M | 0.49 | 0.01 | 0.04 | 11.7 | 0 | 0.78 | 0.57 |
| 75I | 77L | 0.54 | 0.01 | 0.05 | 11.5 | 0 | 0.68 | 0.72 |
| 211K | 215Y | 0.37 | 0.26 | 0.01 | 11.5 | 0 | 0.49 | 0.59 |
| 60I | 67N | 0.21 | 0.09 | 0.01 | 11.4 | 0 | 0.59 | 0.24 |
| 44D | 67N | 0.17 | 0.06 | 0.01 | 11.4 | 0 | 0.72 | 0.19 |
| 118I | 208Y | 0.20 | 0.04 | 0.01 | 11.3 | 0 | 0.23 | 0.58 |
| 41L | 208Y | 0.15 | 0.05 | 0.01 | 11.3 | 0 | 0.15 | 0.94 |
| 67N | 215Y | 0.30 | 0.20 | 0.01 | 11.2 | 0 | 0.53 | 0.41 |
| 214L | 219Q | 0.21 | 0.07 | 0.01 | 11.2 | 0 | 0.37 | 0.33 |
| 70R | 214L | 0.21 | 0.09 | 0.01 | 11.2 | 0 | 0.27 | 0.49 |
| 67N | 208Y | 0.16 | 0.05 | 0.01 | 11.2 | 0 | 0.16 | 0.81 |
| 43E | 118I | 0.19 | 0.04 | 0.01 | 10.7 | 0 | 0.61 | 0.21 |
| 181C | 190A | 0.22 | 0.03 | 0.02 | 10.6 | 0 | 0.29 | 0.51 |
| 41L | 43E | 0.13 | 0.05 | 0.01 | 10.6 | 0 | 0.13 | 0.94 |
| 41L | 211K | 0.36 | 0.26 | 0.01 | 10.5 | 0 | 0.57 | 0.48 |
| 122E | 210W | 0.26 | 0.16 | 0.01 | 10.3 | 0 | 0.35 | 0.49 |
| 39A | 41L | 0.16 | 0.08 | 0.01 | 10.1 | 0 | 0.73 | 0.17 |
| 67N | 218E | 0.13 | 0.04 | 0.01 | 10.1 | 0 | 0.13 | 0.83 |
| 69D | 118I | 0.17 | 0.05 | 0.01 | 9.9 | 0 | 0.46 | 0.22 |
| 70R | 218E | 0.14 | 0.04 | 0.01 | 9.8 | 0 | 0.15 | 0.72 |
| 43E | 215Y | 0.12 | 0.05 | 0.01 | 9.7 | 0 | 0.88 | 0.13 |
| 69N | 219Q | 0.17 | 0.04 | 0.01 | 9.6 | 0 | 0.53 | 0.19 |
| 219Q | 228H | 0.18 | 0.04 | 0.01 | 9.6 | 0 | 0.21 | 0.51 |
| 208Y | 215Y | 0.13 | 0.05 | 0.01 | 9.6 | 0 | 0.82 | 0.13 |
| 43Q | 210W | 0.12 | 0.03 | 0.01 | 9.3 | 0 | 0.83 | 0.12 |
| 69D | 210W | 0.15 | 0.06 | 0.01 | 9.3 | 0 | 0.56 | 0.17 |
| 41L | 122E | 0.30 | 0.22 | 0.01 | 9.3 | 0 | 0.43 | 0.49 |
| 118I | 122E | 0.20 | 0.11 | 0.01 | 9.2 | 0 | 0.53 | 0.24 |
| 70R | 219E | 0.12 | 0.03 | 0.01 | 9.1 | 0 | 0.12 | 0.78 |
| 39A | 210W | 0.16 | 0.07 | 0.01 | 9.0 | 0 | 0.50 | 0.19 |
| 74V | 181C | 0.17 | 0.04 | 0.02 | 8.9 | 0 | 0.35 | 0.25 |
| 60I | 219Q | 0.17 | 0.07 | 0.01 | 8.8 | 0 | 0.31 | 0.27 |
| 203K | 210W | 0.11 | 0.03 | 0.01 | 8.7 | 0 | 0.74 | 0.12 |
| 67N | 196E | 0.20 | 0.12 | 0.01 | 8.6 | 0 | 0.26 | 0.45 |
| 203K | 208Y | 0.20 | 0.02 | 0.02 | 8.6 | 0 | 0.45 | 0.26 |
| 215F | 218E | 0.16 | 0.03 | 0.02 | 8.6 | 0 | 0.22 | 0.41 |
| 118I | 211K | 0.20 | 0.13 | 0.01 | 8.4 | 0 | 0.65 | 0.22 |
| 67N | 228H | 0.13 | 0.05 | 0.01 | 8.4 | 0 | 0.13 | 0.67 |
| 43Q | 118I | 0.13 | 0.03 | 0.01 | 8.3 | 0 | 0.62 | 0.14 |
| 208Y | 211K | 0.11 | 0.06 | 0.01 | 8.3 | 0 | 0.86 | 0.11 |
| 70R | 228H | 0.13 | 0.05 | 0.01 | 8.2 | 2.22E-16 | 0.15 | 0.56 |
| 44D | 203K | 0.17 | 0.02 | 0.02 | 8.1 | 4.44E-16 | 0.21 | 0.45 |
| 41L | 69D | 0.12 | 0.06 | 0.01 | 8.0 | 8.88E-16 | 0.13 | 0.70 |
| 44D | 69D | 0.15 | 0.04 | 0.01 | 8.0 | 1.11E-15 | 0.26 | 0.28 |
| 41L | 43Q | 0.08 | 0.03 | 0.01 | 7.9 | 2.22E-15 | 0.08 | 0.92 |
| 35I | 83K | 0.16 | 0.07 | 0.01 | 7.9 | 2.66E-15 | 0.36 | 0.22 |
| 43E | 44A | 0.22 | 0.01 | 0.03 | 7.9 | 3.33E-15 | 0.23 | 0.95 |
| 181C | 221Y | 0.15 | 0.02 | 0.02 | 7.8 | 4.22E-15 | 0.17 | 0.64 |
| 100I | 103N | 0.10 | 0.01 | 0.01 | 7.8 | 4.88E-15 | 0.99 | 0.10 |
| 69D | 208Y | 0.15 | 0.03 | 0.02 | 7.8 | 5.11E-15 | 0.25 | 0.29 |
| 118I | 203K | 0.12 | 0.03 | 0.01 | 7.8 | 6.00E-15 | 0.13 | 0.54 |
| 103N | 181C | 0.14 | 0.06 | 0.01 | 7.8 | 6.88E-15 | 0.19 | 0.37 |
| 68G | 151M | 0.19 | 0.02 | 0.02 | 7.7 | 1.82E-14 | 0.24 | 0.45 |
| 44D | 211K | 0.12 | 0.07 | 0.01 | 7.6 | 2.29E-14 | 0.76 | 0.13 |
| 60I | 70R | 0.16 | 0.09 | 0.01 | 7.5 | 5.68E-14 | 0.39 | 0.21 |
| 215F | 228H | 0.15 | 0.04 | 0.01 | 7.5 | 9.15E-14 | 0.21 | 0.32 |
| 62V | 75I | 0.25 | 0.01 | 0.03 | 7.4 | 1.13E-13 | 0.33 | 0.51 |
| 67N | 219E | 0.09 | 0.03 | 0.01 | 7.4 | 1.20E-13 | 0.09 | 0.75 |
| 122E | 215Y | 0.28 | 0.22 | 0.01 | 7.4 | 1.21E-13 | 0.47 | 0.41 |
| 196E | 210W | 0.18 | 0.11 | 0.01 | 7.4 | 1.88E-13 | 0.36 | 0.26 |
| 43Q | 215Y | 0.08 | 0.03 | 0.01 | 7.4 | 1.91E-13 | 0.86 | 0.08 |
| 39A | 215Y | 0.13 | 0.08 | 0.01 | 7.3 | 2.17E-13 | 0.62 | 0.14 |
| 43E | 208Y | 0.15 | 0.03 | 0.02 | 7.3 | 3.04E-13 | 0.29 | 0.24 |
| 101E | 190A | 0.19 | 0.01 | 0.02 | 7.3 | 3.33E-13 | 0.54 | 0.23 |
| 210W | 219N | 0.07 | 0.02 | 0.01 | 7.2 | 4.84E-13 | 0.07 | 0.91 |
| 203K | 223E | 0.24 | 0.01 | 0.03 | 7.2 | 7.32E-13 | 0.25 | 0.89 |
| 39A | 67N | 0.13 | 0.07 | 0.01 | 7.2 | 7.59E-13 | 0.51 | 0.15 |
| 203K | 207E | 0.10 | 0.03 | 0.01 | 7.2 | 8.52E-13 | 0.53 | 0.11 |
| 122P | 177E | 0.09 | 0.03 | 0.01 | 7.1 | 1.25E-12 | 0.64 | 0.10 |
| 135T | 214L | 0.16 | 0.09 | 0.01 | 7.0 | 2.07E-12 | 0.20 | 0.41 |
| 122E | 123N | 0.07 | 0.03 | 0.01 | 7.0 | 2.42E-12 | 0.07 | 0.84 |
| 68G | 116Y | 0.17 | 0.01 | 0.02 | 7.0 | 2.66E-12 | 0.20 | 0.48 |
| 68G | 77L | 0.17 | 0.01 | 0.02 | 7.0 | 2.67E-12 | 0.20 | 0.53 |
| 203K | 215Y | 0.08 | 0.03 | 0.01 | 7.0 | 2.72E-12 | 0.82 | 0.08 |
| 41L | 184V | 0.32 | 0.26 | 0.01 | 7.0 | 3.10E-12 | 0.53 | 0.45 |
| 41L | 203K | 0.08 | 0.03 | 0.01 | 6.9 | 3.90E-12 | 0.08 | 0.81 |
| 69D | 219Q | 0.12 | 0.05 | 0.01 | 6.9 | 7.32E-12 | 0.33 | 0.16 |
| 118I | 214L | 0.14 | 0.07 | 0.01 | 6.8 | 7.52E-12 | 0.23 | 0.28 |
| 41L | 228H | 0.10 | 0.05 | 0.01 | 6.8 | 9.70E-12 | 0.11 | 0.70 |
| 118I | 219R | 0.09 | 0.02 | 0.01 | 6.8 | 1.11E-11 | 0.09 | 0.74 |
| 118I | 219N | 0.09 | 0.02 | 0.01 | 6.8 | 1.15E-11 | 0.09 | 0.71 |
| 20R | 219Q | 0.16 | 0.08 | 0.01 | 6.8 | 1.41E-11 | 0.27 | 0.27 |
| 62V | 151M | 0.19 | 0.01 | 0.03 | 6.7 | 1.51E-11 | 0.30 | 0.34 |
| 75M | 210W | 0.07 | 0.02 | 0.01 | 6.7 | 1.62E-11 | 0.80 | 0.07 |
| 208Y | 223Q | 0.15 | 0.01 | 0.02 | 6.7 | 2.20E-11 | 0.16 | 0.59 |
| 69N | 215F | 0.12 | 0.03 | 0.01 | 6.7 | 2.45E-11 | 0.30 | 0.17 |
| 43Q | 44D | 0.13 | 0.02 | 0.02 | 6.7 | 2.64E-11 | 0.36 | 0.16 |
| 20R | 67N | 0.17 | 0.11 | 0.01 | 6.6 | 2.93E-11 | 0.46 | 0.22 |
| 68G | 75I | 0.16 | 0.01 | 0.02 | 6.6 | 3.27E-11 | 0.19 | 0.47 |
| 69D | 215F | 0.12 | 0.04 | 0.01 | 6.6 | 4.12E-11 | 0.25 | 0.20 |
| 121Y | 122E | 0.06 | 0.02 | 0.01 | 6.6 | 5.08E-11 | 0.94 | 0.06 |
| 115F | 151M | 0.23 | 0.01 | 0.03 | 6.6 | 5.74E-11 | 0.58 | 0.28 |
| 121H | 122E | 0.06 | 0.02 | 0.01 | 6.5 | 7.59E-11 | 0.91 | 0.06 |
| 62V | 116Y | 0.19 | 0.01 | 0.03 | 6.5 | 8.38E-11 | 0.27 | 0.39 |
| 69D | 228H | 0.13 | 0.03 | 0.01 | 6.5 | 9.00E-11 | 0.21 | 0.25 |
| 67N | 122E | 0.24 | 0.19 | 0.01 | 6.5 | 9.56E-11 | 0.41 | 0.37 |
| 43E | 67N | 0.09 | 0.05 | 0.01 | 6.5 | 1.01E-10 | 0.58 | 0.10 |
| 20R | 70R | 0.17 | 0.10 | 0.01 | 6.4 | 1.35E-10 | 0.37 | 0.23 |
| 118I | 223Q | 0.08 | 0.01 | 0.01 | 6.4 | 1.39E-10 | 0.08 | 0.73 |
| 118I | 228H | 0.11 | 0.04 | 0.01 | 6.4 | 1.66E-10 | 0.14 | 0.36 |
| 210W | 219R | 0.06 | 0.02 | 0.01 | 6.4 | 1.94E-10 | 0.06 | 0.83 |
| 39A | 43E | 0.12 | 0.03 | 0.01 | 6.3 | 2.67E-10 | 0.16 | 0.29 |
| 181C | 210W | 0.12 | 0.07 | 0.01 | 6.3 | 2.91E-10 | 0.41 | 0.15 |
| 44D | 196E | 0.12 | 0.05 | 0.01 | 6.3 | 3.89E-10 | 0.34 | 0.15 |
| 108I | 181C | 0.11 | 0.02 | 0.01 | 6.2 | 4.15E-10 | 0.42 | 0.13 |
| 65R | 68G | 0.14 | 0.01 | 0.02 | 6.2 | 4.85E-10 | 0.48 | 0.17 |
| 208Y | 228H | 0.13 | 0.03 | 0.02 | 6.2 | 5.10E-10 | 0.22 | 0.23 |
| 44D | 122E | 0.11 | 0.07 | 0.01 | 6.2 | 5.21E-10 | 0.54 | 0.12 |
| 207E | 211K | 0.19 | 0.14 | 0.01 | 6.2 | 5.88E-10 | 0.58 | 0.22 |
| 44A | 118I | 0.07 | 0.01 | 0.01 | 6.2 | 6.04E-10 | 0.88 | 0.07 |
| 41L | 60I | 0.15 | 0.10 | 0.01 | 6.2 | 6.67E-10 | 0.17 | 0.53 |
| 41L | 75M | 0.05 | 0.02 | 0.01 | 6.2 | 6.83E-10 | 0.05 | 0.95 |
| 60I | 69D | 0.11 | 0.05 | 0.01 | 6.2 | 6.99E-10 | 0.16 | 0.27 |
| 60I | 218E | 0.10 | 0.03 | 0.01 | 6.1 | 8.05E-10 | 0.13 | 0.33 |
| 39A | 208Y | 0.11 | 0.04 | 0.01 | 6.1 | 9.62E-10 | 0.17 | 0.25 |
| 39A | 118I | 0.12 | 0.06 | 0.01 | 6.1 | 1.03E-09 | 0.29 | 0.17 |
| 67N | 104N | 0.05 | 0.02 | 0.01 | 6.1 | 1.19E-09 | 0.06 | 0.84 |
| 62V | 77L | 0.18 | 0.01 | 0.03 | 6.1 | 1.24E-09 | 0.24 | 0.38 |
| 44D | 219N | 0.11 | 0.01 | 0.02 | 6.1 | 1.37E-09 | 0.12 | 0.50 |
| 202V | 219Q | 0.11 | 0.05 | 0.01 | 6.0 | 1.55E-09 | 0.30 | 0.15 |
| 210W | 223Q | 0.06 | 0.02 | 0.01 | 6.0 | 1.60E-09 | 0.06 | 0.84 |
| 49R | 169D | 0.11 | 0.03 | 0.01 | 6.0 | 1.73E-09 | 0.20 | 0.21 |
| 44D | 219R | 0.11 | 0.01 | 0.02 | 6.0 | 1.81E-09 | 0.12 | 0.52 |
| 177E | 178M | 0.11 | 0.05 | 0.01 | 6.0 | 1.97E-09 | 0.13 | 0.36 |
| 210W | 228H | 0.10 | 0.05 | 0.01 | 6.0 | 2.17E-09 | 0.11 | 0.46 |
| 44D | 223Q | 0.11 | 0.01 | 0.02 | 6.0 | 2.20E-09 | 0.12 | 0.56 |
| 75M | 118I | 0.07 | 0.02 | 0.01 | 5.9 | 3.08E-09 | 0.58 | 0.08 |
| 75I | 115F | 0.23 | 0.01 | 0.04 | 5.9 | 3.09E-09 | 0.31 | 0.48 |
| 214L | 215F | 0.12 | 0.06 | 0.01 | 5.9 | 3.53E-09 | 0.18 | 0.25 |
| 67N | 203K | 0.07 | 0.03 | 0.01 | 5.9 | 3.84E-09 | 0.08 | 0.62 |
| 67N | 190A | 0.08 | 0.04 | 0.01 | 5.9 | 4.37E-09 | 0.09 | 0.56 |
| 135V | 162C | 0.12 | 0.05 | 0.01 | 5.9 | 4.37E-09 | 0.25 | 0.18 |
| 74V | 103N | 0.10 | 0.04 | 0.01 | 5.9 | 4.50E-09 | 0.35 | 0.13 |
| 208Y | 219R | 0.12 | 0.01 | 0.02 | 5.9 | 4.55E-09 | 0.14 | 0.45 |
| 39A | 44D | 0.11 | 0.04 | 0.01 | 5.9 | 4.79E-09 | 0.18 | 0.22 |
| 67N | 181C | 0.12 | 0.07 | 0.01 | 5.9 | 4.89E-09 | 0.14 | 0.47 |
| 208Y | 219N | 0.12 | 0.01 | 0.02 | 5.8 | 5.06E-09 | 0.14 | 0.45 |
| 115F | 116Y | 0.21 | 0.01 | 0.04 | 5.8 | 5.65E-09 | 0.47 | 0.28 |
| 44A | 210W | 0.05 | 0.01 | 0.01 | 5.8 | 5.72E-09 | 0.96 | 0.05 |
| 122E | 208Y | 0.09 | 0.05 | 0.01 | 5.8 | 5.88E-09 | 0.10 | 0.57 |
| 77L | 115F | 0.23 | 0.01 | 0.04 | 5.8 | 7.43E-09 | 0.31 | 0.45 |
| 203K | 223Q | 0.15 | 0.01 | 0.02 | 5.8 | 8.12E-09 | 0.20 | 0.42 |
| 41L | 219N | 0.05 | 0.02 | 0.00 | 5.8 | 8.37E-09 | 0.05 | 0.93 |
| 215Y | 219N | 0.05 | 0.02 | 0.00 | 5.8 | 8.66E-09 | 0.05 | 0.92 |
| 69D | 215Y | 0.10 | 0.06 | 0.01 | 5.7 | 1.07E-08 | 0.60 | 0.11 |
| 69D | 122E | 0.10 | 0.06 | 0.01 | 5.7 | 1.24E-08 | 0.53 | 0.11 |
| 67. | 69G | 0.80 | 0.00 | 0.14 | 5.7 | 1.31E-08 | 0.89 | 0.89 |
| 44D | 75M | 0.10 | 0.02 | 0.01 | 5.7 | 1.46E-08 | 0.11 | 0.42 |
| 207E | 210W | 0.16 | 0.11 | 0.01 | 5.7 | 1.56E-08 | 0.33 | 0.24 |
| 118I | 207E | 0.14 | 0.09 | 0.01 | 5.7 | 1.56E-08 | 0.26 | 0.23 |
| 118I | 196E | 0.14 | 0.08 | 0.01 | 5.6 | 1.61E-08 | 0.26 | 0.23 |
| 181C | 208Y | 0.10 | 0.04 | 0.01 | 5.6 | 1.68E-08 | 0.16 | 0.23 |
| 41L | 196E | 0.18 | 0.13 | 0.01 | 5.6 | 1.78E-08 | 0.22 | 0.49 |
| 104N | 219Q | 0.07 | 0.02 | 0.01 | 5.6 | 1.93E-08 | 0.56 | 0.07 |
| 39A | 228H | 0.11 | 0.04 | 0.01 | 5.6 | 1.93E-08 | 0.16 | 0.25 |
| 218E | 228H | 0.11 | 0.03 | 0.02 | 5.6 | 2.21E-08 | 0.22 | 0.18 |
| 207E | 215Y | 0.18 | 0.13 | 0.01 | 5.6 | 2.21E-08 | 0.49 | 0.22 |
| 35M | 39A | 0.10 | 0.03 | 0.01 | 5.6 | 2.25E-08 | 0.32 | 0.13 |
| 181C | 228H | 0.11 | 0.04 | 0.01 | 5.6 | 2.44E-08 | 0.16 | 0.24 |
| 210W | 228R | 0.06 | 0.02 | 0.01 | 5.5 | 3.25E-08 | 0.07 | 0.63 |
| 43E | 211K | 0.08 | 0.05 | 0.01 | 5.5 | 3.25E-08 | 0.73 | 0.08 |
| 101E | 181C | 0.09 | 0.01 | 0.01 | 5.5 | 3.36E-08 | 0.45 | 0.10 |
| 108I | 221Y | 0.16 | 0.01 | 0.03 | 5.5 | 3.86E-08 | 0.25 | 0.29 |
| 20R | 215F | 0.12 | 0.06 | 0.01 | 5.5 | 4.08E-08 | 0.18 | 0.28 |
| 40F | 43E | 0.12 | 0.01 | 0.02 | 5.4 | 5.28E-08 | 0.91 | 0.12 |
| 74V | 100I | 0.10 | 0.01 | 0.02 | 5.4 | 5.29E-08 | 0.11 | 0.49 |
| 67N | 219N | 0.05 | 0.02 | 0.01 | 5.4 | 5.33E-08 | 0.05 | 0.77 |
| 60I | 135T | 0.13 | 0.09 | 0.01 | 5.4 | 5.66E-08 | 0.37 | 0.17 |
| 103N | 108I | 0.07 | 0.02 | 0.01 | 5.4 | 6.23E-08 | 0.07 | 0.48 |
| 41L | 74I | 0.05 | 0.02 | 0.00 | 5.4 | 7.47E-08 | 0.05 | 0.86 |
| 39A | 69D | 0.10 | 0.04 | 0.01 | 5.4 | 7.48E-08 | 0.17 | 0.21 |
| 203D | 208Y | 0.10 | 0.02 | 0.02 | 5.4 | 7.63E-08 | 0.29 | 0.14 |
| 122E | 135T | 0.22 | 0.17 | 0.01 | 5.4 | 7.69E-08 | 0.32 | 0.41 |
| 28K | 32E | 0.48 | 0.00 | 0.09 | 5.4 | 7.78E-08 | 0.67 | 0.64 |
| 41L | 43N | 0.04 | 0.02 | 0.00 | 5.4 | 7.91E-08 | 0.04 | 0.94 |
| 215F | 219E | 0.09 | 0.03 | 0.01 | 5.3 | 9.91E-08 | 0.12 | 0.30 |
| 43N | 210W | 0.05 | 0.01 | 0.01 | 5.3 | 1.10E-07 | 0.73 | 0.05 |
| 75M | 208Y | 0.10 | 0.01 | 0.02 | 5.3 | 1.14E-07 | 0.35 | 0.12 |
| 208Y | 223E | 0.10 | 0.01 | 0.02 | 5.3 | 1.16E-07 | 0.11 | 0.65 |
| 20R | 69N | 0.10 | 0.04 | 0.01 | 5.3 | 1.38E-07 | 0.12 | 0.33 |
| 70R | 104N | 0.05 | 0.02 | 0.01 | 5.3 | 1.40E-07 | 0.05 | 0.65 |
| 69D | 203K | 0.09 | 0.02 | 0.01 | 5.3 | 1.50E-07 | 0.13 | 0.26 |
| *Negative correlations* | | | | | | | | |
| 210W | 214L | 0.01 | 0.09 | 0.00 | -50.2 | 0 | 0.01 | 0.02 |
| 214L | 215Y | 0.01 | 0.10 | 0.00 | -48.8 | 0 | 0.04 | 0.01 |
| 44D | 215F | 0.00 | 0.04 | 0.00 | -35.9 | 0 | 0.00 | 0.00 |
| 44D | 214L | 0.00 | 0.05 | 0.00 | -34.6 | 0 | 0.01 | 0.00 |
| 65R | 215Y | 0.00 | 0.02 | 0.00 | -33.7 | 0 | 0.01 | 0.00 |
| 41L | 214L | 0.03 | 0.11 | 0.00 | -25.8 | 0 | 0.03 | 0.10 |
| 70R | 210W | 0.04 | 0.13 | 0.00 | -24.5 | 0 | 0.07 | 0.07 |
| 162C | 166R | 0.00 | 0.05 | 0.00 | -24.1 | 0 | 0.00 | 0.01 |
| 121H | 123E | 0.00 | 0.02 | 0.00 | -23.4 | 0 | 0.01 | 0.00 |
| 122E | 123E | 0.06 | 0.16 | 0.00 | -22.6 | 0 | 0.09 | 0.13 |
| 210W | 219Q | 0.02 | 0.10 | 0.00 | -20.9 | 0 | 0.04 | 0.06 |
| 67N | 68G | 0.01 | 0.04 | 0.00 | -20.2 | 0 | 0.01 | 0.04 |
| 70R | 215Y | 0.07 | 0.16 | 0.00 | -19.3 | 0 | 0.18 | 0.10 |
| 215Y | 219Q | 0.04 | 0.11 | 0.00 | -19.0 | 0 | 0.05 | 0.14 |
| 210W | 211G | 0.00 | 0.02 | 0.00 | -18.4 | 0 | 0.00 | 0.01 |
| 60I | 62V | 0.00 | 0.02 | 0.00 | -17.0 | 0 | 0.00 | 0.01 |
| 83K | 142V | 0.01 | 0.06 | 0.00 | -16.4 | 0 | 0.01 | 0.03 |
| 210W | 215F | 0.02 | 0.07 | 0.00 | -16.0 | 0 | 0.02 | 0.07 |
| 83K | 214L | 0.02 | 0.08 | 0.00 | -15.7 | 0 | 0.03 | 0.05 |
| 162C | 214L | 0.01 | 0.07 | 0.00 | -15.3 | 0 | 0.03 | 0.03 |
| 208Y | 214L | 0.01 | 0.04 | 0.00 | -15.0 | 0 | 0.02 | 0.01 |
| 43E | 214L | 0.00 | 0.04 | 0.00 | -14.8 | 0 | 0.02 | 0.01 |
| 44D | 70R | 0.02 | 0.06 | 0.00 | -13.8 | 0 | 0.06 | 0.02 |
| 65R | 67N | 0.00 | 0.02 | 0.00 | -13.7 | 0 | 0.03 | 0.00 |
| 70R | 219N | 0.00 | 0.02 | 0.00 | -13.4 | 0 | 0.00 | 0.02 |
| 41L | 70R | 0.10 | 0.17 | 0.01 | -12.7 | 0 | 0.14 | 0.24 |
| 69N | 215Y | 0.01 | 0.05 | 0.00 | -12.7 | 0 | 0.12 | 0.02 |
| 162C | 219Q | 0.02 | 0.07 | 0.00 | -12.1 | 0 | 0.05 | 0.04 |
| 67N | 83K | 0.06 | 0.12 | 0.01 | -12.0 | 0 | 0.09 | 0.16 |
| 83K | 219Q | 0.03 | 0.08 | 0.00 | -11.9 | 0 | 0.06 | 0.06 |
| 151M | 215Y | 0.01 | 0.03 | 0.00 | -11.8 | 0 | 0.09 | 0.01 |
| 69N | 210W | 0.01 | 0.04 | 0.00 | -11.8 | 0 | 0.06 | 0.01 |
| 162A | 166R | 0.00 | 0.02 | 0.00 | -11.4 | 0 | 0.01 | 0.00 |
| 69D | 219E | 0.00 | 0.02 | 0.00 | -11.1 | 0 | 0.00 | 0.01 |
| 65R | 210W | 0.00 | 0.02 | 0.00 | -10.8 | 0 | 0.03 | 0.00 |
| 83K | 122E | 0.07 | 0.12 | 0.01 | -10.8 | 0 | 0.19 | 0.10 |
| 214L | 219N | 0.00 | 0.02 | 0.00 | -10.8 | 0 | 0.00 | 0.01 |
| 44D | 90I | 0.00 | 0.02 | 0.00 | -10.5 | 0 | 0.00 | 0.01 |
| 83K | 142T | 0.00 | 0.02 | 0.00 | -10.3 | 0 | 0.00 | 0.02 |
| 44D | 219Q | 0.01 | 0.05 | 0.00 | -10.3 | 0 | 0.04 | 0.02 |
| 178L | 219E | 0.00 | 0.02 | 0.00 | -10.2 | 0 | 0.00 | 0.01 |
| 215Y | 225H | 0.00 | 0.01 | 0.00 | -9.6 | 0 | 0.00 | 0.05 |
| 44D | 69N | 0.00 | 0.03 | 0.00 | -9.6 | 0 | 0.01 | 0.01 |
| 41L | 65R | 0.00 | 0.02 | 0.00 | -9.5 | 0 | 0.00 | 0.09 |
| 43E | 70R | 0.01 | 0.04 | 0.00 | -9.5 | 0 | 0.08 | 0.02 |
| 41L | 211G | 0.00 | 0.02 | 0.00 | -9.4 | 0 | 0.00 | 0.09 |
| 49R | 162A | 0.00 | 0.02 | 0.00 | -9.4 | 0 | 0.00 | 0.01 |
| 70R | 211K | 0.13 | 0.18 | 0.01 | -9.3 | 0 | 0.33 | 0.17 |
| 151M | 210W | 0.01 | 0.02 | 0.00 | -9.3 | 0 | 0.05 | 0.01 |
| 138A | 177E | 0.00 | 0.01 | 0.00 | -9.0 | 0 | 0.03 | 0.00 |
| 135M | 214L | 0.00 | 0.01 | 0.00 | -8.9 | 0 | 0.01 | 0.00 |
| 75I | 215Y | 0.00 | 0.02 | 0.00 | -8.8 | 0 | 0.10 | 0.00 |
| 98S | 162C | 0.01 | 0.05 | 0.00 | -8.7 | 0 | 0.04 | 0.02 |
| 41L | 151M | 0.01 | 0.03 | 0.00 | -8.7 | 0 | 0.01 | 0.12 |
| 178M | 202V | 0.01 | 0.04 | 0.00 | -8.5 | 0 | 0.02 | 0.02 |
| 35M | 142V | 0.00 | 0.03 | 0.00 | -8.4 | 0 | 0.01 | 0.01 |
| 158S | 208Y | 0.00 | 0.02 | 0.00 | -8.4 | 0 | 0.01 | 0.00 |
| 162C | 202V | 0.02 | 0.05 | 0.00 | -8.3 | 0 | 0.02 | 0.04 |
| 196E | 197E | 0.00 | 0.01 | 0.00 | -8.2 | 2.22E-16 | 0.00 | 0.03 |
| 83K | 118I | 0.04 | 0.09 | 0.01 | -8.0 | 1.55E-15 | 0.08 | 0.09 |
| 70R | 83K | 0.06 | 0.11 | 0.01 | -7.7 | 9.77E-15 | 0.10 | 0.14 |
| 211K | 214L | 0.07 | 0.11 | 0.01 | -7.6 | 2.44E-14 | 0.09 | 0.31 |
| 83K | 215F | 0.03 | 0.06 | 0.00 | -7.6 | 3.35E-14 | 0.04 | 0.08 |
| 41L | 219Q | 0.08 | 0.12 | 0.01 | -7.6 | 3.69E-14 | 0.10 | 0.25 |
| 67N | 69i | 0.00 | 0.02 | 0.00 | -7.5 | 4.95E-14 | 0.00 | 0.07 |
| 211K | 219Q | 0.08 | 0.12 | 0.01 | -7.5 | 7.33E-14 | 0.10 | 0.32 |
| 83K | 135T | 0.07 | 0.11 | 0.01 | -7.3 | 3.69E-13 | 0.17 | 0.11 |
| 116Y | 215Y | 0.01 | 0.02 | 0.00 | -7.2 | 6.10E-13 | 0.13 | 0.01 |
| 118I | 173Q | 0.00 | 0.01 | 0.00 | -7.1 | 1.39E-12 | 0.00 | 0.02 |
| 203K | 214L | 0.01 | 0.03 | 0.00 | -7.1 | 1.46E-12 | 0.03 | 0.01 |
| 21I | 219Q | 0.00 | 0.01 | 0.00 | -7.1 | 1.48E-12 | 0.02 | 0.00 |
| 211G | 215Y | 0.00 | 0.02 | 0.00 | -7.0 | 2.43E-12 | 0.12 | 0.01 |
| 41L | 83K | 0.09 | 0.13 | 0.01 | -7.0 | 2.91E-12 | 0.12 | 0.28 |
| 70R | 215D | 0.00 | 0.01 | 0.00 | -7.0 | 3.31E-12 | 0.00 | 0.03 |
| 215F | 219R | 0.00 | 0.02 | 0.00 | -7.0 | 3.51E-12 | 0.00 | 0.01 |
| 44D | 116Y | 0.00 | 0.02 | 0.00 | -6.9 | 5.55E-12 | 0.00 | 0.01 |
| 60I | 162C | 0.03 | 0.07 | 0.01 | -6.9 | 6.56E-12 | 0.06 | 0.06 |
| 70R | 219R | 0.00 | 0.02 | 0.00 | -6.9 | 7.14E-12 | 0.00 | 0.00 |
| 35L | 142V | 0.00 | 0.02 | 0.00 | -6.8 | 7.50E-12 | 0.01 | 0.00 |
| 48T | 83K | 0.00 | 0.02 | 0.00 | -6.8 | 1.08E-11 | 0.03 | 0.00 |
| 83K | 208Y | 0.02 | 0.05 | 0.00 | -6.8 | 1.17E-11 | 0.02 | 0.07 |
| 83K | 179I | 0.01 | 0.04 | 0.00 | -6.7 | 2.20E-11 | 0.02 | 0.06 |
| 207E | 211S | 0.00 | 0.01 | 0.00 | -6.7 | 2.36E-11 | 0.00 | 0.02 |
| 41L | 215V | 0.00 | 0.01 | 0.00 | -6.6 | 2.98E-11 | 0.00 | 0.07 |
| 75I | 210W | 0.00 | 0.02 | 0.00 | -6.6 | 5.20E-11 | 0.07 | 0.01 |
| 83K | 121H | 0.00 | 0.02 | 0.00 | -6.5 | 9.63E-11 | 0.00 | 0.04 |
| 83K | 219E | 0.01 | 0.03 | 0.00 | -6.5 | 1.03E-10 | 0.01 | 0.05 |
| 68G | 208Y | 0.01 | 0.03 | 0.00 | -6.4 | 1.32E-10 | 0.01 | 0.01 |
| 83K | 228H | 0.02 | 0.05 | 0.00 | -6.4 | 1.51E-10 | 0.03 | 0.07 |
| 162C | 215F | 0.03 | 0.06 | 0.00 | -6.4 | 2.12E-10 | 0.04 | 0.06 |
| 41L | 116Y | 0.01 | 0.02 | 0.00 | -6.3 | 3.65E-10 | 0.01 | 0.15 |
| 69D | 83K | 0.02 | 0.05 | 0.00 | -6.2 | 4.76E-10 | 0.08 | 0.03 |
| 60I | 174H | 0.00 | 0.01 | 0.00 | -6.2 | 5.77E-10 | 0.00 | 0.02 |
| 44D | 75I | 0.00 | 0.02 | 0.00 | -6.2 | 7.52E-10 | 0.00 | 0.01 |
| 178V | 210W | 0.00 | 0.01 | 0.00 | -6.1 | 9.61E-10 | 0.03 | 0.00 |
| 44A | 70R | 0.00 | 0.01 | 0.00 | -6.1 | 9.71E-10 | 0.05 | 0.00 |
| 75M | 214L | 0.00 | 0.02 | 0.00 | -6.0 | 2.07E-09 | 0.03 | 0.00 |
| 41L | 225H | 0.00 | 0.01 | 0.00 | -5.8 | 6.88E-09 | 0.00 | 0.10 |
| 166R | 169D | 0.01 | 0.03 | 0.00 | -5.7 | 1.12E-08 | 0.02 | 0.02 |
| 43Q | 214L | 0.01 | 0.03 | 0.00 | -5.7 | 1.37E-08 | 0.04 | 0.01 |
| 70R | 74V | 0.03 | 0.05 | 0.00 | -5.6 | 1.91E-08 | 0.03 | 0.12 |
| 69i | 184V | 0.01 | 0.02 | 0.00 | -5.6 | 1.97E-08 | 0.19 | 0.01 |
| 135T | 158S | 0.01 | 0.03 | 0.00 | -5.6 | 2.06E-08 | 0.01 | 0.11 |
| 39A | 43N | 0.00 | 0.01 | 0.00 | -5.6 | 2.54E-08 | 0.00 | 0.01 |
| 62V | 67N | 0.01 | 0.03 | 0.00 | -5.5 | 2.93E-08 | 0.13 | 0.01 |
| 21I | 70R | 0.00 | 0.01 | 0.00 | -5.5 | 3.38E-08 | 0.06 | 0.00 |
| 83K | 177N | 0.00 | 0.01 | 0.00 | -5.5 | 3.67E-08 | 0.00 | 0.03 |
| 60I | 174R | 0.00 | 0.01 | 0.00 | -5.4 | 6.50E-08 | 0.00 | 0.02 |
| 100I | 162C | 0.00 | 0.01 | 0.00 | -5.4 | 7.55E-08 | 0.03 | 0.00 |
| 98S | 178M | 0.01 | 0.04 | 0.00 | -5.4 | 8.11E-08 | 0.03 | 0.03 |
| 69D | 121Y | 0.00 | 0.01 | 0.00 | -5.3 | 9.13E-08 | 0.00 | 0.01 |
| 115F | 215Y | 0.00 | 0.01 | 0.00 | -5.3 | 1.06E-07 | 0.13 | 0.00 |
| 64R | 69D | 0.00 | 0.01 | 0.00 | -5.3 | 1.07E-07 | 0.01 | 0.00 |
| 83K | 207E | 0.06 | 0.09 | 0.01 | -5.3 | 1.20E-07 | 0.12 | 0.11 |

**^*^** 327 pairs from RTI experienced persons were significantly associated after adjusting for multiple comparisons by controlling the family-wise error rate at < 0.01

**^†^**J: Jaccard similarity coefficients; J_RAND_: expected value of the Jaccard similarity coefficient assuming Mut X and Mut Y occur independently based on a permutation procedure described in the methods; J_SE_ : the standard error of J; Z: (J- J_RAND_)/ J_SE_. Mutation pairs are ordered by their Z statistic.
