# Supplementary material for: HIV-1 Subtype B Protease and Reverse Transcriptase Amino Acid Covariation
Source: PLoS Comput Biol. 2007 May 11;3(5):e87. doi: 10.1371/journal.pcbi.0030087 (PMC1866358; doi:10.1371/journal.pcbi.0030087)
Supplement: Supplementary file 7 [file pcbi.0030087.st004.doc]

**Table S4. Highly Correlated HIV-1 Subtype B RT Mutation Pairs from RTI-Naive Persons**

| **Mut X** | **Mut Y** | **Association^†^** | | | | | **P(Y\|X)** | **P(X\|Y)** |
| --- | --- | --- | --- | --- | --- | --- | --- | --- |
|  |  | **J** | **J_RAND_** | **J_SE_** | **Z** | **p** |  |  |
| *Positive correlations* | | | | | | | | |
| 118I | 214L | 0.20 | 0.02 | 0.02 | 7.41 | 0.00 | 0.93 | 0.20 |
| 121H | 122E | 0.12 | 0.03 | 0.01 | 7.12 | 0.00 | 0.98 | 0.12 |
| 135V | 162C | 0.17 | 0.06 | 0.02 | 6.86 | 0.00 | 0.46 | 0.21 |
| 135T | 214L | 0.19 | 0.10 | 0.01 | 6.09 | 0.00 | 0.22 | 0.52 |
| 122E | 123N | 0.10 | 0.03 | 0.01 | 5.95 | 0.00 | 0.10 | 0.84 |
| 123E | 177E | 0.21 | 0.14 | 0.01 | 5.94 | 0.00 | 0.33 | 0.38 |
| 122Q | 177E | 0.09 | 0.02 | 0.01 | 5.44 | 0.00 | 0.83 | 0.09 |
| 122Q | 207E | 0.12 | 0.02 | 0.02 | 5.35 | 0.00 | 0.63 | 0.13 |
| 83K | 123E | 0.18 | 0.11 | 0.01 | 5.30 | 0.00 | 0.39 | 0.24 |
| *Negative correlations* | | | | | | | | |
| 122E | 123E | 0.05 | 0.16 | 0.01 | -17.66 | 0.00 | 0.09 | 0.10 |
| 83K | 142V | 0.01 | 0.05 | 0.00 | -15.49 | 0.00 | 0.01 | 0.02 |
| 122Q | 123E | 0.00 | 0.02 | 0.00 | -14.81 | 0.00 | 0.02 | 0.00 |
| 162C | 214L | 0.01 | 0.08 | 0.00 | -13.16 | 0.00 | 0.03 | 0.03 |
| 121H | 123E | 0.00 | 0.03 | 0.00 | -11.97 | 0.00 | 0.03 | 0.00 |
| 83K | 122E | 0.04 | 0.11 | 0.01 | -10.56 | 0.00 | 0.12 | 0.07 |
| 83K | 214L | 0.02 | 0.08 | 0.01 | -10.27 | 0.00 | 0.04 | 0.05 |
| 178M | 202V | 0.01 | 0.04 | 0.00 | -9.00 | 0.00 | 0.01 | 0.01 |
| 68G | 83K | 0.01 | 0.04 | 0.00 | -8.67 | 0.00 | 0.03 | 0.01 |
| 83K | 122Q | 0.00 | 0.02 | 0.00 | -8.51 | 0.00 | 0.00 | 0.02 |
| 162C | 166R | 0.01 | 0.05 | 0.00 | -8.50 | 0.00 | 0.02 | 0.04 |
| 142T | 207E | 0.00 | 0.02 | 0.00 | -8.35 | 0.00 | 0.01 | 0.00 |
| 178M | 214L | 0.01 | 0.05 | 0.01 | -7.83 | 0.00 | 0.03 | 0.02 |
| 48T | 83K | 0.00 | 0.02 | 0.00 | -7.74 | 0.00 | 0.02 | 0.00 |
| 118I | 162C | 0.00 | 0.02 | 0.00 | -7.04 | 0.00 | 0.00 | 0.00 |
| 135V | 177E | 0.02 | 0.06 | 0.01 | -6.89 | 0.00 | 0.09 | 0.03 |
| 122Q | 196E | 0.00 | 0.02 | 0.00 | -6.76 | 0.00 | 0.02 | 0.00 |
| 162C | 202V | 0.02 | 0.06 | 0.01 | -6.61 | 0.00 | 0.03 | 0.05 |
| 83K | 121H | 0.01 | 0.03 | 0.00 | -6.54 | 0.00 | 0.01 | 0.03 |
| 83K | 135T | 0.07 | 0.12 | 0.01 | -6.50 | 0.00 | 0.20 | 0.10 |
| 162C | 165I | 0.01 | 0.03 | 0.00 | -6.40 | 0.00 | 0.01 | 0.04 |
| 83K | 98S | 0.01 | 0.04 | 0.00 | -6.17 | 0.00 | 0.02 | 0.05 |
| 98S | 162C | 0.01 | 0.04 | 0.01 | -5.98 | 0.00 | 0.06 | 0.02 |
| 83K | 174H | 0.00 | 0.02 | 0.00 | -5.97 | 0.00 | 0.00 | 0.02 |
| 68G | 142V | 0.00 | 0.03 | 0.00 | -5.91 | 0.00 | 0.00 | 0.00 |
| 68G | 214L | 0.01 | 0.03 | 0.00 | -5.62 | 0.00 | 0.03 | 0.01 |
| 60I | 162C | 0.03 | 0.06 | 0.01 | -5.59 | 0.00 | 0.08 | 0.04 |
| 207E | 211G | 0.00 | 0.02 | 0.00 | -5.59 | 0.00 | 0.00 | 0.00 |
| 196E | 211K | 0.07 | 0.11 | 0.01 | -5.37 | 0.00 | 0.28 | 0.09 |
| 177E | 203D | 0.00 | 0.01 | 0.00 | -5.10 | 0.00 | 0.00 | 0.04 |
| 121Y | 123E | 0.00 | 0.02 | 0.00 | -5.05 | 0.00 | 0.07 | 0.00 |
| 83K | 123N | 0.01 | 0.03 | 0.00 | -5.04 | 0.00 | 0.01 | 0.05 |

**^†^** J: Jaccard similarity coefficients; J_RAND_: expected value of the Jaccard similarity coefficient assuming Mut X and Mut Y occur independently based on a permutation procedure described in the methods; J_SE_ : the standard error of J; Z: (J- J_RAND_)/ J_SE_. Mutation pairs are ordered by their Z statistic.
